# Peyer’s patch M cells organize an epithelial niche that sustains group 3 innate lymphoid cells and IL-22

**DOI:** 10.1038/s41590-026-02606-3

**Published:** 2026-08-14

**Authors:** Wang H. J. Cao, Yue You, Nancy Wang, M. Zeeshan Chaudhry, Huiyang Yu, Peter T. Bell, Ellesandra C. Noye, Renae Denman, Byungchul Lee, Abbey Waddington, Junpeng Ye, Jaring Schreuder, Qiutong Huang, Julie Tellier, Sophie Curio, James Santiago, Daniela Amann-Zalcenstein, Nicolas Jacquelot, Peter Hickey, Stephen L. Nutt, Cyril Seillet, Philip M. Hansbro, Verena C. Wimmer, Richard A. Strugnell, Zewen Kelvin Tuong, Matthew E. Ritchie, Gabrielle T. Belz

**Affiliations:** 1 The University of Queensland Frazer Institute, University of Queensland, Woolloongabba, Queensland Australia; 2 Walter and Eliza Hall Institute of Medical Research, Parkville, Victoria Australia; 3 Department of Medical Biology, University of Melbourne, Parkville, Victoria Australia; 4 Department of Microbiology and Immunology, The University of Melbourne at the Peter Doherty Institute for Infection and Immunity, Melbourne, Victoria Australia; 5 Department of Biochemistry & Molecular Biology; Department of Microbiology, Immunology & Infectious Diseases; Riddell Centre for Cancer Immunotherapy, Arnie Charbonneau Cancer Institute, Arthur J.E. Child Comprehensive Cancer Centre, Calvin, Phoebe, and Joan Snyder Institute for Chronic Diseases, Alberta Children’s Hospital Research Institute, Cumming School of Medicine, University of Calgary, Calgary, Alberta Canada; 6 Department of Immunology, University of Monash, Melbourne, Victoria Australia; 7 Centre for Inflammation, Centenary Institute and University of Technology Sydney, Faculty of Science, School of Life Sciences, Sydney, New South Wales Australia; 8 Ian Frazer Centre for Children’s Immunotherapy Research, Child Health Research Centre, Faculty of Medicine, The University of Queensland, Brisbane, Queensland Australia

**Keywords:** Innate lymphoid cells, Mucosal immunology

## Abstract

Microfold (M) cells transcytose luminal antigens to initiate mucosal adaptive immunity, but their role in organizing innate responses in Peyer’s patches is unclear. Here we showed that Peyer’s patch M cells organized an epithelial–group 3 innate lymphoid cell (ILC3) axis, establishing a spatial niche within the dome epithelium that drove ILC3 localization, proliferation and IL-22 production. We found that epithelial, but not hematopoietic, SPI-B was required for intestinal IgA responses to establish this niche. Single-cell profiling of intestinal SPI-B^+^ epithelial cells revealed that M cells were highly heterogeneous, displaying tissue- and pathogen-specific transcriptional programs. Using subset-specific genetic perturbation and whole-mount imaging, we found that this circuit relied on CCR6-dependent positioning cues and RANK–RANKL signaling to ILC3 to regulate Peyer’s patch ILC3 homeostasis. Together, these findings identified Peyer’s patch M cells as organizers that spatially coordinated innate cell localization, proliferation and cytokine production to maintain mucosal barrier defense.

## Abstract (web summary)

Belz and colleagues show that Peyer’s patch M cells are required for ILC3 localization, proliferation and IL-22 production within the dome epithelium.

## Main

Intestinal homeostasis depends on the coordinated interactions between epithelial and immune cells that sense luminal cues and tailor protective responses while preserving tissue integrity. The gut epithelium functions as both a physical barrier and as a signaling interface that integrates microbial, dietary and environmental cues to regulate innate and adaptive immune immunity^1^. This communication enables immune cells to balance tissue repair and barrier maintenance with protective responses; however, how specialized epithelial lineages spatially organize immune function within gut-associated lymphoid tissues remains incompletely understood.

The intestinal epithelium comprises multiple specialized cell types, including enterocytes, Paneth cells, goblet cells, enteroendocrine cells, Tuft cells and microfold (M) cells. Tuft cells are rare chemosensory cells that detect luminal metabolites and neuropeptides through the succinate receptor SUCNR1 (ref. ^2^) and the calcium activated channel TRPM5 (ref. ^3^). Through this sensing capacity, tuft cells establish an epithelial–group 2 innate lymphoid cell (ILC2) circuit promoting type 2 immunity, tissue repair and barrier defense^3,4^. Whether analogous epithelial circuits regulate other ILC populations, particularly within organized lymphoid structures such as Peyer’s patch, remains unknown.

M cells are specialized epithelial cells located within the follicle-associated epithelium of Peyer’s patches and along the intestinal epithelium^5^. They require SPI-B and SOX8 for functional differentiation and expression of canonical M cell markers^5,6^. M cells are classically defined by their capacity to transcytose luminal antigens and deliver them to underlying antigen-presenting cells to promote B cell activation and immunoglobulin A production^7^. Several enteric pathogens including *Salmonella* *enterica*, poliovirus and prions exploit the pathway to gain access to host tissues^8,9^. Furthermore, downstream nociceptor neurons can regulate M cell numbers in Peyer’s patches to limit *Salmonella* invasion and modulate gut microbial homeostasis^10^. This strategic position suggests tailored epithelial–immune interactions, but whether M cells coordinate immune organization beyond antigen transport remains unclear.

Here, we investigated how epithelial–immune interactions establish signaling circuits that relay luminal information to immune cells. Using SPI-B-tdTomato reporter mice, single-cell RNA sequencing and whole-mount imaging, we identified distinct M cell and tuft cell populations and defined their interactions with immune cells. M cells displayed tissue- and pathogen-specific transcriptional programs, whereas loss of SPI-B in M cells, but not tuft cells, disrupted ILC3 localization, proliferation and function within Peyer’s patches. We further identified Peyer’s patch M cells as organizers of a spatially restricted ILC3 niche regulated by CCR6-dependent positioning cues and RANK–RANKL signaling that controlled ILC3 abundance, IL-22 production and IgA-mediated mucosal immunity.

## Results

### Epithelial SPI-B is needed for intestinal IgA production

**Extended Data Fig. 1:**
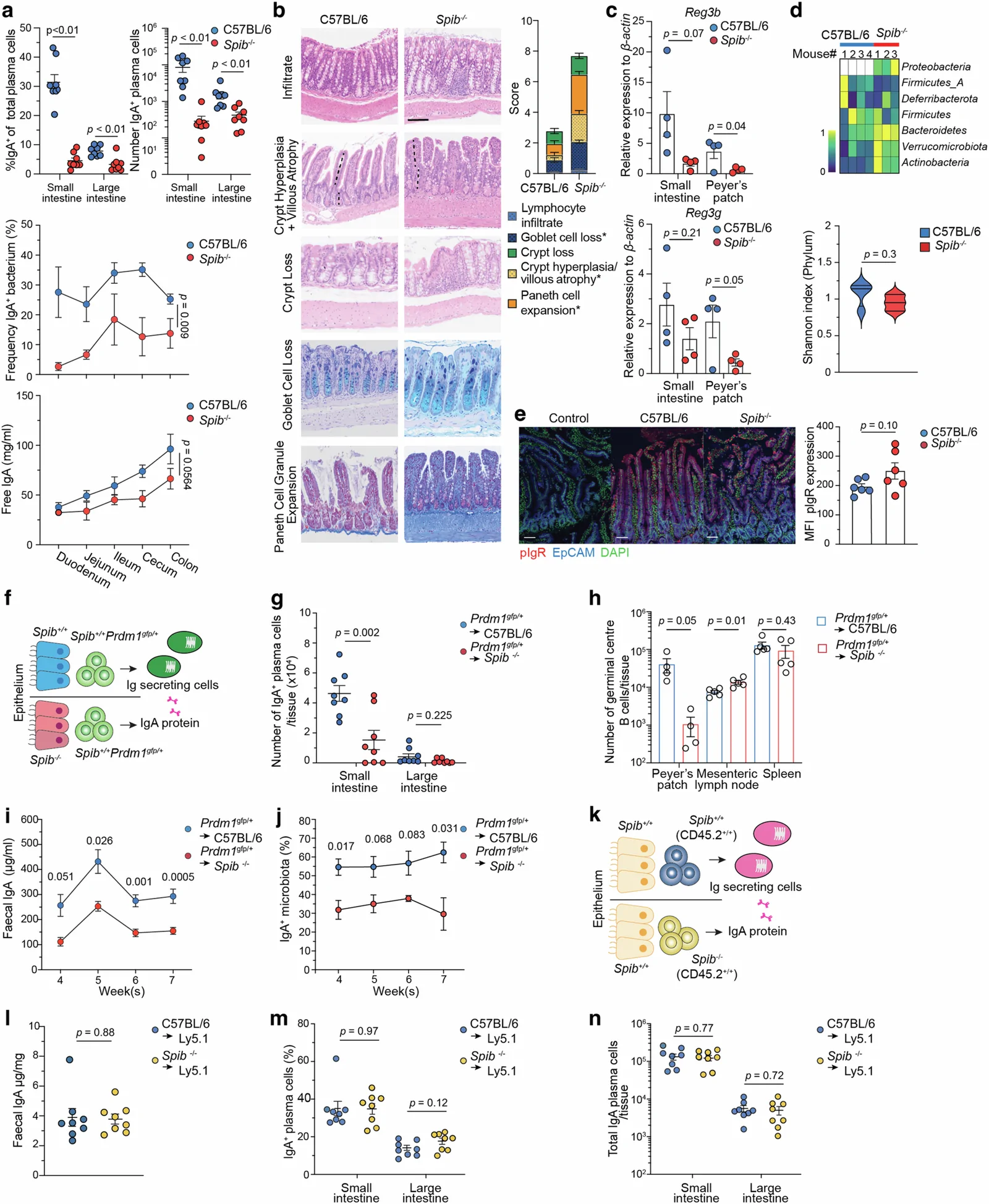
SPI-B-expressing epithelial cells are required for the generation of intestinal commensal- and pathogen-specific IgA responses. **a**, Frequency and number of IgA^+^ plasma cells in intestinal tissues (top panels) and frequency of IgA^+^ bacteria (middle panel) and quantification of IgA levels (bottom panel) in the luminal contents of small intestine (SI) and large intestine (LI) isolated from naïve C57BL/6 and *Spib*^*−/−*^ mice. Data pooled from two independent experiments is shown as mean ± s.e.m. (*n* = 8 mice/genotype). Statistical *p* values were determined using an unpaired two-tailed Student’s t-test (top) or a two-way ANOVA with the Geisser-Greenhouse correction (middle and bottom). **b**, Histological analysis (left) of small intestine epithelium stained with hematoxylin and eosin (H&E, infiltrate, crypt hyperplasia+villous atrophy, crypt loss), Alcian Blue Periodic Acid Schiff (goblet cells) or Masson Trichome (Paneth cells). Dashed line indicates villous length measurements. Scale bar, 100 μm. Quantitative analysis (right) of changes in intestinal morphology showing lymphocyte infiltration, goblet cell and crypt loss, crypt hyperplasia, villous atrophy and Paneth cell expansion in *Spib*^*−/−*^ and C57BL/6 mice. Data pooled from two experiments (*n* = 6 mice/genotype). Data show means ± s.e.m, p < 0.001, *. Statistical *p* value was determined using an unpaired two-tailed Student’s *t*-test. **c**, Quantitative PCR analysis of *Reg3b* and *Reg3g* gene expression in SI and Peyer’s patch (PP) of naïve C57BL/6 and *Spib*^*−/−*^ mice. Data pooled from two experiments shown as mean ± s.e.m. (*n* = 4 mice/genotype). Statistical *p* value was determined using an unpaired two-tailed Student’s *t*-test. **d**, Abundance of bacterial phyla (top) and Shannon index (bottom) for microbial flora in feces of naïve C57BL/6 and *Spib*^*−/−*^ mice. Not-detected bacterial phyla indicated as white boxes. Data from one experiment (*n* = 3 or 4 mice/genotype). Statistical *p* value was determined using an unpaired two-tailed Student’s *t*-test. **e**, Representative pIgR staining (left) in SI villi of C57BL/6 and *Spib*^*−/−*^ mice. Scale bar, 50 μm. Immunofluorescence imaging quantification of pIgR expression as MFI (right) in EpCAM^+^ IECs. Data from two independent experiments shown as mean ± s.e.m. (*n* = 6 mice/genotype). Statistical significance was determined using an unpaired two-tailed Student’s *t*-test. **f**, Schematic showing bone marrow chimeras generation where C57BL/6 and *Spib*^*−/−*^ mice were transplanted with bone marrow (BM) cells from *Prdm1*^gfp/+^ mice following lethal irradiation. Fecal samples were collected from week 3 to week 7 after BM reconstitution. **g**, Total number of IgA^+^CD98^+^BLIMP-GFP^+^ plasma cells (left) isolated from the SI and LI chimeric C57BL/6 and *Spib*^*−/−*^ mice at 8 weeks after BM reconstitution as in **f**. **h**, Total number of germinal center B cells (Live CD19^+^B220^+^CD95^+^CD38^−^) isolated from SI, LI and mesenteric lymph nodes (mLN) of chimeric C57BL/6 and *Spib*^*−/−*^ mice as in **g**. **g,h**, Data pooled from four independent experiments shows as mean ± s.e.m. (*n* = 8 mice/genotype). Panel **h** shows individual mice for SI and LI and pooled samples for PP (2 mice/sample). Statistical *p* values were determined using an unpaired two-tailed Student’s t-test. **i**, ELISA quantification of fecal IgA from chimeric C57BL/6 and *Spib*^*−/−*^mice as in **f** at the indicated time points. **j**, Frequency of IgA^+^ gut microbiota (SYTO9-Green^+^PI^−^ events) in fecal samples from chimeric C57BL/6 and *Spib*^*−/−*^mice at indicated time points as in **f**. **i,j**, Data pooled from three independent experiments shown as mean ± s.e.m (*n* = 8 mice/genotype). **k**, Schematic showing where Ly5.1 mice were transplanted with BM cells from congenic C57BL/6 (CD45.2^+^) *or Spib*^*+/+*^ (CD45.2^+^) mice following lethal irradiation. **l**-**n** Quantification of fecal IgA (**l**), frequency (**m**) and total number (**n**) of IgA^+^CD98^+^ plasma cells isolated from the SI and LI of chimeric Ly5.1 mice at 8 weeks after BM reconstitution as in **k**. Data pooled from three independent experiments is shown as mean ± s.e.m. (*n* = 8 mice/genotype). Statistical significance was determined using an unpaired two-tailed Student’s *t-*test. Source Data

SPI-B is expressed in both hematopoietic and epithelial compartments, including B cells, T cells^11^, plasmacytoid dendritic cells (DCs)^12^, stromal cells^13^ and intestinal epithelial cells (IECs)^5^. Consistent with previous studies^11^, *Spib*^−/−^ mice exhibited marked defects in Peyer’s patch development and reduced IgA production (Extended Data Fig. 1a). In addition, *Spib*^−/−^ mice exhibited reduced fecal IgA and decreased IgA coating of commensal bacteria and IgA^+^ plasma cells, impaired expression of antimicrobial molecules and reduced microbial diversity (Extended Data Fig. 1a–d), together with an increase in Paneth cells, villus number and length and crypt hyperplasia (Extended Data Fig. 1a–d). These changes occurred despite preserved polymeric immunoglobulin receptor (pIgR) expression (Extended Data Fig. 1e), indicating impaired IgA transcytosis was unlikely to explain the phenotype.

**Extended Data Fig. 2:**
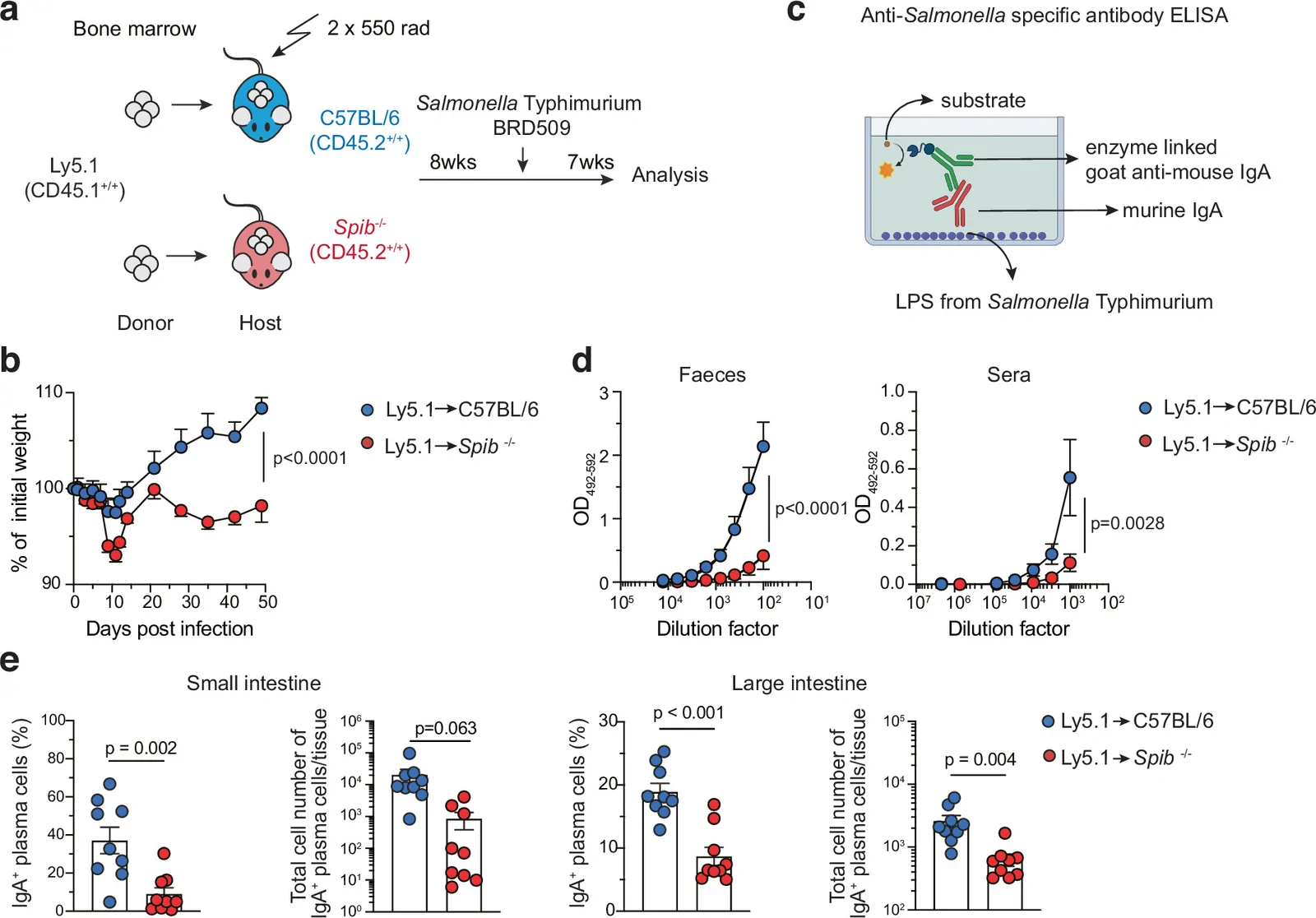
SPI-B deficiency in epithelial cells impairs barrier function during infection. **a**, Schematic showing generation of bone marrow chimeric mice where C57BL/6 (CD45.2^+^) and *Spib*^*−*/*−*^ (CD45.2^+^) mice were reconstituted with congenic Ly5.1 (CD45.1^+^) BM after lethal irradiation. Mice were infected with *Salmonella enterica* serovar Typhimurium BRD509 8 weeks after BM reconstitution. **b**, Body mass change in chimeric C57BL/6 and *Spib*^*−*/*−*^ mice following the *Salmonella enterica* serovar Typhimurium BRD509 infection as in **a**. **c**, Schematic diagram showing the principle of the *Salmonella*-specific IgA ELISA. The ELISA plate was pre-coated with *Salmonella*-derived LPS and then incubated with serum or fecal samples. **d**, ELISA quantification of relative amounts of LPS-specific IgA in feces and sera of chimeric C57BL/6 and *Spib*^*−*/*−*^ mice as in **a** at 7 weeks after infection with *Salmonella enterica* serovar Typhimurium BRD509. **e**, Flow cytometric quantification of frequency and total number of IgA^+^CD98^+^ plasma cells isolated from the small and large intestine of chimeric C57BL/6 and *Spib*^*−*/*−*^ mice as in **d**. **b**, **d**, **e** Data pooled from three independent experiments shown as mean ± s.e.m. (*n* = 9 mice/genotype). Statistical *p* values were determined using a two-way ANOVA (**b**, **d**) or an unpaired two-tailed Student’s *t*-test (**e**). Source Data

To determine whether these effects reflected loss of SPI-B in the hematopoietic or epithelial compartments, we generated bone-marrow (BM) chimeras using *Prdm1*^*gfp/+*^reporter mice, which express green fluorescent protein (GFP) in plasma cells and plasmablasts^14^. Reconstitution of lethally irradiated *Spib*^−/−^ or C57BL/6 mice with *Prdm1*^*gfp/+*^ BM cells resulted in a pronounced reduction in IgA^+^CD45^+^BLIMP1-GFP^+^CD98^+^ plasma cells in the small intestine and fewer germinal center B cells in Peyer’s patches, accompanied by decreased fecal IgA (Extended Data Fig. 1f–h), reduced IgA coating of commensal bacteria (Extended Data Fig. 1i–k) and enhanced tissue damage (Supplementary Fig. 1). In contrast, chimeric wild-type recipients reconstituted with *Spib*^−/−^ BM exhibited normal frequencies of intestinal IgA^+^ plasma cells and intact IgA production (Extended Data Fig. 1k–n). Notably, germinal center B cell populations in the spleen and mesenteric lymph nodes were maintained across all chimeric settings (Extended Data Fig. 1h), indicating that the requirement for SPI-B was restricted to the intestinal epithelium. Similarly, IgA^+^ plasma cells were reduced in the small and large intestine of *S.* *enterica* serovar Typhimurium-infected *Spib*^−/−^ or C57BL/6 mice reconstituted with wild-type (Ly5.1^+^) BM (Extended Data Fig. 2). Thus, epithelial, rather than hematopoietic, SPI-B was required for maintaining intestinal IgA responses.

### SPI-B reveals distinct M cell and Tuft cell populations

**Extended Data Fig. 3:**
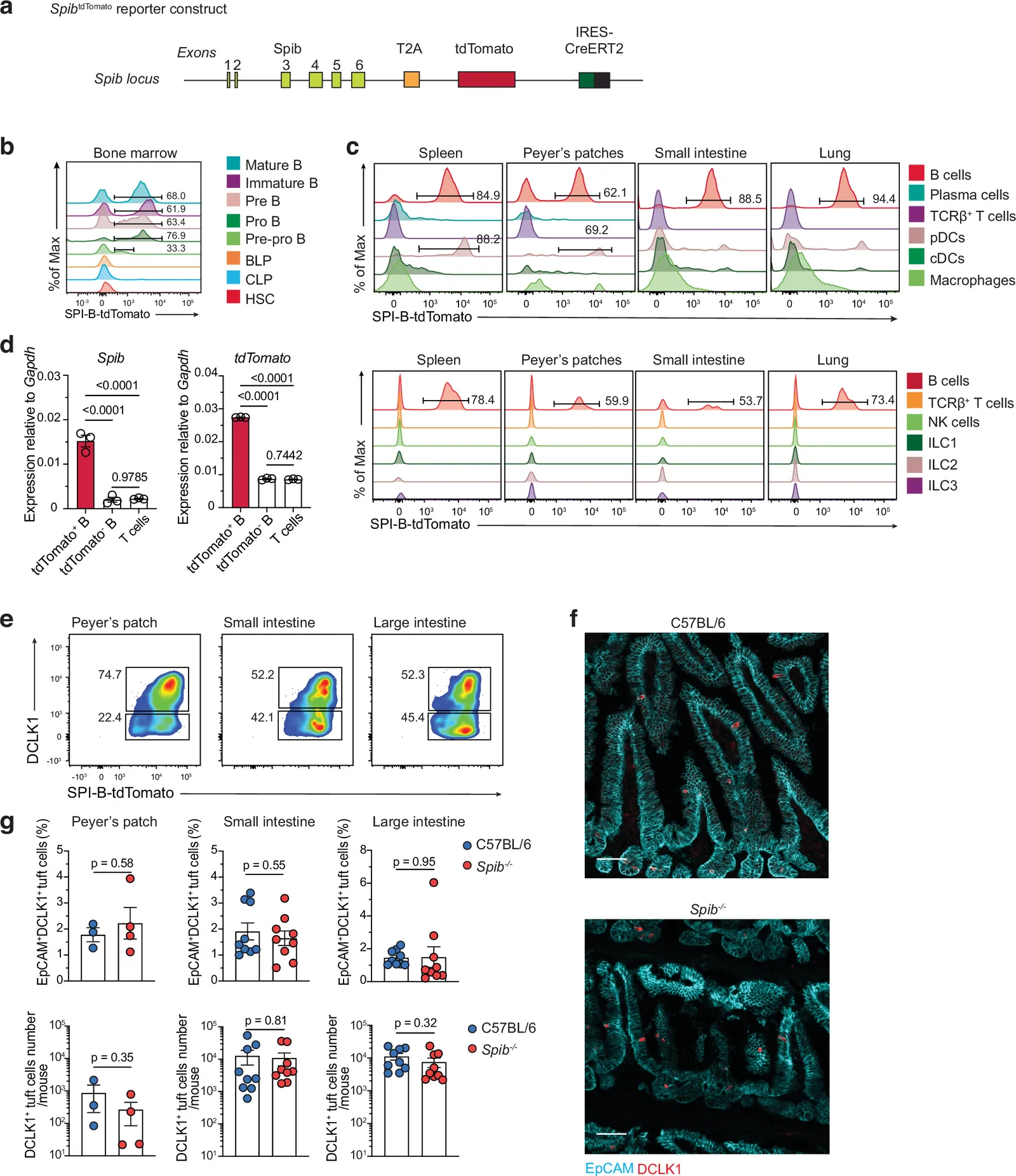
SPI-B-tdTomato expression identifies immune and epithelial cell populations. **a**, Schematic diagram showing the genomic construct design for the generation of the *Spib*^*−2A−*tdTomato*−*CreERT2^ reporter strain. **b**, Flow cytometric plots showing the gating strategy for progenitor and mature B cells isolated from the bone marrow of *Spib*^tdTomato*−*CreERT2^ (*Spib*^tdTomato/+^) mice (*n* = 3 mice from two similar experiments). Lineage markers included live/dead, CD3ε, CD4, CD8α, NKp46, CD11b, CD11c, Ly6G/Ly6C and TER119. Histograms (right) show SPI-B-tdTomato expression for different B cell developmental stages in bone marrow and the frequency of tdTomato^+^ cells. **c**, SPI-B-tdTomato expression by different immune cell types isolated from the spleen, Peyer’s patches and small intestine lamina propria and lung of *Spib*^tdTomato/+^ mice. Numbers indicate frequency of tdTomato^+^ cells within each population. Data show representative plots from one of five individual *Spib*^*tdTomato/+*^ mice from three independent experiments (*n* = 5 mice) with similar results. Cell populations were gated as live B cells: CD19^+^ B220^+^; plasma cell: CD138^+^ CD98^+^; T cells: CD3ε^+^TCRβ^+^; pDCs: CD11c^+^ SiglecH^+^; cDCs: CD64^−^CD11c^+^MHCII^+^; macrophages: CD11b^+^CD64^+^; NK cells: CD3ε^*−*^CD19^−^NK1.1^+^NKp46^+^CD49b^+^; ILC1: CD3ε^*−*^CD19^−^NK1.1^+^NKp46^+^CD49a^+^; ILC2: CD3ε^*−*^CD19^−^NK1.1^*−*^NKp46^−^KLRG1^+^; and ILC3: CD3e^−^CD19^−^NK1.1^*−*^ c-Kit^+^. **d**, Quantitative PCR analysis for the indicated transcripts of live (PI^−^) populations of cells from spleen purified based on their expression of SPI-B-tdTomato. Data show the mean ± s.e.m. of three animals (*n* = 3 mice). Statistical *p* values were determined using an unpaired two-tailed Student’s *t*-test. **e**, Flow cytometric intensity plots showing anti-DCLK1 staining of CD45-EpCAM^+^ SPI-B-tdTomato^+^ intestinal epithelial cells from the Peyer’s patch, small intestine, and large intestine in *Spib*^tdTomato/+^ mice. Data show representative plots from one of nine individual mice analyzed and number indicates frequency within the CD45-EpCAM^+^ SPI-B-tdTomato^+^ gate with similar results from three independent experiments. **f**, Confocal images of 7 μm fixed-frozen small intestinal tissue stained for EpCAM (epithelial cells, cyan) and DCLK1 (tuft cells, red). Representative images from one of four mice/genotype, scale bar, 100 μm. **g**, Frequency and number of EpCAM^+^DCLK1^+^ cells within the CD45^*−*^EpCAM^+^ gate. Data show pooled samples for Peyer’s patches (C57BL/6, *n* = 3; *Spib*^*−*/*−*^, *n* = 4), and individual values for small and large intestine. Data show mean ± s.e.m. of one representative (C57BL/6, *n* = 9 mice; *Spib*^*−*/*−*^, *n* = 8 mice) of two independent experiments. Statistical differences were determined using an unpaired two-tailed Student’s *t*-test. Source Data

M cells have been difficult to study due to their rarity and lack of unique surface markers. UEA-1 and NKM-16-2-4 antibodies that recognize fucose carbohydrate moieties, peptidoglycan recognition protein-S (PGRP-S) and CD155 reporters are confounded by variable specificity, particularly under inflammatory conditions^15–17^. To resolve epithelial populations expressing SPI-B, we generated a *Spib*^*−2A−tdTomato−IRES−c*^^*reERT2*^ reporter mouse (referred to as *Spib*^*tdTomato*^, Extended Data Fig. 3a), which allows the visualization and isolation of SPI-B-expressing cells. SPI-B-tdTomato expression was detected in CD45^*−*^EpCAM^+^ IECs and CD19^+^ B cells, but not in CD98^+^CD138^+^ plasma cells or CD19^*−*^CD3ε^*−*^CD90.2^+^IL-7Rα^+^ ILCs (Extended Data Fig. 3b–e). Within the intestinal epithelium, approximately 50% of SPI-B-tdTomato^+^EpCAM^+^ cells coexpressed the tuft cell marker DCLK1 (mean ± s.e.m. Peyer’s patch, 70.93 ± 4.73%; small intestine, 69.83 ± 2.89% and large intestine, 48.67 ± 4.70%; Extended Data Fig. 3e) whereas the remaining DCLK^*−*^ population exhibited features consistent with M cells (Extended Data Fig. 3e). Both populations were detected throughout the small and large intestine and in Peyer’s patches (Extended Data Fig. 3e). While M cells were reported to be absent in *Spib*^−/−^ mice^5^, we found that tuft cells were present at comparable frequencies in wild-type and *Spib*^−/−^ mice (Extended Data Fig. 3f,g), indicating a requirement for SPI-B in M cell but not tuft cell development.

**Fig. 1:**
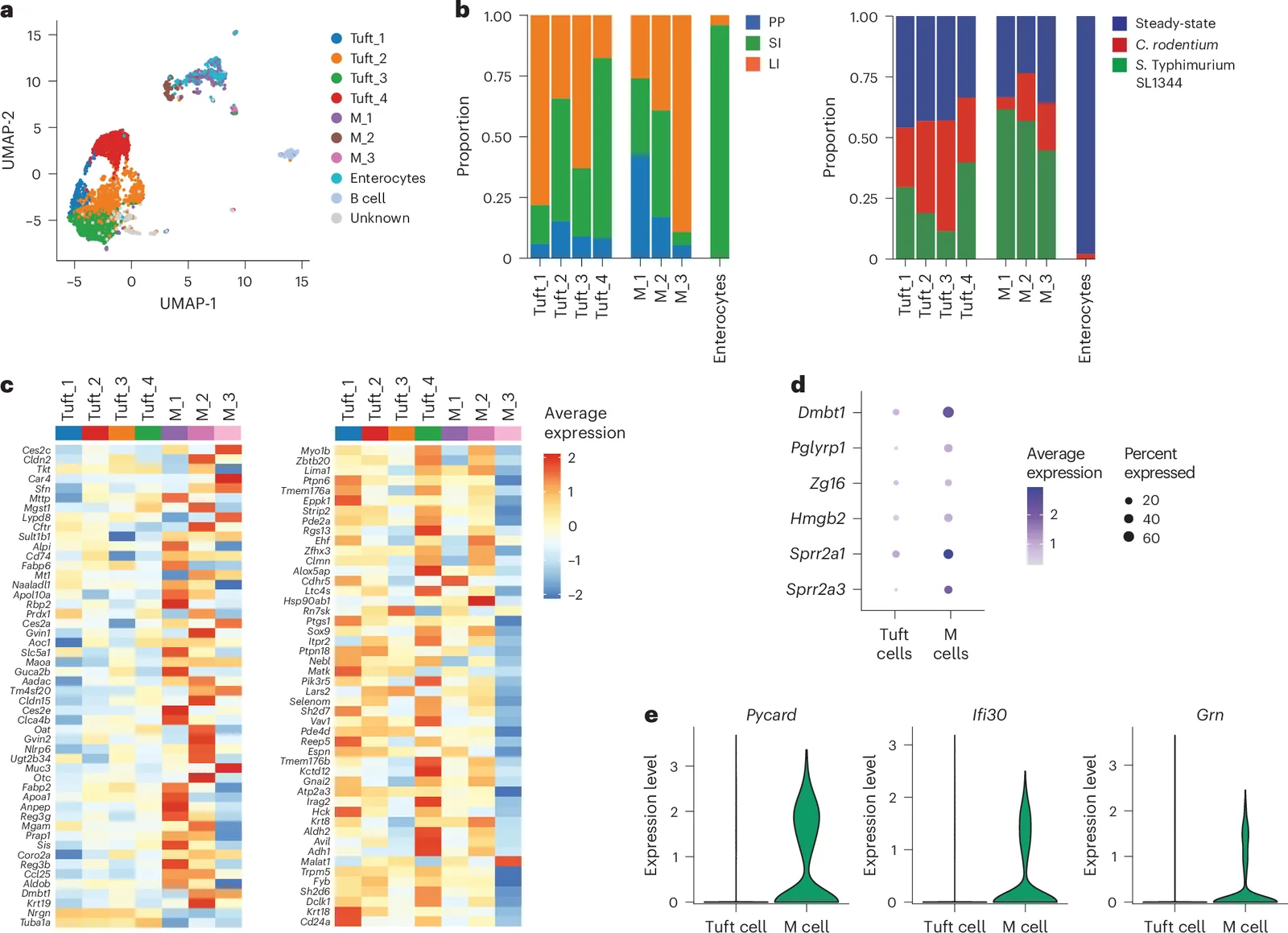
scRNA-seq identifies distinct SPI-B^+^ epithelial cells across steady-state and infection conditions. **a**, Uniform Manifold Approximation and Projection (UMAP) showing single-cell transcriptional profile of SPI-B-tdTomato^+^ IECs pooled from Peyer’s patches (PPs), small intestine (SI) and large intestine (LI) of *Spib*^*tdTomato/+*^ mice at steady state or on day 3 after infection with *S.* *enterica* serovar Typhimurium SL1344 or *C.* *rodentium*. **b**, Relative proportions of SPI-B-tdTomato^+^ Tuft_1–Tuft_4, M_1–M_3 cells and enterocytes as in **a** in wild-type mice based on anatomical location (in the PPs, SI and LI; left) or infection status (steady state, *S.* *enterica* serovar Typhimurium SL1344 or *C.* *rodentium*; right). **c**, Heatmap showing average expression of the top 40 differentially expressed genes (DEGs) in Tuft_1–Tuft_4 and M_1–M_3 cells isolated from PPs, SI and LI of *Spib*^*tdTomato/+*^ mice as in **a**. **d**, Expression of genes encoding antimicrobial peptides (*Pglyrp1*, *Hmgb2*, *Sprr2a1*, *Sprr2a3*) or related proteins (*Dmbt1*, *Zg16*) in tuft cells and M cells as in **c**. Dot size represents the percentage of cells expressing each gene; color indicates the average expression of each gene. **e**, Violin plots showing expression of *Pycard*, *Ifi30* and *Grn* in tuft cells and M cells as in **c**. **a**–**e** show data from one experiment (*n* = 5 mice for naive, 3 mice for *S*. Typhimurium SL1344 and 3 mice for *C.* *rodentium*). Source Data

Tissue- and infection-specific epithelial states were resolved by single-cell RNA sequencing (scRNA-seq) of CD45^*−*^EpCAM^+^SPI-B-tdTomato^+^ and SPI-B-tdTomato^*−*^ epithelial cells sorted from *Spib*^*tdTomato/+*^ mice at steady state or at day 3 of infection with *S.* Typhimurium, which initially colonizes the small intestine, or day 3 following infection with *Citrobacter* *rodentium*, which colonizes the cecum and colon (Fig. 1a,b and Supplementary Table 1). CD45^*−*^EpCAM^+^SPI-B-tdTomato^+^ and CD45^−^EpCAM^+^SPI-B-tdTomato^*−*^ epithelial cells were index-sorted for lineage-resolved analysis across tissues and conditions. To maximize discovery of cell-type and cell-state profiles, scRNA-seq data from each condition were combined before deconvolution to define tissue- and pathogen-specific features. This analysis therefore included Peyer’s patch and villous M cells, and potentially M cells from small isolated tertiary lymphoid follicles^18^, which could not be separated. After quality control filtering (removal of cells with a high percentage of mitochondrial genes, a low total gene content and doublets) 3,262 SPI-B^+^ epithelial cells were retained for downstream analysis (Supplementary Tables 2 and 3) and gene expression of SPI-B-tdTomato^+^ IECs was compared to SPI-B-tdTomato^−^ IECs.

**Extended Data Fig. 4:**
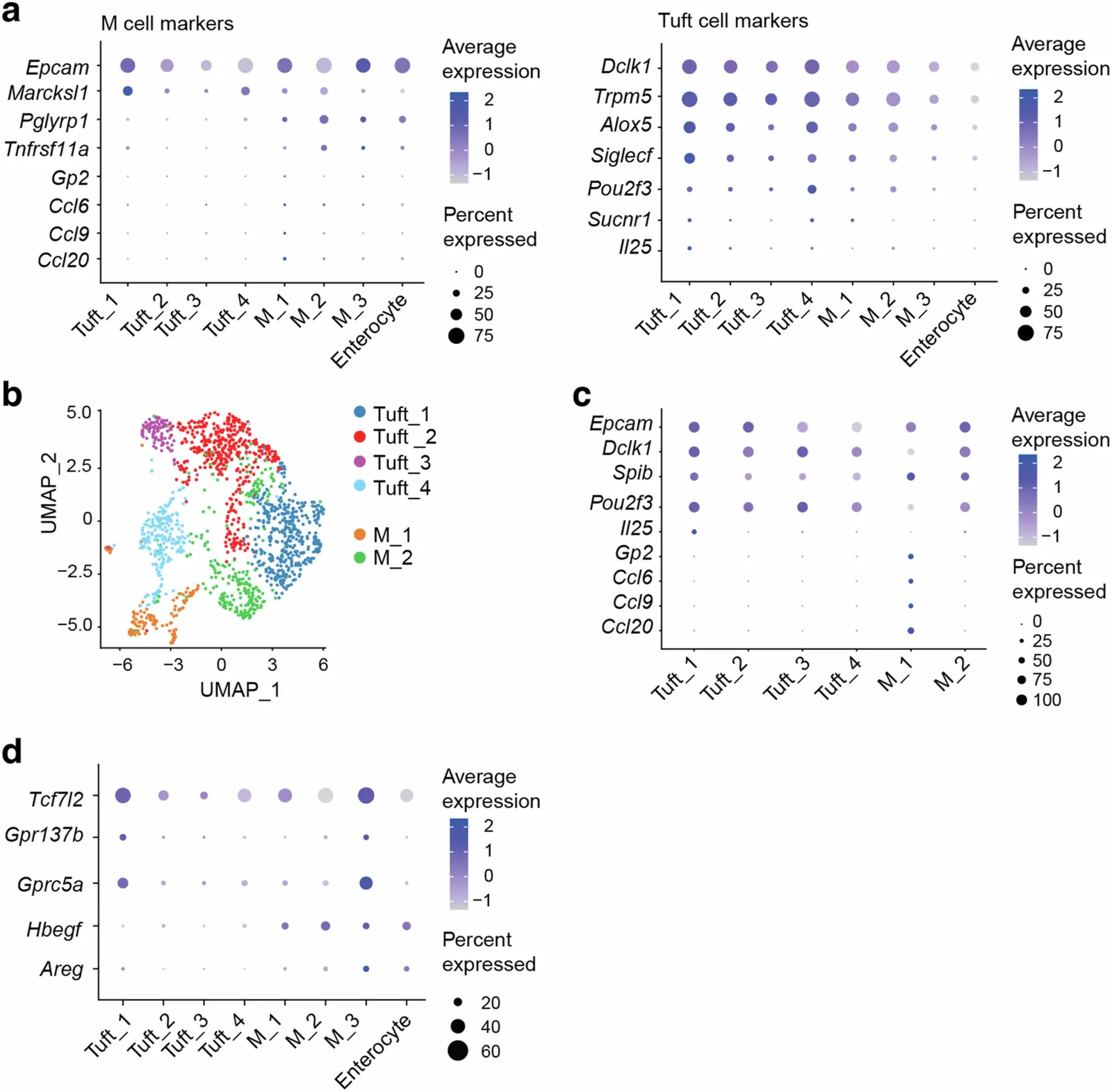
M cell clusters exhibit distinct transcriptional programs at steady-state and during enteric infection. **a**, Dot plots showing average expression of canonical markers of tuft cells, M cells and enterocytes in each cluster within SPI-B-tdTomato^+^ IECs isolated from naïve *Spib*^*tdTomato/+*^ mice or 3 days after infection with *Salmonella enterica* serovar Typhimurium SL1344 or *Citrobacter rodentium*. Data from one experiment (*n* = 5 naïve mice, *n* = 3 mice for *S. enterica* Typhimurium SL1344 and *n* = 3 mice for *C. rodentium*). Dot size represents the frequency of cells expressing each gene, while color indicates average gene expression. **b**, UMAP showing scRNA-seq based transcriptional profile of SPI-B-tdTomato^+^ IECs isolated from Peyer’s patches of naïve *Spib*^*tdTomato/+*^*Rorc*^*gfp/+*^ mice. Data from one experiment, cells pooled from 10 mice. **c**, Dot plot showing expression of canonical M cell markers across Tuft_1-Tuft_4 and M_1 and M_2 cells as in **b**. Dot size represents the percentage of cells expressing each gene and color indicates average expression. **d**, Dot plot showing selected gene expression across Tuft_1-Tuft_4, M_1-M_3 cells and enterocytes as in **a**.

Unsupervised clustering of SPI-B-tdTomato^+^ IECs resolved seven transcriptionally distinct clusters comprising four tuft cell clusters and three M cell clusters (Fig. 1a and Extended Data Fig. 4a). Tuft cell populations expressed canonical markers, including *Dclk1*, *Trpm5* and *Alox5*, and were annotated as Tuft_1 (*Sucnr1*, *Dclk1* and *Il25*), Tuft_2 (*Pou2f3*, *Il13ra* and *Dclk1*), Tuft_3 (*Trpm5*, *Ptpn18* and *Plcb*2) and Tuft_4 (*Chat* and *Cox1*) (Supplementary Table 1). All tuft cell clusters were represented across the different intestinal regions and infection states with Tuft_1 and Tuft_3 corresponding to previously described Tuft_2 and Tuft_1 populations^19–22^, respectively (Supplementary Table 1). Tuft cell clusters showed distinct tissue distributions, with Tuft_1 and Tuft_3 cells predominantly found in the large intestine (78.2% and 63.1%, respectively), whereas Tuft_2 and Tuft_4 cells were more prevalent in the small intestine (50.4% and 74.2%, respectively) (Fig. 1b). The three M cell clusters expressed canonical M cell genes, including *Pglyrp1*, *Tnfrsf11a* and *Gp2* (Extended Data Fig. 4a). M_1 cells (*Ccl25*, *Fabp1*, *H2-Ab1*, *Reg3b*, *Reg3g* and *CD74*) were most abundant in Peyer’s patch (42.6%), M_2 cells (*Pycard, Cd81, Mki67, Irf6 and Irf8*) in the small intestine (43.8%) and the M_3 cells (*Sprr2b, Saa1, Hk2, Foxo3, Dmbt1, Pdgfa and Pglyrp1*) were almost exclusively found in the large intestine (89.3%) (Fig. 1b and Supplementary Table 1). Notably, these populations were substantially amplified in *S.* *Typhimurium*-infected mice (Fig. 1b). scRNA-seq of Peyer’s patch M cells from naive *Spib*^*tdTomato*^ mice revealed that M_1 and M_2 cells, but not M_3 cells, could be resolved consistent with the predominance of M_3 cells in the large intestine and their enrichment following enteric infection (Fig. 1b, Extended Data Fig. 4b,c and Supplementary Table 4).

**Fig. 2:**
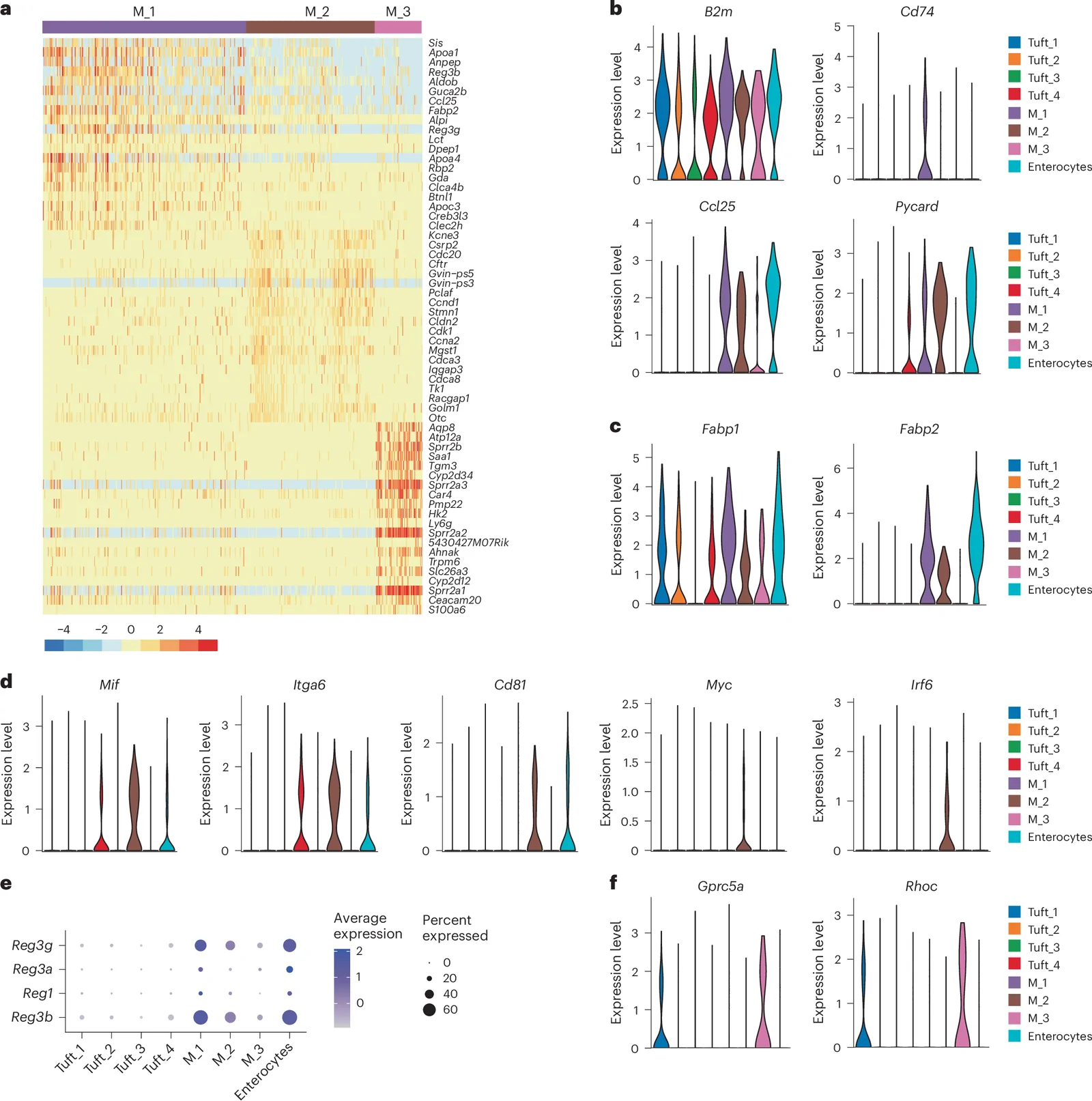
Distinct M cell clusters show gene signatures associated with antigen presentation, antimicrobial defense and immune cell interaction. **a**, Heatmap showing average expression of the top 20 DEGs in M_1, M_2 and M_3 cells identified by scRNA-seq of SPI-B-tdTomato^+^ IECs isolated from PPs, SIs and LIs of *Spib*^*tdTomato/+*^ mice at steady state or on day 3 after infection with *S.* *enterica* serovar Typhimurium SL1344 or *C.* *rodentium*. **b**, Violin plots showing expression of *B2m*, *Cd74*, *Ccl25* and *Pycard* in Tuft_1–Tuft_4, M_1–M_3 cells and enterocytes as in **a**. **c**, Violin plots showing expression *of Fabp1* and *Fabp2* in Tuft_1–Tuft_4, M_1–M_3 cells and enterocytes as in **a**. **d**, Violin plots showing expression of *Mlf, Itga6, Cd81, Myc* and *Irf6* in Tuft_1–Tuft_4, M_1–M_3 cells and enterocytes as in **a**. **e**, Dot plot showing expression of *Reg3g*, *Reg3a*, *Reg1* and *Reg3b* in Tuft_1–Tuft_4, M_1–M_3 cells and enterocytes as in **a**. Dot size indicates the percentage of cells expressing each gene; color indicates average expression. **f**, Violin plots of *Gprc5a* and *Rhoc* expression in Tuft_1–Tuft_4, M_1–M_3 cells and enterocytes as in **a**. **a**–**f** show data from one experiment (*n* = 5 mice for naive, 3 mice for *S*. Typhimurium SL1344 and 3 mice for *C.* *rodentium*).

M cells were enriched for genes associated with immune interaction (*Ccl25*), mucosal defense (*Muc3* and *Gvin2*) and epithelial metabolic functions (*Rbp2*) (Fig. 1c), as well as lysosomal and antigen-processing components^23^ (*Pycard*, *Ifi30* and *Grn*), barrier repair (*Hmgb2* and *Sprr2*) and antimicrobial responses (*Pglyrp1*, *Zg16* and *Dmbt1*) (Fig. 1d,e and Supplementary Table 5). The M cell heterogeneity revealed distinct M cell transcription programs in M_1, M_2 and M_3 populations (Fig. 2a). M_1 cells were enriched for genes involved with antigen presentation (*B2m* and *Cd74*), chemokine signaling^24^ (*Ccl25*), antimicrobial responses (*Pycard*) (Fig. 2b)^25,26^ and lipid metabolism (*Fabp1* and *Fabp2*) (Fig. 2c)^26^, suggesting a combined antigen handling and epithelial phenotype. M_2 cells shared many features with M_1, but showed higher expression of genes associated with adhesion, migration, antigen presentation^27,28^ and proliferation (*Mif*, *Itga6*, *Cd81*, *Myc* and *Irf6*)^29^ (Fig. 2d). Both M_1 and M_2 subsets expressed antimicrobial effector molecules (*Reg1/3a/b/g*) (Fig. 2e). M_3 cells, the smallest subset (~12% of M cells) were characterized by elevated levels of G-protein-coupled receptors (*Gpr137b* and *Gprc5a*) and tissue repair factors (*Areg* and *Hbegf*)^30,31^ (Fig. 2f and Extended Data Fig. 4d), indicating a potential role in epithelial repair. Collectively, SPI-B identified transcriptionally distinct M cell and tuft cell populations and revealed functionally specialized M cell subsets associated with antigen presentation, epithelial defense and tissue repair.

### Tissue and infection shape M cell transcriptional states

**Fig. 3:**
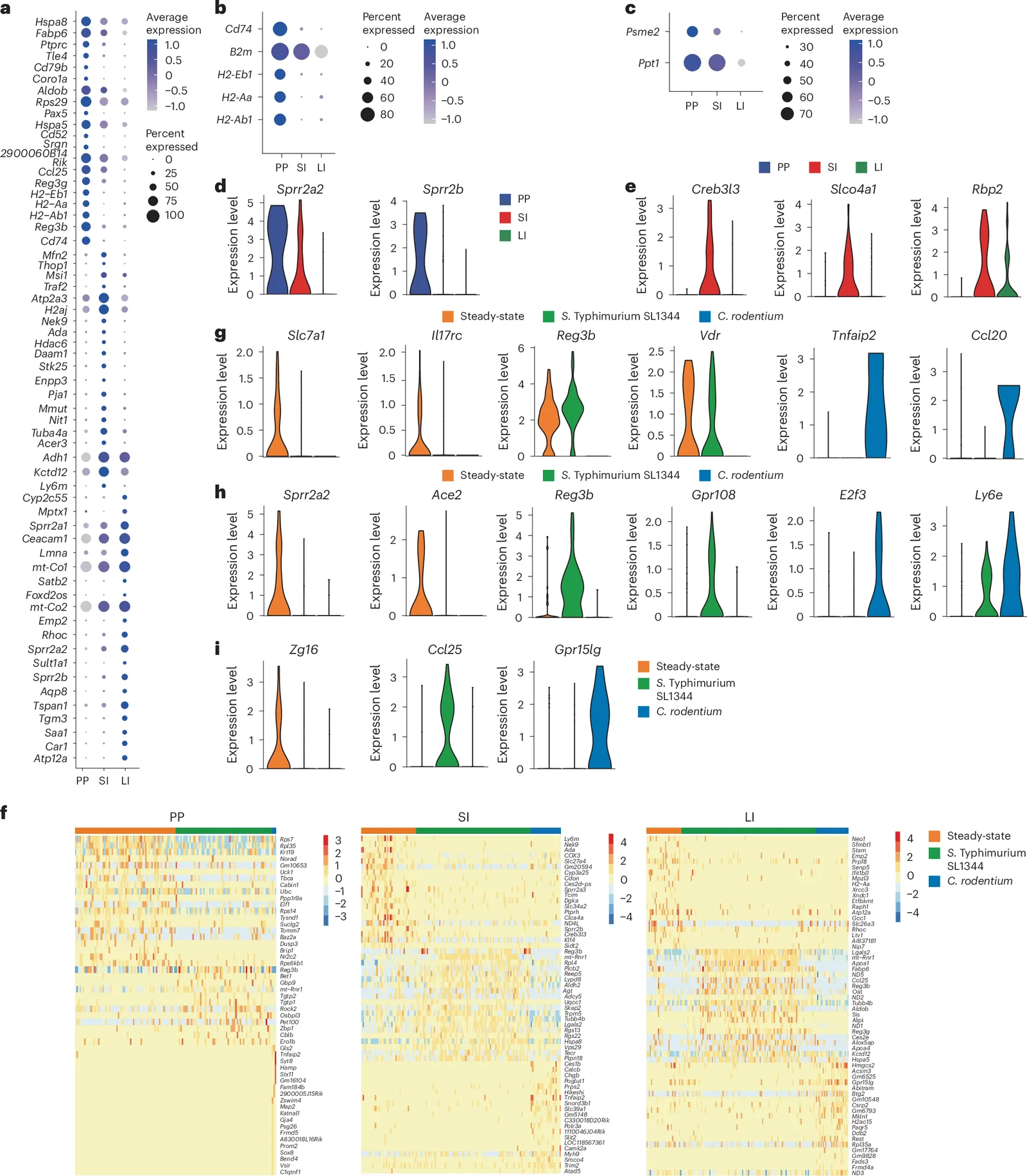
M cells show tissue-specific and pathogen-associated gene expression programs across intestinal regions. **a**, Dot plot showing top 20 DEGs in M cells isolated from PPs, SI and LI of steady-state *Spib*^*tdTomato/+*^ mice. Dot size represents the percentage of cells expressing each gene and color indicates the average expression of each gene. **b**, Dot plot showing expression of MHC class II complex components (*B2m*, *H2-Eb1*, *H2-Aa* and *H2-Ab1*) and *Cd74* genes in M cells isolated from PPs, SI and LI as in **a**. **c**, Dot plot showing of expression of *Psme2* and *Ppt1* in M cells from PP, SI and LI as in **b**. **d**,**e**, Violin plots showing expression of *Sprr2a2* and *Sprr2b* (**d**) or *Creb3l3*, *Slco4a1* and *Rbp2* (**e**) in M cells from PP, SI and LI as in **b**. **f**, Heatmaps showing the average expression of the top 20 DEGs in M cells isolated from PPs, SIs and LIs of *Spib*^*tdTomato/+*^ mice at steady state or on day 3 after *S.* *enterica* serovar Typhimurium SL1344 or *C.* *rodentium* infection. **g**–**i**, Violin plots showing expression of *Slc7a1*, *Il17rc*, *Reg3b*, *Vdr*, *Tnfaip2* and *Ccl20* in the PP (**g**), *Sprr2a2*, *Ace2*, *Reg3b*, *Gpr108*, *E2f3* and *Ly6e* in SI (**h**) and *Zg16*, *Ccl25* and *Gpr15lg* in the LI (**i**) M cell from *Spib*^*tdTomato/+*^ mice at steady state or on day 3 after *S.* *enterica* serovar Typhimurium SL1344 or *C.* *rodentium* infection as in **f**. **a**–**e** show data from one experiment (*n* = 5 mice). **f**–**i** show data from one experiment (*n* = 5 mice for naive, 3 mice for *S*. Typhimurium SL1344 and 3 mice for *C.* *rodentium*).

**Extended Data Fig. 5:**
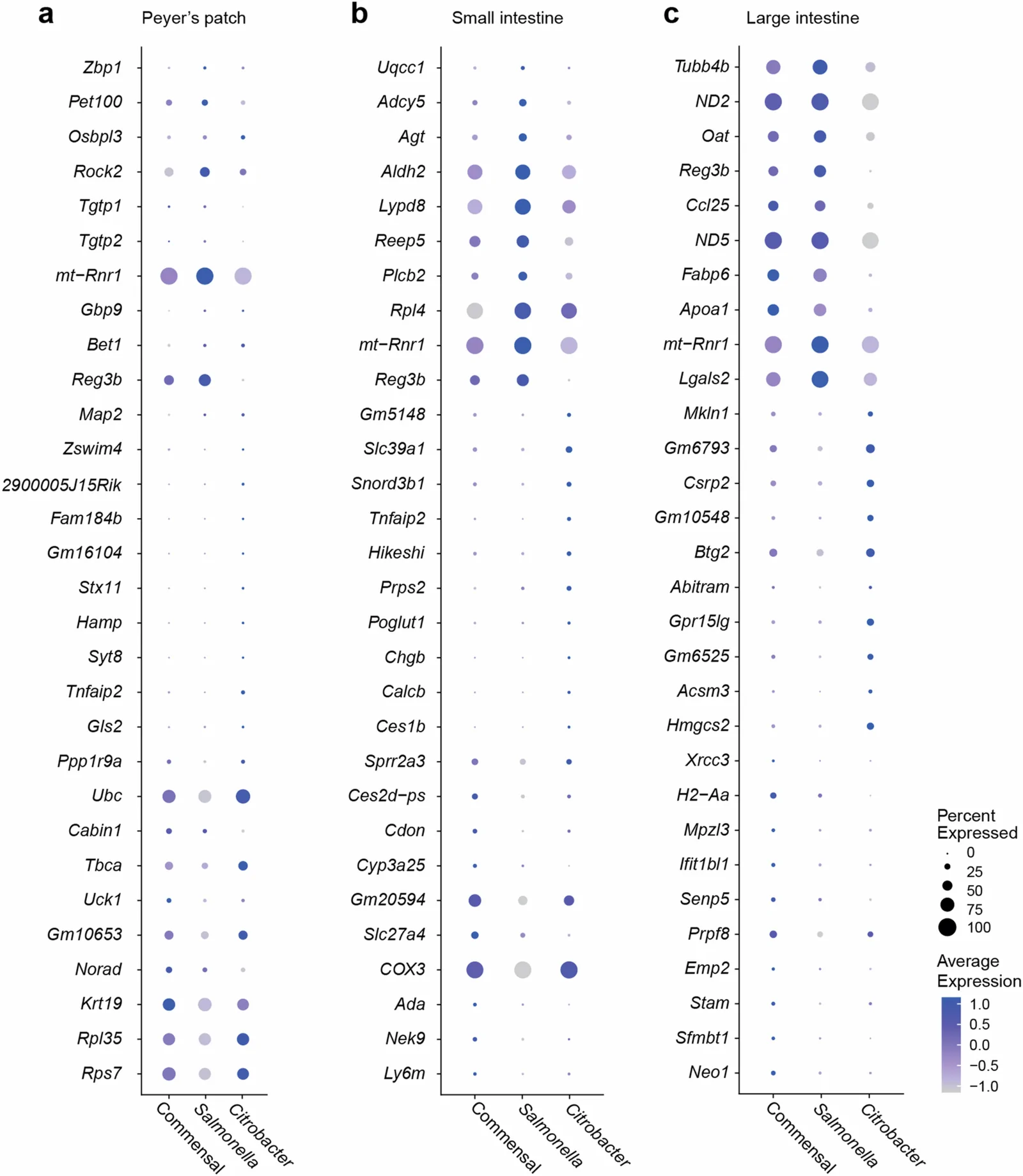
Infection-associated transcriptional programs in M cells. **a-c**, Dot plots showing expression of the top 10 genes in M cells from Peyer’s patches (**a**), small intestine (**b**) and large intestine (**c**) isolated from naïve (commensal) *Spib*^*tdTomato/+*^ mice or 3 days after infection with *Salmonella enterica* serovar Typhimurium SL1344 or *Citrobacter rodentium*. Dot size show the percentage of cells expressing each gene and color indicates average expression across conditions.

We next examined how these subsets programs varied across intestinal tissues and during enteric infection. At steady state, SPI-B-tdTomato^+^ Peyer’s patch M cells were enriched for genes associated with antigen processing and presentation, including MHC class I (*B2m*) and II complex components (*H2-Aa*, -*Ab1*, *-Eb1* and *Cd74*) (Fig. 3a,b), but comparatively weaker antigen presentation signatures were expressed by large intestine M cells (Fig. 3a). Peyer’s patch M cells also expressed regulators of antigen handling such as *Psme2* and *Ppt1* (Fig. 3c, Supplementary Fig. 2 and Supplementary Table 6) and antimicrobial and epithelial defense genes (*Reg3g*, *Sprr2a2*, *Sprr2b* and *Zg16*) (Fig. 3a,d)^32^. In contrast, villous M cells from the small intestine preferentially expressed metabolic (*Traf2*, *Nek9*, *Daam1*, *Ada* and *Rbp2*) and epithelial renewal pathways, including genes involved in lipid handling and solute transport (*Creb3l3* and *Slco4a1*), suggesting additional functions linked to nutrient sensing and epithelial homeostasis (Fig. 3e and Extended Data Fig. 5). Large intestine M cells were enriched for genes linked to epithelial integrity (*Emp2* and *Satb2*) and cell–cell interactions (*Emp2*, *Satb2*, *Ceacam1* and *Tspan1*) (Fig. 3a). These observations indicated that M cell functions were regionally specialized along the intestinal tract.

On day 3 after *S*. Typhimurium infection, Peyer’s patches M cells upregulated antimicrobial and stress-associated genes (*Nisch* and *Reg3b*) and cytoskeletal pathways, and concurrently downregulated genes associated with commensal interactions (*Ub*c, *Krt19* and *Il17rc*) when compared to Peyer’s M cells from steady-state mice (Fig. 3f and Extended Data Fig. 5a). M cells from the small and large intestine of *S*. Typhimurium-infected mice also upregulated genes linked to lipid metabolism (*Fabp6* and *Apoa1*), stress responses (*Lgals2*) and antigen presentation compared to Peyer’s patches M cells of *S*. Typhimurium-infected mice (Fig. 3f and Extended Data Fig. 5b,c), consistent with the ability of *Salmonella* to manipulate M cell biology during infection^10^. In contrast, infection with *Citrobacter* preferentially induced genes associated with inflammatory and epithelial repair programs (*Tnfaip2*, *Ccl20*, *Mkln1*, *Csrp2*, *E2f3* and *Gpr15lg*) and chemokines associated with ILC3 recruitment, but showed comparatively weaker antimicrobial responses (for example, *Reg3b*), in Peyer’s patch M cells (Fig. 3g and Extended Data Fig. 5a). Both *Citrobacter* and *Salmonella* infection induced decreased expression of MHC-II molecules (*H2-Aa*) in large intestine M cells compared to steady-state mice (Extended Data Fig. 5c). Expression of *Gpr108*, a negative regulator of Toll-like receptor signaling, was higher in small intestine M cells from *S*. Typhimurium-infected mice compared to naive mice or *Citrobacter*-infected mice (Fig. 3h and Extended Data Fig. 5b), suggesting enhanced restraint of TLR-driven inflammatory responses during *S*. Typhimurium infection. Expression of *Ccl25* was increased in large intestine M cells from *S*. Typhimurium-infected mice compared to naive mice or *Citrobacter*-infected mice (Fig. 3i), suggesting gene expression changes may alter immune cell recruitment, localization and/or interactions in response to infection^24^. Unlike the regional specialization observed at steady state, these infection-induced programs were broadly conserved across intestinal sites, indicating that pathogen-derived signals could override aspects of tissue-specific M cell identity (Fig. 3a–i and Extended Data Fig. 5a–c). Thus, M cell transcriptional responses were shaped by anatomical location at steady state and were remodeled in a pathogen-specific manner during enteric infection.

### M cells establish a niche for Peyer’s patch ILC3s

**Extended Data Fig. 6:**
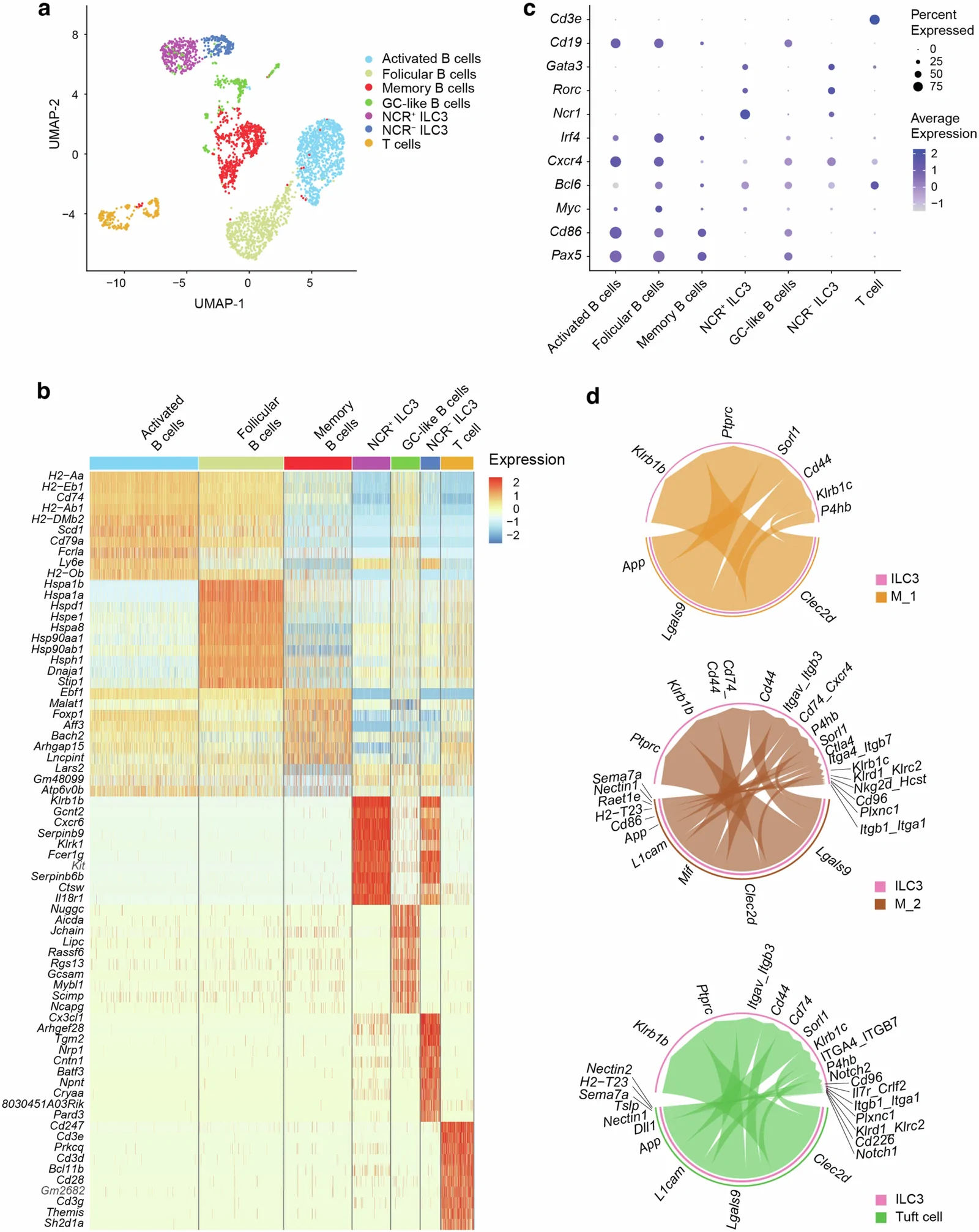
Predicted interactions between M cell subsets and ILC3s in murine Peyer’s patches. **a**, UMAP analysis of scRNA-seq data from Peyer’s patch immune cells showing major B cell, T cell and ILC3 subsets from naïve *Spib*^*tdTomato/+*^*Rorc(γt)*^*gfp/+*^ mice. **b**, Heatmap of top differentially expressed genes across Peyer’s patch immune cells indetified in **a**. **c**, Dot plot showing expression of canonical immune cell subset markers in each cluster. Dot size indicates the percentage of cells expressing each gene, and color scale indicates average expression. **d**, CellChat analysis showing predicted ligand–receptor interactions between M_1, M_2 subsets and tuft cells and their ligand-pair patterns in murine ILC3 cells. Only interactions with a *p* value < 0.05, as determined by permutation testing, are considered significant and shown in these diagrams.

**Extended Data Fig. 7:**
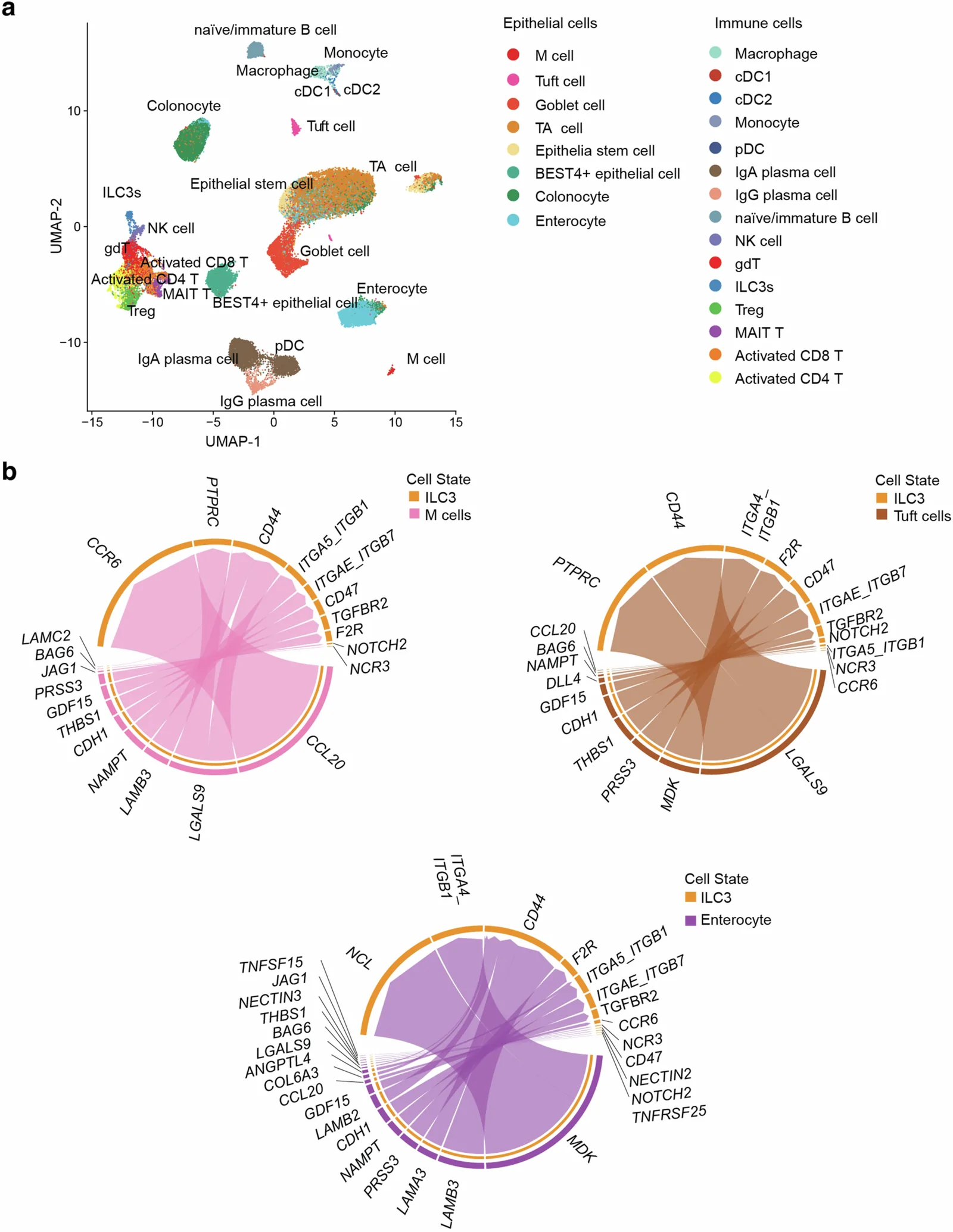
Predicted cell–cell communication between human intestinal epithelial populations and ILC3s. **a**, UMAP showing scRNA-seq profile of human intestinal epithelial cells and immune cells as described in Elmentaite *et al*. (2021)^1^. **b**, CellChat chord diagrams showing predicted interactions between the indicated human intestinal epithelial populations and ILC3s. The upper thinner bar represents the target cells that receive signals from the corresponding cells shown by the lower bar. The size of the inner arrow is proportional to the signal strength received by the target cells. Only interactions with a *p* value < 0.05, as determined by permutation testing are shown.

To examine ILC3–M cell interactions and distinguish M cells from other epithelial cells, RORγt-GFP^+^ ILC3s, CD45^+^RORγt-GFP^−^ immune cells and SPI-B-tdTomato^+^ epithelial cells purified from the Peyer’s patch of *Spib*^*tdTomato/+*^*Rorc*^*gfp/+*^ mice, which express tdTomato in epithelial cells and GFP in ILCs, were subjected to scRNA-seq to identify the differential gene expression in epithelial and lamina propria immune cells (Extended Data Fig. 6a–c). To investigate potential cell–cell communication between M_1, M_2 subsets or tuft cells and ILC3s, we used CellChat analysis^33^ to determine the probability of receptor–ligand interactions based on cell mRNA expression levels. This analysis indicated that different interactions occurred between M_1–ILC3s and M_2–ILC3s, and these were distinct from tuft cell–ILC3 interactions (Extended Data Fig. 6d). Predicted receptor–ligand interactions between M_1 and ILC3s were consistent with inhibitory or nonactivating roles, including *Lgals9*–*Ptprc/Cd44*, a pairing associated with suppression of immune cell activation and *L1cam*–*Itgav/Itgb3*, and adhesion interaction involved in positioning cells within tissues (Extended Data Fig. 6d). Reanalysis of a publicly available human gut epithelial–immune scRNA-seq dataset^34^ predicted M cell–ILC3 interactions were distinct from those involving tuft cells, or enterocytes and ILC3s, although several receptor–ligand signaling pathways were shared across epithelial populations (Extended Data Fig. 7a,b). CellChat analysis predicted that M cells signal to ILC3s primarily through M cell-expressed *CCL20*, a chemokine that recruits CCR6^+^ immune cells, acting via CCR6 on ILC3s, and through M cell-expressed *LAMC2*, an extracellular matrix component involved in cell adhesion and tissue organization, interacting with integrin receptors on ILC3s (Extended Data Fig. 7b). Tuft cells were predicted to signal through tuft cell-expressed *DLL4*, a Notch ligand involved in regulating cell differentiation and immune cell function, and required for ILC3 differentiation^35^ (Extended Data Fig. 7b). Enterocytes were predicted to signal through enterocyte-expressed *TNFSF15*, an inflammatory cytokine that regulates lymphocyte activation and survival, signaling through *TNFRSF25* on ILC3s (Extended Data Fig. 7b). These analyses indicated that M cells established a specialized epithelial niche that selectively supported ILC3 localization within Peyer’s patches.

### Epithelial SPI-B maintains intestinal ILC3 function

**Fig. 4:**
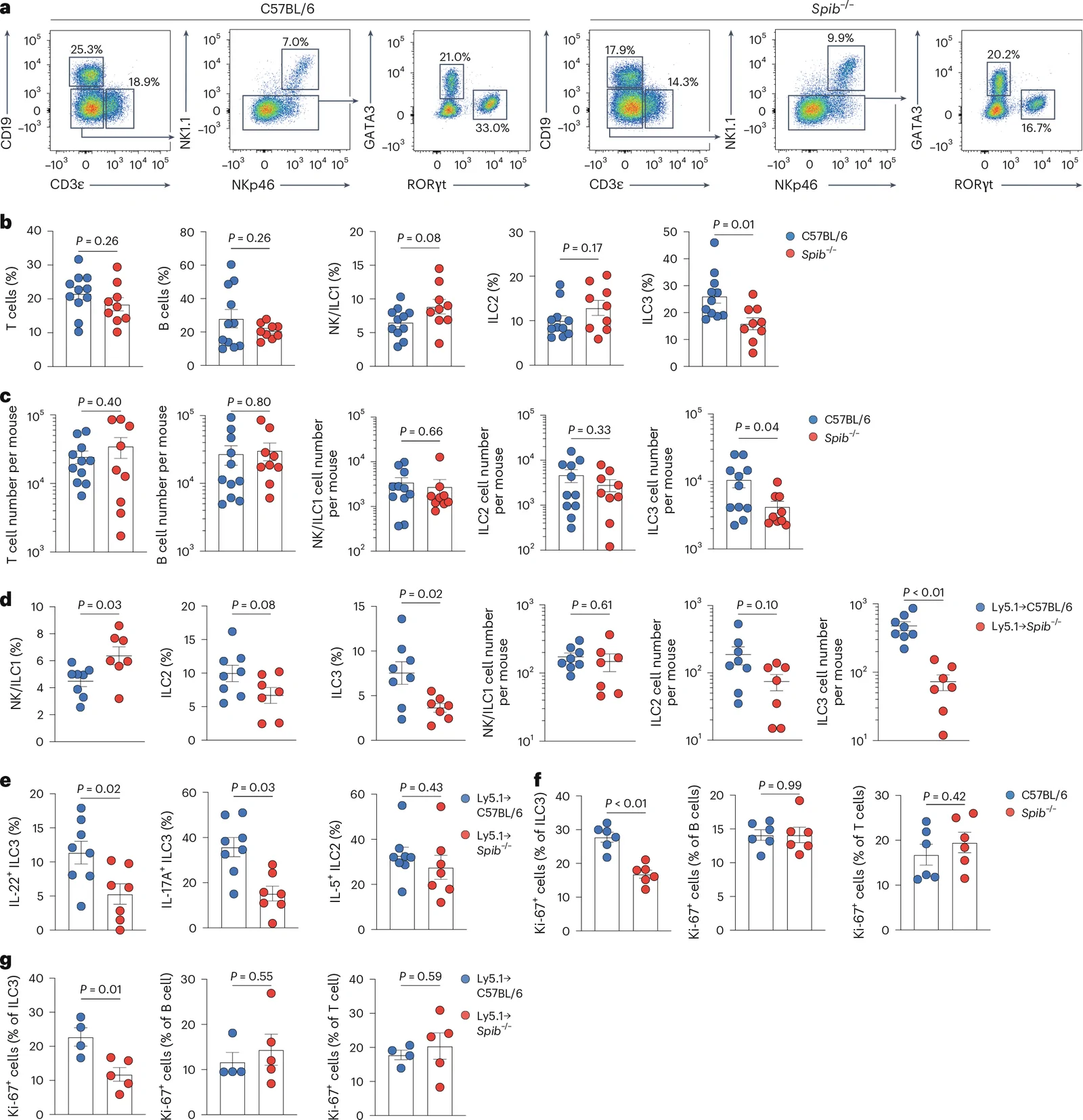
SPI-B deficiency alters innate lymphoid cell composition and proliferation. **a**, Representative dot plots showing the frequency of CD19^+^ B cells, CD3ε^+^ T cells, CD19^*−*^CD3ε^*−*^NK1.1^+^NKp46^+^ NK/ILC1s, CD19^*−*^CD3ε^*−*^NK1.1^*−*^ RORγt^*−*^ GATA3^+^ ILC2s, CD19^*−*^CD3ε^*−*^NK1.1^*−*^ GATA3^*−*^RORγt^+^ ILC3s in the SI of naive *Spib*^−/−^ and C57BL/6 mice. **b**,**c**, Frequency (**b**) and total number (**c**) of T cells, B cells, NK/ILC1s, ILC2s and ILC3s in the SI of *Spib*^*−/−*^ and C57BL/6 mice as in **a** (*n* = 9 or 11 mice per genotype). **d**, Frequency and total number of CD45.1^+^CD19^*−*^CD3ε^*−*^NK1.1^+^NKp46^+^ NK/ILC1, CD45.1^+^CD19^*−*^CD3ε^*−*^NK1.1^*−*^GATA3^+^RORγt^*−*^ ILC2s and CD45.1^+^CD19^*−*^CD3ε^*−*^NK1.1^*−*^GATA3^*−*^RORγt^+^ ILC3 isolated from the SI of C57BL/6 (Ly5.1→C57BL/6) or *Spib*^−/−^ (Ly5.1→*Spib*^−/−^) mice at week 8 post-reconstitution with Ly5.1 BM cells following lethal irradiation (*n* = 7 or 8 mice per genotype pooled from two independent experiments). **e**, Frequency of IL-17A^+^ ILC3s, IL-22^+^ ILC3s and IL-5^+^ ILC2s isolated from the SI of Ly5.1→C57BL/6 or *Spib*^−/−^ Ly5.1→*Spib*^−/−^ chimeric mice as in **d** following 4 h of culture with phorbol 12-myristate 13-acetate+ionomycin (*n* = 7 or 8 mice per genotype pooled from two independent experiments). **f**, Frequency of Ki*-*67^*+*^ ILC3, B and T cells isolated from the PP of C57BL/6 and *Spib*^*−*/*−*^ mice. Data pooled from two independent experiments (*n* = 5 or 6 mice per genotype). **g**, Frequency of Ki*-*67^*+*^ ILC3, B cells and T cells isolated from the PP of Ly5.1→C57BL/6 or *Spib*^−/−^ Ly5.1→*Spib*^−/−^ chimeric mice as in **d**. Data pooled from two independent experiments (*n* = 4 or 5 mice per genotype). **b**–**g** show data as mean ± s.e.m. Statistical analysis was performed using an unpaired two-tailed Student’s *t*-test. Source Data

**Extended Data Fig. 8:**
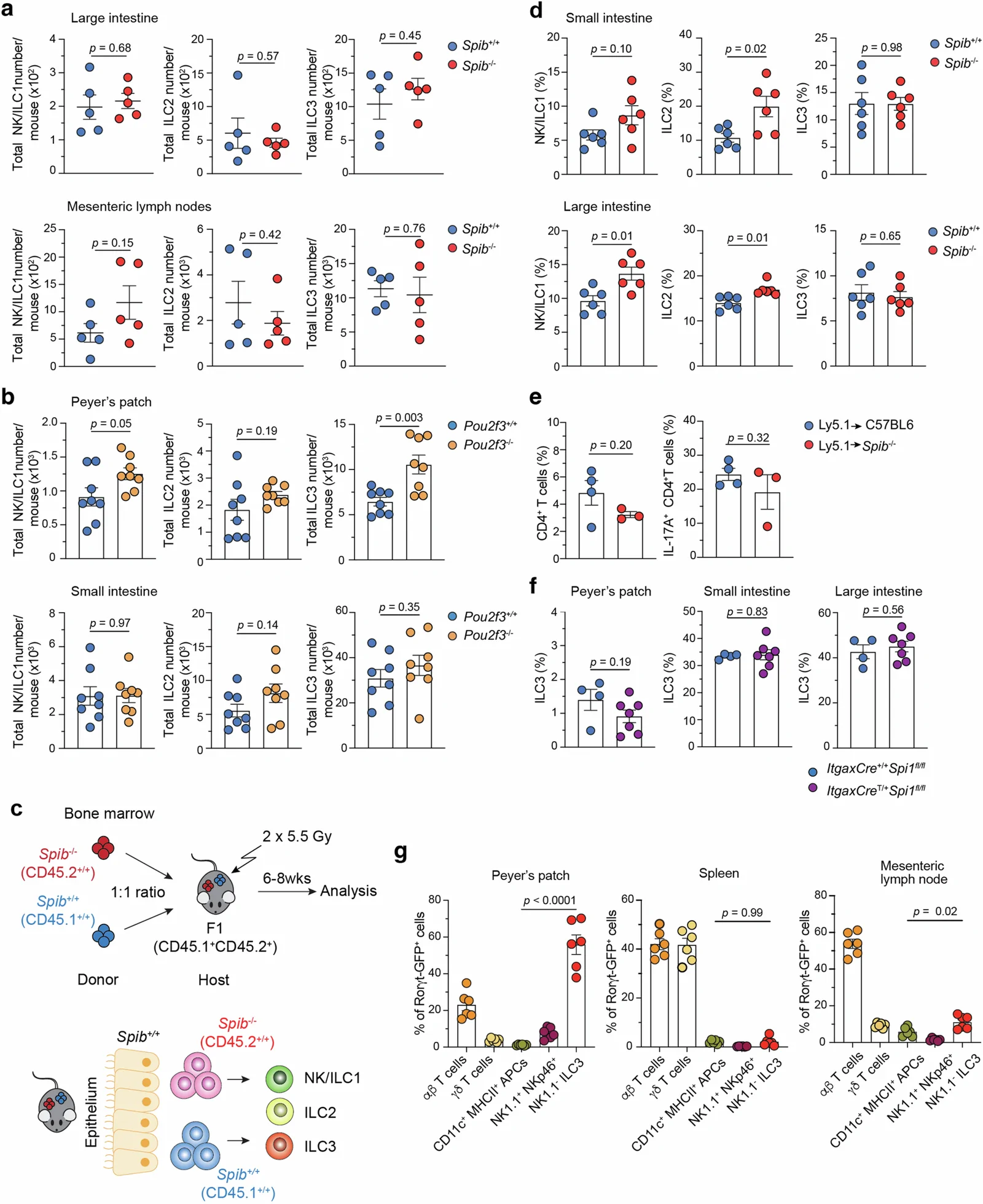
Small intestinal CD4+ T cells and ILCs in large intestine, mesenteric lymph nodes and lung are not disrupted by loss of non-hemopoietic SPI-B. **a**, Total number of NK/ILC1 (CD45.1^+^CD19^−^CD3ε^*−*^NK1.1^+^NKp46^+^), ILC2 (CD45.1^+^CD19^−^CD3ε^*−*^NK1.1^*−*^GATA3^+^Rorγt^*−*^) and ILC3 (CD45.1^+^CD19^−^CD3ε^*−*^NK1.1^*−*^GATA3^−^Rorγt^+^) cells isolated from the large intestine and mesenteric lymph nodes (mLN) of C57BL/6 and *Spib*^*−/−*^ mice. Data pooled from two independent experiment shown as mean ± s.e.m. (*n* = 5 mice/genotype). **b**, Total number of NK/ILC1 (CD45.1^+^CD19^−^CD3ε^*−*^NK1.1^+^NKp46^+^), ILC2 (CD45.1^+^CD19^−^CD3ε^*−*^NK1.1^*−*^GATA3^+^Rorγt^*−*^) and ILC3 (CD45.1^+^CD19^−^CD3ε^*−*^NK1.1^*−*^GATA3^−^Rorγt^+^) cells isolated from the Peyer’s patch and small intestine of naive *Pou2f3*^*+/+*^ and *Pou2f3*^*−/−*^ mice. Data pooled from two independent experiments shown as mean ± s.e.m. (*n* = 8 mice/genotype). **c**, Schematic showing generation of mixed bone marrow chimeric mice. Lethally irradiated F1 (CD45.1^+^CD45.2^+^) host mice received 1:1 ratio mixed bone marrow isolated from *Spib*^*−/−*^ (CD45.2^+/+^) and Ly5.1 (CD45.1^+/+^) mice (top). Illustration showing experimental approach to distinguish SPI-B^+^ or SPI-B^*−*^ cell reconstitution in intestinal lamina propria (bottom). **d**, Frequency of *Spib*^*+/+*^ (CD45.1^+/+^) and *Spib*^*−/−*^ (CD45.2^+/+^) ILC subsets within their respective donor compartments (CD45.1^+^ or CD45.2^+^) isolated from the small or large intestine of mixed bone marrow chimeric mice as in **c**. Data show pooled from two experiments shown as mean ± s.e.m. (*n* = 6 mice/genotype). **e**, Frequency of CD4^+^ T cells (live CD3ε^+^TCRβ^+^CD4^+^) and Th17 (live CD3ε^+^TCRβ^+^CD4^+^ RORγt^+^) cells isolated from the small intestine of chimeric C57BL/6 or *Spib*^*−/−*^ host mice at 8 weeks after reconstitution with Ly5.1 BM cells following lethal irradiation. Data from one experiment shown as mean ± s.e.m. (*n* = 3 or 4 mice/genotype). **f**, Frequency of ILC3 (CD45^+^CD19^−^CD3ε^*−*^NK1.1^*−*^GATA3^−^Rorγt^+^) in the Peyer’s patch, small and large intestine of naive *Itgax*^*CreT/+*^*Spi1*^*fl/fl*^ mice. Data pooled from two independent experiments shown as mean ± s.e.m (*n* = 4 or 7 mice/genotype). **g**, Flow cytometric quantification showing the frequency of RORγt-eGFP^+^ cells in the Peyer’s patch, spleen and mesenteric lymph node of naïve *Rorc*^*gfp/+*^ mice. αβ T cells (live CD45.2^+^CD19^−^CD90.2^+^CD3ε^+^TCRβ^+^), γδ T cells (live CD45.2^+^CD19^−^CD90.2^+^CD3ε^+^TCRβ^*−*^), APCs (live CD45.2^+^CD19^−^CD90.2^*−*^CD11c^+^MHCII^+^) and ILC3s (live CD45.2^+^CD19^−^CD90.2^+^CD3ε^*−*^TCRβ^*−*^NK1.1^*−*^IL-18Rα^+^CXCR6^+^). Data pooled from two experiments shown as mean ± s.e.m. (*n* = 3 mice/experiment). Statistical significance was determined using an unpaired two-tailed Student’s *t*-test (**a**,**b**,**d**-**g**). Source Data

To determine whether epithelial SPI-B influenced innate immune composition, we quantitated ILC populations in the small intestine of *Spib*^−/−^ and wild-type mice. The number of small intestine CD45^+^ immune cells, including CD19^+^ B cells and CD3ε^+^ T cells were unchanged in *Spib*^−/−^ and wild-type mice (Fig. 4a–c and Supplementary Fig. 3). In contrast, the number of total CD45^+^CD19^*−*^CD3ε^*−*^ ILCs was significantly reduced in the small intestine of *Spib*^−/−^ mice, with this reduction attributable mainly to loss of RORγt^+^ group 3 ILCs (ILC3s), whereas NK1.1^+^NKp46^+^ ILC1 and GATA3^+^ ILC2 were not significantly altered (Fig. 4b,c). ILC populations in the large intestine or mesenteric lymph nodes were not significantly altered in *Spib*^−/−^ compared to wild-type mice (Extended Data Fig. 8a). To exclude the possibility that this phenotype reflected the loss of tuft cells, we analyzed *Pou2f3*^−/−^ mice, which lack tuft cells^36^. The number of ILC3s in the small intestine in *Pou2f3*^−/−^ mice were comparable to those in wild-type controls (Extended Data Fig. 8b) indicating that the loss of ILC3 in *Spib*^−/−^ mice was not attributable to tuft cell deficiency.

To determine whether the loss of ILC3s was driven by loss of SPI-B in the epithelial or hematopoietic compartment, we generated BM chimeras in which lethally irradiated *Spib*^−/−^ or C57BL/6 host mice were reconstituted with BM cells from congenically labeled Ly5.1 mice (Ly5.1→*Spib*^−/−^ and Ly5.1→C57BL/6, respectively) (Fig. 4d). In Ly5.1→*Spib*^−/−^ mice, the frequencies and absolute numbers of ILC3s in the small intestine were significantly reduced compared to Ly5.1→C57BL/6 chimeras (Fig. 4d,e). In mixed BM chimeras, where CD45.1 CD45.2 wild-type mice were reconstituted with 1:1 ratio of CD45.2^+^ *Spib*^−/−^ and CD45.1^+^ wild-type BM cells, *Spib*^−/−^ and wild-type ILCs reconstituted intestinal tissues equivalently (Extended Data Fig. 8c,d), indicating that loss of ILC3 numbers in *Spib*^−/−^ mice was not cell-autonomous.

Next, we assessed whether loss of epithelial SPI-B affected ILC effector function. ILC3 isolated from the small intestine of Ly5.1→*Spib*^−/−^ chimeric mice stimulated with phorbol 12-myristate 13-acetate+ionomycin for 4 h exhibited a marked reduction in the production of IL-22 and a more modest reduction in IL-17A expression (Fig. 4e), whereas ILC2 or CD4^+^ T cells from Ly5.1→*Spib*^−/−^ chimeric mice produced comparable levels of IL-5 (Fig. 4e) or IL-17 (Extended Data Fig. 8e) respectively, compared to Ly5.1→C57BL/6 chimeras. Ki-67 staining revealed a significant decrease in proliferating ILC3 in the Peyer’s patches of naive *Spib*^−/−^ mice (Fig. 4f) and Ly5.1→*Spib*^−/−^ chimeric mice compared to Ly5.1→C57BL/6 chimeras (Fig. 4g), whereas proliferation of B cells and T cells was similar in Ly5.1→*Spib*^−/−^ and Ly5.1 →C57BL/6 chimeras (Fig. 4g). ILC1, ILC2 and ILC3 isolated from the large intestine and mesenteric lymph nodes of Ly5.1 →*Spib*^−/−^ chimeras had comparable proliferation with Ly5.1→C57BL/6 chimeras (Extended Data Fig. 8f). To determine whether the reduction in the number of ILC3s reflected altered DC-mediated signaling downstream of M cells, we analyzed *Itgax*^*cre/+*^*Spi1*^*fl/fl*^ mice, in which DCs are selectively depleted, whereas M cells remain intact. In these mice, the frequency and number of ILC3s in the small intestine and Peyer’s patches were comparable to those in *Itgax*^*+/+*^*Spi1*^*fl/fl*^ littermates (Extended Data Fig. 8f), indicating that the reduction in ILC3s in *Spib*^−/−^ mice was not secondary to loss of classical DC populations. To confirm that the RORγt^+^ cells we identified in our studies were ILC3s, rather than conventional CD3ε^+^TCRβ^+^ αβ T cells or CD3ε^+^TCRβ^*−*^ γδ T cells or the recently described MHCII^+^RORγt^+^ cells, we analyzed the Peyer’s patch, spleen and mesenteric lymph node of naive *Rorc*^*gfp/+*^ mice using the established RORγt^+^ ILC3 markers CXCR6 and IL-18R^37,38^. This showed that only ~1% of RORγt^+^ cells in Peyer’s patch were CD11c^+^, suggesting that they could be CD11c^+^MHCII^+^RORγt^+^ cells, whereas the majority of RORγt^+^ cells in Peyer’s patch were CD3ε^−^NK1.1^−^CXCR6^+^ IL-18Rα^+^ ILC3 (ref. ^39^; Extended Data Fig. 8g).

**Extended Data Fig. 9:**
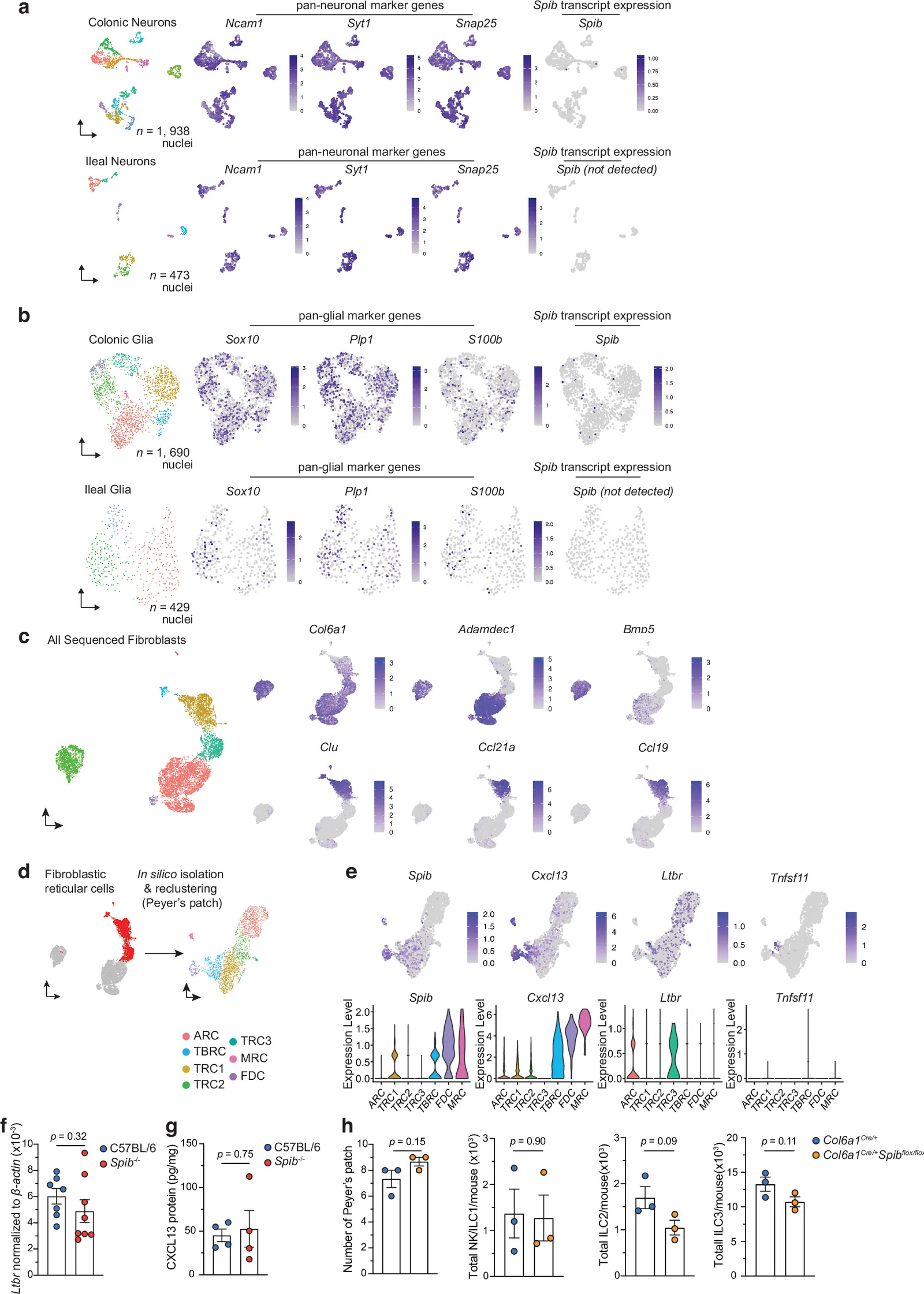
Spib expression is detected in Peyer’s patch stromal cells, but not in intestinal neurons or glia. **a**, UMAP of neuronal nuclei isolated from colon and ileum. Clusters correspond to transcriptionally distinct neuronal subsets as defined in the original dataset. Expression of pan-neuronal marker genes (*Ncam, Syt1* and *Snap25*) and *Spib* is shown across neuronal clusters. **b**, UMAP visualization of enteric glial nuclei isolated from colon and ileum. Expression of canonical glial marker genes (*Sox10, Plp1* and *S100b*) and *Spib* is shown across glial clusters. Color scales represent normalized expression values for each gene. **a**, **b**, Data were re-analyzed from the publicly available single-nucleus RNA-seq dataset reported in Drokhlyansky *et al*.^2^ **c**, UMAP of fibroblasts scRNA-seq public data from Prados *et al*.^3^ in which stromal cells (defined as CD31^−^CD45^−^PDPN^+^ and/or VCAM^+^ cells) were isolated from murine Peyer’s patches. Clustering analyses applied to all sorted fibroblasts demonstrated multiple fibroblast subsets characterized by differential expression of marker genes including *Adamdec1, Bmp5*, and *Clu*. Clustering analyses was applied to all sorted fibroblasts demonstrating multiple fibroblast subsets characterized by differential expression of marker genes including *Adamdec1, Bmp5, Clu*, *Ccl21a* and *Ccl19*. **d**, UMAP analysis showing *in silico* re-clustering of Peyer’s patch fibroblastic reticular cells from total purified fibroblasts as in **c**. Re-clustered fibroblastic reticular cell subsets were annotated as adventitial reticular cells (ARCs), T–B reticular cells (TBRCs), T cell reticular cells 1–3 (TRC1–3), follicular dendritic cells (FDCs) and marginal reticular cells (MRCs). **e**, Feature plots and violin plots showing expression of *Spib*, *Cxcl13*, *Ltbr* and *Tnfsf11* across Peyer’s patch fibroblastic reticular cell subsets shown in **d**. Color scales indicate normalized expression values for each gene. **f**, qPCR quantification of *Ltbr* gene expression in tissue lysates of Peyer’s patches isolated from C57BL/6 and *Spib*^*−/−*^ mice. Data pooled from two independent experiments (*n* = 7 or 8 mice/genotype). **g**, CXCL13 protein quantification in the tissue lysates of Peyer’s patches isolated from C57BL/6 and *Spib*^*−/−*^ mice. Data pooled from two independent experiments shows as mean ± s.e.m. (*n* = 4 mice/genotype). **h**, Number of Peyer’s patch (left) and ILC subsets (right panel) in the Peyer’s patches in the *Col6a1*^*Cre/+*^ and *Col6a1*^*Cre/+*^*Spib*^*flox/flox*^ mice. Data from one experiment shown as mean ± s.e.m. (*n* = 3 mice/genotype). Statistical significance was determined using an unpaired two-tailed Student’s *t-*test (**f**-**h**). Source Data

Analysis of publicly available neuronal, glial^40^ and Peyer’s patch stromal cell^41^ scRNA-seq datasets revealed minimal *Spib* expression outside the epithelial compartment (Extended Data Fig. 9a–e). Expression of *Ltbr* and *Tnfsf11* (encodes RANKL) was detected rarely in follicular DCs, and these transcripts were not coexpressed with *Spib*, while *Cxcl13* expression was restricted to *Cr2*^*+*^*Cttnbp2*^*+*^ follicular DCs and *Madcam1*^*+*^ marginal reticular cell populations (Extended Data Fig. 9e). Expression of *Ltbr* and *Cxcl13* in the Peyer’s patches was not significantly different in *Spib*^−/−^ and wild-type mice (Extended Data Fig. 9f,g). Consistent with this, deletion of SPI-B in stromal cells using *Col6a1*^*cre*^*Spib*^*fl/fl*^ mice did not show a loss of Peyer’s patch ILCs, including ILC3 (Extended Data Fig. 9h). Collectively, stromal and other non-epithelial cells showed minimal or no SPI-B-dependent expression of *Ltbr*, *Tnfsf11* and *Cxcl13*, and stromal-specific SPI-B deletion did not affect Peyer’s patch ILC3 numbers indicating SPI-B^+^ stromal cells do not play an important role in regulating ILC3 homeostasis.

### SPI-B^+^ epithelial cells organize Peyer’s patch ILC3s

**Fig. 5:**
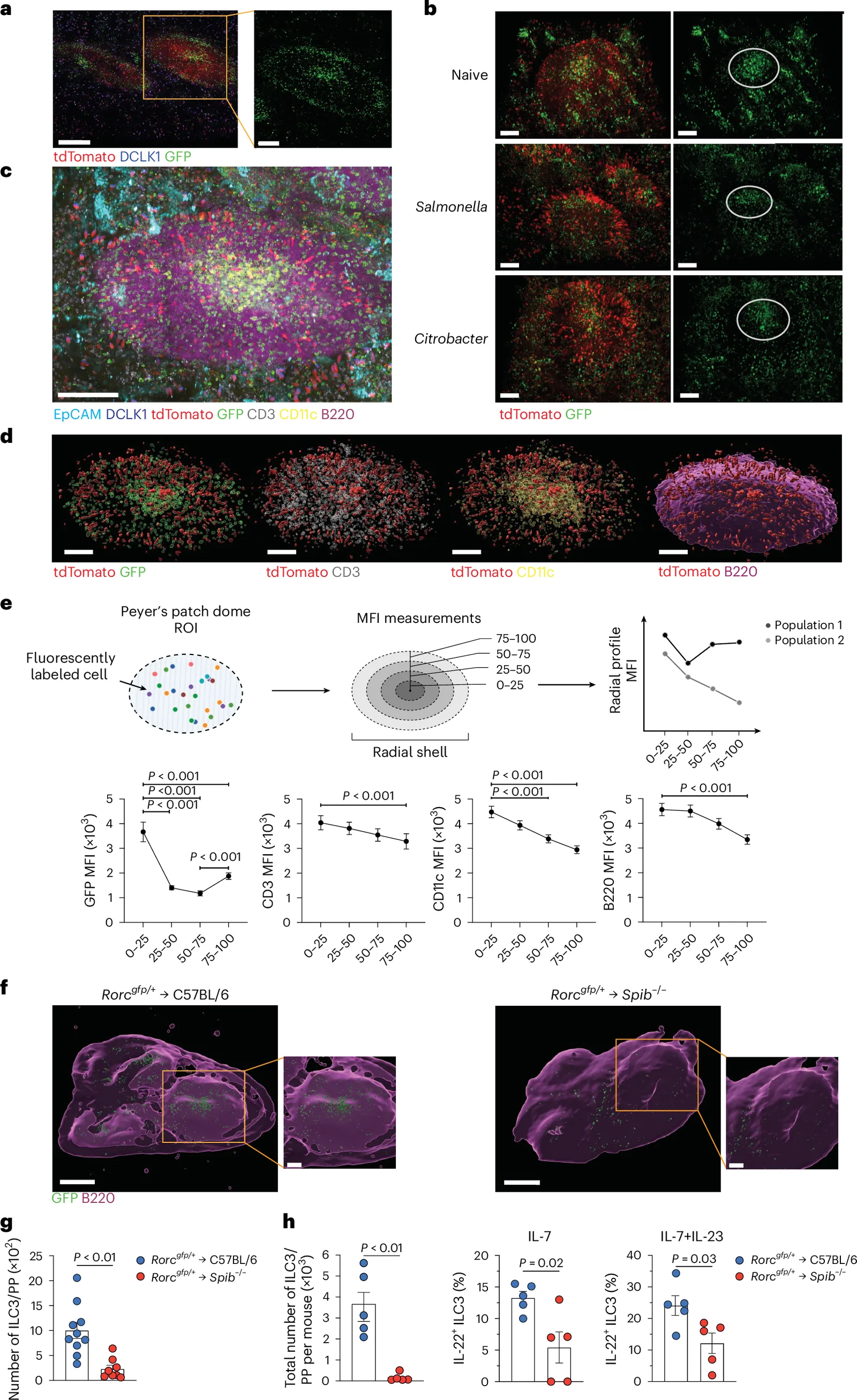
SPI-B-expressing epithelial cells regulate ILC3 localization in Peyer’s patches. **a**, Representative immunofluorescence of SPI-B-tdTomato, DCLK1 and RORγt-GFP staining in PPs isolated from naive *Spib*^*tdTomato/+*^*Rorc*^*gfp/+*^ mice. Scale bar, 300 μm (left) and 150 μm (right). *n* = 4 or 5 mice per condition from two independent experiments. **b**, Representative images of SPI-B-tdTomato staining in PPs from *Spib*^*tdTomato/+*^*Rorc*^*gfp/+*^ mice at steady state or day 3 after *S.* *enterica* or *C.* *rodentium* infection. Ovals indicate the central follicle regions. Scale bars, 100 μm. Representative of *n* = 4 or 5 mice per condition from two independent experiments. **c**, Representative immunofluorescence of EpCAM, DCLK1, SPI-B-tdTomato, RORγt-GFP, CD3, CD11c, B220 in PPs from naive *Spib*^*tdTomato/+*^*Rorc*^*gfp/+*^ mice. Scale bar, 50 μm. Representative of two independent experiments (*n* = 10 mice). **d**, Three-dimensional (3D) reconstruction of SPI-B-tdTomato^+^ epithelial cell association with RORγt-GFP^+^ ILC3s, CD11c^+^ DCs, CD3^+^ T cells and B220^+^ B cells in PPs from naive *Spib*^*tdTomato/+*^*Rorc*^*gfp/+*^ mice. Scale bars, 50 μm **e**, Schematic showing method for the quantification of mean fluorescence intensity (MFI) in the radial profile of PP based on four concentric radial bins (0–25%, 25–50%, 50–75% and 75–100%) across the dome of the PP (top) and quantification of MFI distribution for CD11c, RORγt, B220 and CD3 in the radial shell of PP from *Spib*^*tdTomato/+*^*Rorc*^*gfp/+*^ mice (bottom). Data are pooled from three experiments (*n* = 10 mice). ROI, region of interest. **f**, 3D reconstructions showing RORγt-GFP^+^ ILC3 and B220^+^ B cells in PPs isolated from *Rorc*^*gfp/+*^→C57BL/6 or *Rorc*^*gfp/+*^→*Spib*^−/−^ chimeric mice at week 8 after lethal irradiation and reconstitution. Scale bars, 300 μm. Images are representative of three independent experiments (*n* = 9 or 10 mice per genotype). **g**, Immunofluorescence-based quantification of the number of ILC3s per PP in *Rorc*^*gfp/+*^→C57BL/6 or *Rorc*^*gfp/+*^→*Spib*^−/−^ chimeric mice as in **f**. **h**, Flow cytometric quantification of total number of ILC3s in PP (left) and frequency of IL-22^+^ ILC3 following 4 h of culture with IL-7 (center) or IL-7 + IL-23 (right) in *Rorc*^*gfp/+*^→C57BL/6 or *Rorc*^*gfp/+*^→*Spib*^−/−^ chimeras as in **f**. Data are pooled from two experiments (*n* = 5 mice per group). **e**, **g**, and **h** show data as mean ± s.e.m. *P* values were derived by paired (**e**) or unpaired (**g**,**h**) two-tailed Student’s *t*-tests. Source Data

**Extended Data Fig. 10:**
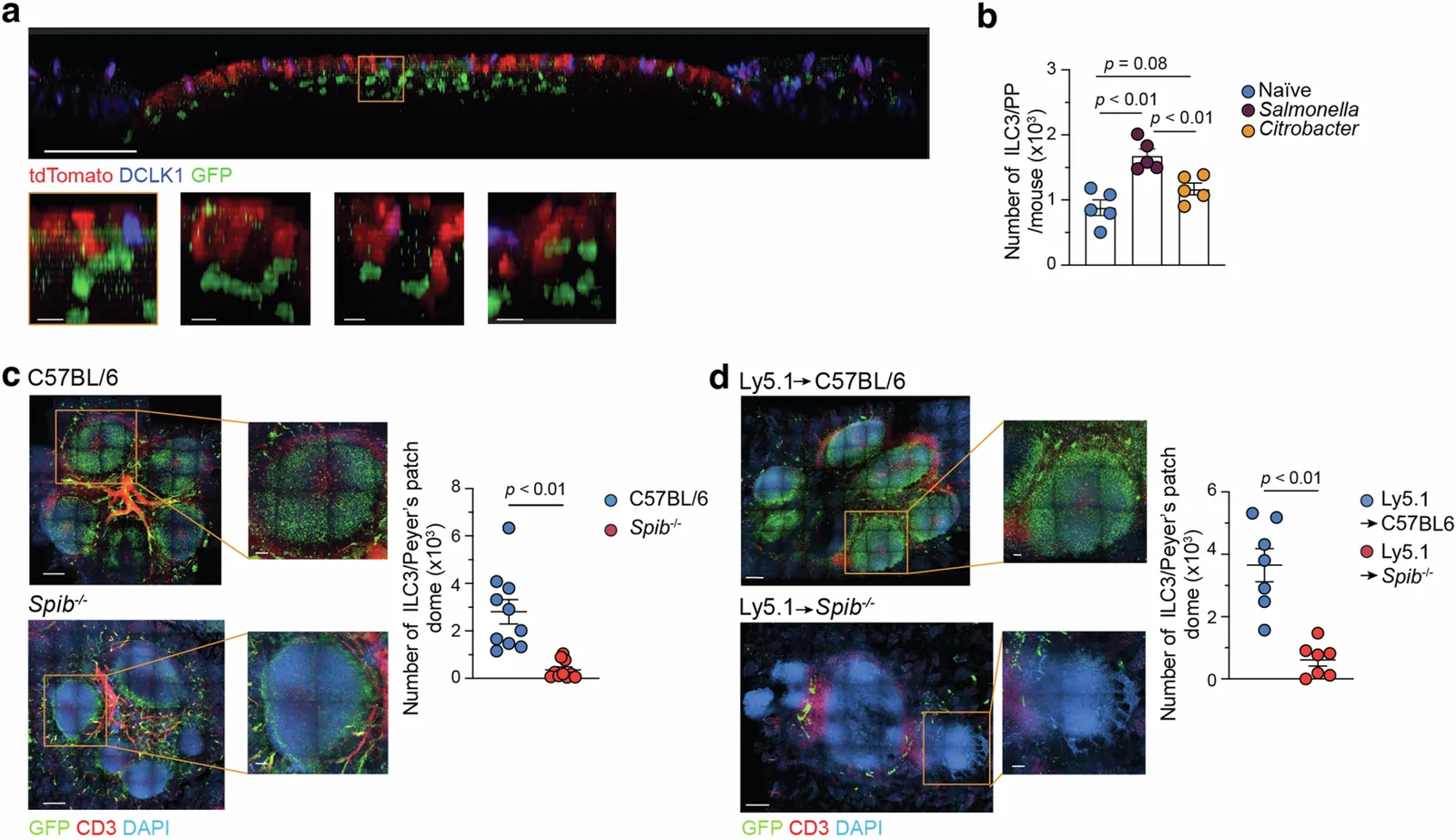
M cells and tuft cells are positioned within the Peyer’s patch epithelial dome. **a**, Immunofluorescence imaging of Peyer’s patch dome in naïve *Spib*^*tdTomato/+*^*Rorc*^*gfp/+*^ mice. Images representative of two independent experiment with similar results (*n* = 5 mice). Data show merged fluorescence signals for SPI-B-tdTomato (anti-tdTomato, red), RORγt-GFP (anti-GFP, green) and DCLK1 (blue). Scale bar, 100μm. Insets, representative magnified epithelial areas (bottom), scale bar 10μm. **b**, Immunofluorescence imaging-based quantification of number ILC3 in Peyer’s patch isolated from naïve *Spib*^*tdTomato/+*^*Rorc*^*gfp/+*^ mice or 3 days after infection with *Salmonella enterica* serovar Typhimurium SL1344 or *Citrobacter rodentium*. Data pooled from two independent experiments shown as mean ± s.e.m. (*n* = 5 mice/condition). CD3ε^+^ T cells were excluded from ILC3 quantification. Statistical significance was determined using an unpaired two-tailed Student’s *t*-test. c, Representative immunofluorescence images (left) and quantification of ILC3 number in Peyer’s patches of C57BL/6 and *Spib*^*−/−*^ mice 3 days after infection with *Salmonella enterica* serovar Typhimurium SL1344. Data pooled from two experiments shown as mean ± s.e.m. (*n* = 10 mice/genotype). Scale bars, 50 μm. **d**, Representative immunofluorescence images (left) and quantification of ILC3 number in Peyer’s patches isolated at 3 days after infection with *Salmonella enterica* serovar Typhimurium SL1344 of chimeric C57BL/6 and *Spib*^*−/−*^ mice that were lethally irradiated and reconstituted with Ly5.1 BM cells 8 weeks before infection. Data pooled from two experiments shown as mean ± s.e.m. (*n* = 7 mice/genotype). Scale bars, 50 μm. Statistical significance was determined using an unpaired two-tailed Student’s *t*-test (**c**,**d**). Source Data

To determine whether epithelial SPI-B regulated the spatial distribution of ILC3 within the Peyer’s patches, we performed confocal microscopy of cleared whole-mount small intestinal tissues from *Spib*^*tdTomato/+*^*Rorc*^*gfp/+*^ mice. DCLK1^+^ tuft cells and SPI-B-tdTomato^+^ DCLK1^−^ M cells were detected within the Peyer’s patch dome epithelium and along intestinal villi (Fig. 5a and Extended Data Fig. 10a). In naive *Spib*^*tdTomato/+*^*Rorc*^*gfp/+*^ mice, RORγt-eGFP^+^ ILC3 formed a discrete and reproducible accumulation beneath the Peyer’s patches dome epithelium, with a central cluster overlying the SPI-B-tdTomato^+^ B cell follicle surrounded by a peripheral ring within the subepithelial region, resembling a ‘fried egg’ (Fig. 5a and Supplementary Videos 1 and 2). This organization was maintained at day 3 post-infection with *Salmonella* and *Citrobacter* (Fig. 5b and Extended Data Fig. 10b). The number of Peyer’s patch ILC3s was significantly expanded at day 3 after *Salmonella* infection, along with a ~1.6-fold increase in Ki-67^+^ ILC3s compared to naive mice, indicating local proliferation of ILC3s, whereas ILC2 numbers remained unchanged and ILC1s slightly increased (Supplementary Fig. 4a–c). Imaging analysis showed that SPI-B-tdTomato^+^DCLK1^−^ M cells were closely associated with both CD11c^+^ DCs and RORγt-eGFP^+^ ILC3s (Fig. 5c,d). Radial shell analysis revealed progressive reduction in CD11c, B220 and CD3 expression from the follicle center toward the outer dome, whereas RORγt^+^ ILC3s exhibited a biphasic distribution, with enrichment in the inner and outer regions and relative depletion between these zones (Fig. 5e).

To determine whether ILC3 organization depended on epithelial SPI-B, lethally irradiated *Spib*^−/−^ or wild-type recipients were reconstituted with *Rorc*^*gfp/+*^ BM. RORγt-GFP^+^ ILC3s accumulated normally beneath the Peyer’s patch dome epithelium in *Rorc*^*gfp/+*^ →C57BL/6 mice but were largely absent in *Rorc*^*gfp/+*^→*Spib*^−/−^ chimeras, resulting in an ~tenfold reduction in follicle-associated ILC3s (Fig. 5f–h). Similar changes were observed in *Salmonella*-infected Ly5.1→*Spib*^−/−^ and Ly5.1→C57BL/6 chimeric mice (Extended Data Fig. 10c,d). Peyer’s patch ILC3s were transcriptionally distinct from steady-state small intestine ILC3 in C57BL/6 mice (Supplementary Fig. 5a) and exhibited greater constitutive production of IL-22 (Supplementary Fig. 5b). Following 4 h of ex vivo stimulation with IL-7 or IL-7 + IL-23, Peyer’s patch ILC3s isolated from *Rorc*^*gfp/+*^→*Spib*^−/−^ chimeras produced significantly less IL-22 than ILC3 from *Rorc*^*gfp/+*^→C57BL/6 controls (Fig. 5h). Thus, SPI-B^+^ epithelial cells supported both the positioning and the IL-22-producing capacity of Peyer’s patches ILC3s.

### CCR6 guides M cell-associated ILC3 localization

**Fig. 6:**
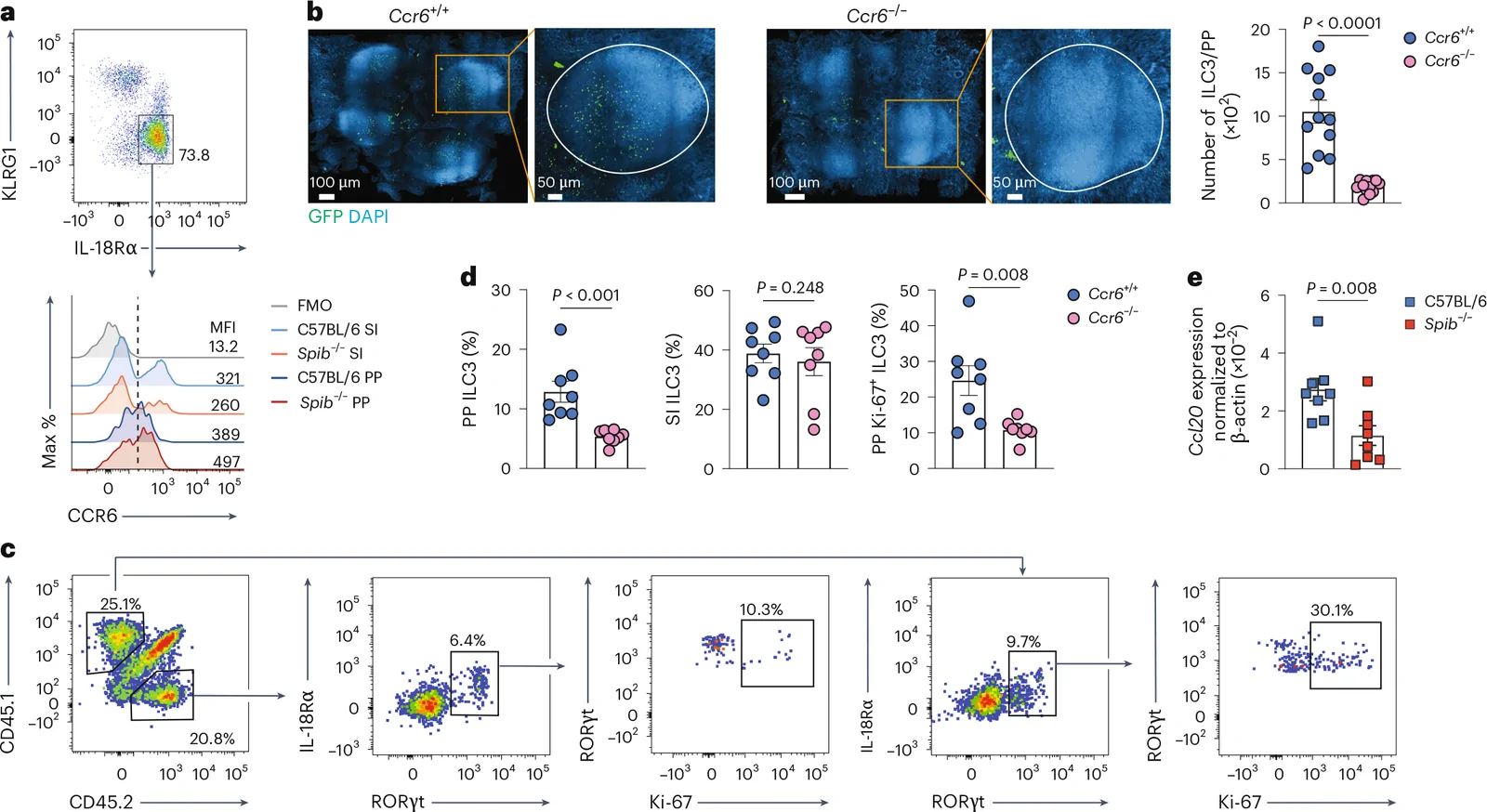
CCR6–CCL20 signaling is required for ILC3 positioning in the Peyer’s patch dome. **a**, Representative flow cytometry plot showing frequency of KLRG^*−*^IL-18Rα^+^ ILC3s among live CD19^−^CD3ε^−^NK1.1^−^ cells (top) and representative histogram showing expression of CCR6 in ILC3s from SI and PP of C57BL/6 or *Spib*^−/−^ mice (bottom). Data are representatives of two independent experiments (*n* = 4 mice per genotype). FMO, fluorescence minus one. **b**, Representative immunofluorescence images (left) and quantification (right) of RORγt^+^ ILC3 in PP from *Ccr6*^+/+^ and *Ccr6*^−/−^ mice. Data show individual PPs collected from four mice per genotype as mean ± s.e.m. **c**, Representative flow cytometry plots showing *Ccr6*^*+/+*^CD45.1^+^ or *Ccr6*^−/−^CD45.2^+^IL-18Rα^+^RORγt^+^ ILC3s and IL-18Rα^+^RORγt^+^ proliferating ILC3s in PP of CD45.1 CD45.2 C57BL/6 host mice reconstituted with a 1:1 mix of congenically labeled CD45.1^+^*Ccr6*^*+/+*^ and CD45.2^+^*Ccr6*^−/−^ BM cells at week 8 post lethal irradiation and reconstitution. Cells were pre-gated on live lin^−^CD90.2^+^IL-7Rα^+^ cells. **d**, Frequency of *Ccr6*^*+/+*^ or *Ccr6*^−/−^ ILC3s in PP (left) and SI (middle), and frequency of Ki-67^+^ ILC3s in PPs (right) in mixed chimeras as in **c**. Data are pooled from independent experiments (*n* = 8 mice). **e**, qPCR quantification of *Ccl20* in PP isolated from C57BL/6 or *Spib*^−/−^ mice at steady state. Data are pooled from two independent experiments (*n* = 8 mice per genotype). Data are shown as mean ± s.e.m. (**b**,**d**,**e**). Statistical analysis was performed using a paired (**d**) or an unpaired (**b**,**e**) two-tailed Student’s *t*-test. Source Data

To define the mechanisms controlling ILC3 positioning within the Peyer’s patch dome, we examined CCR6 expression on Peyer’s patch and small intestine ILC3s isolated from C57BL/6 wild-type and *Spib*^−/−^ mice. Flow cytometry analysis showed robust expression of CCR6 on Peyer’s patch and small intestine KLRG1^*−*^IL-18Rα^+^ ILC3s, with similar CCR6 gMFI in ILC3s from *Spib*^−/−^ and wild-type mice (Fig. 6a). Confocal imaging of Peyer’s patches from wild-type and *Ccr6*^−/−^ mice showed that RORγt^+^ ILC3s were enriched within the subepithelial dome of wild-type mice but were reduced by ~5.6-fold in *Ccr6*^−/−^ mice and showed reduced density within the subepithelial dome compared to wild-type mice (Fig. 6b). This indicated that CCR6 was required for the localization and/or retention of ILC3s within the Peyer’s patch.

To determine whether CCR6 acted intrinsically in ILC3s, we reconstituted lethally irradiated CD45.1 CD45.2 wild-type recipient mice with a 1:1 mixture of whole BM from CD45.1 Ly5.1 and CD45.2 *Ccr6*^−/−^ mice. Within the Peyer’s patch, donor wild-type CD45.1^+^ ILC3s were preferentially enriched relative to *Ccr6*^−/−^CD45.2^+^ ILC3s at 8 weeks after BM transfer (Fig. 6c,d), while no significant differences were observed in the small intestine (Fig. 6d). Donor *Ccr6*^−/−^ ILC3s displayed reduced frequency of Ki-67^+^ cells compared to *Ccr6*^+/+^ ILC3s (Fig. 6d), suggesting impaired expansion within organized lymphoid tissues and identifying a cell-intrinsic role for CCR6 in promoting ILC3 accumulation within Peyer’s patches. As CCR6-dependent localization requires the ligand CCL20, we examined whether chemokine production was altered in *Spib*^−/−^ epithelial cells. Expression of *Ccl20* mRNA was reduced in the Peyer’s patch epithelial cells in *Spib*^−/−^ mice compared to C57BL/6 mice (Fig. 6e), indicating that epithelial cell SPI-B regulated expression of *Ccl20* in vivo. Thus, epithelial SPI-B was an upstream regulator of the CCL20-CCR6 axis that controlled the accumulation of ILC3s within the Peyer’s patch dome.

### RANK–RANKL signaling controls epithelial ILC3 abundance

**Fig. 7:**
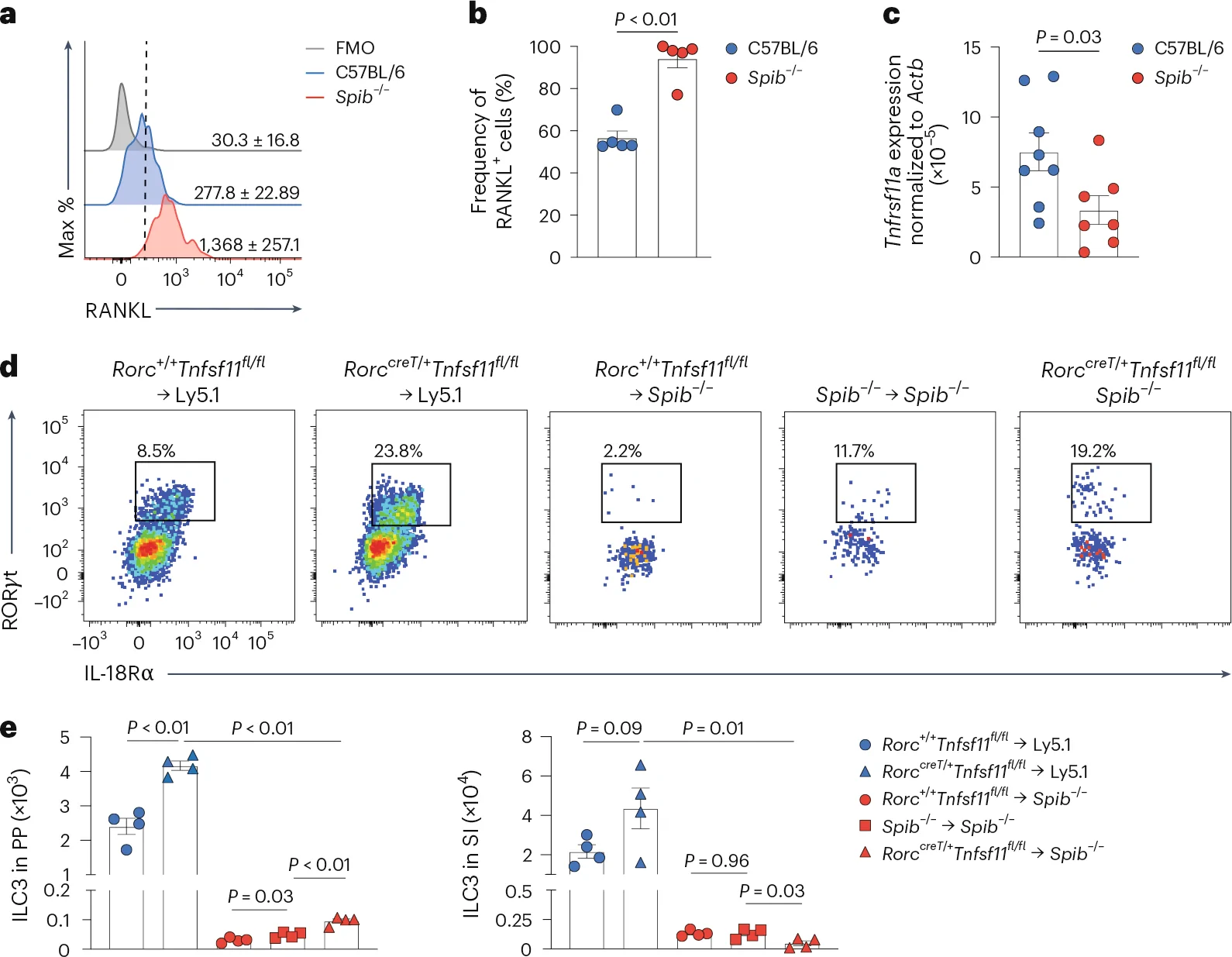
Loss of SPI-B drives RANKL upregulation but impairs ILC3 homeostasis in the small intestine. **a**, Representative flow cytometry histograms showing expression of RANKL in PP live CD45^+^CD19^*−*^CD3ε^*−*^TCRβ^*−*^IL-18Rα^+^CXCR6^+^ ILC3s isolated from C57BL/6 and *Spib*^*−*/*−*^ mice at steady state. Representative plots from one of two independent experiments are shown, MFI quantification shown as mean ± s.e.m. (*n* = 5 mice per genotype). **b**, Frequency of RANKL^+^ ILC3s in PP from C57BL/6 and *Spib*^*−*/*−*^ mice at steady state as in **a**. Data of two independent experiments with similar results as mean ± s.e.m. (*n* = 5 mice per genotype). **c**, qPCR quantification of *Tnfrsf11a* in PP epithelium of steady-state C57BL/6 (*n* = 8) and *Spib*^*−*/*−*^ mice (*n* = 7). Data pooled from two independent experiments shown as mean ± s.e.m. **d**, Representative flow cytometry plots showing donor-derived live lin^−^CD45.2^+^RORγt^+^IL-18Rα^+^ ILC3s in PP from CD45.2 *Rorc*^*+/+*^*Tnfsf11*^*fl/fl*^→CD45.1 WT, CD45.2 *Rorc*^*creT/+*^*Tnfsf11*^*fl/fl*^→CD45.1 WT, CD45.2 *Rorc*^*+/+*^*Tnfsf11*^*fl/fl*^→CD45.1 *Spib*^−/−^, CD45.2 *Spib*^−/−^→CD45.1 *Spib*^−/−^ or CD45.2 *Rorc*^*creT/+*^*Tnfsf11*^*fl/f l*^→CD45.1 *Spib*^−/−^ chimeric mice at week 8 after lethal irradiation and reconstitution. **e**, Total number of ILC3s recovered from PP and SI of BM chimera as in **d**. **d** and **e** show data from one of two independent experiments with similar results as mean ± s.e.m. (*n* = 4 mice per group). Statistical significance was determined using an unpaired two-tailed Student’s *t*-test (**b**,**c**,**e**). Source Data

SPI-B is induced downstream of RANK–RANKL signaling during M cell differentiation^42^. Flow cytometric analysis indicated that the frequency of RANKL^+^ ILC3s and RANKL expression in ILC3 were increased by approximately 2.1-fold and 4.9-fold, respectively, in Peyer’s patches from *Spib*^−/−^ mice compared to wild-type mice (Fig. 7a,b). qPCR showed that RANK, which was largely restricted to Peyer’s patch epithelial cells, was significantly reduced in *Spib*^−/−^ compared to wild-type Peyer’s patch (Fig. 7c). To determine whether RANK–RANKL signaling between Peyer’s patch ILC3s and epithelial cells influenced ILC3 homeostasis, we generated BM chimeras in which congenically labeled BM cells from wild-type, *Spib*^−/−^ or *Rorc*^*creT/+*^*Tnfsf11*^*fl/fl*^ mice, in which *Tnfsf11* encoding RANKL is conditionally deleted in *Rorc-*expressing cells, including ILC3s, were transferred into wild-type or *Spib*^−/−^ mice to generate *Rorc*^*cre+/+*^*Tnfsf11*^*fl/fl*^→Ly5.1, *Rorc*^*creT/+*^*Tnfsf11*^*fl/fl*^→Ly5.1, *Rorc*^*cre+/+*^*Tnfsf11*^*fl/fl*^→Ly5.1 *Spib*^−/−^, Ly5.2 *Spib*^−/−^→Ly5.1 *Spib*^−/−^ and *Rorc*^*creT/+*^*Tnfsf11*^*fl/fl*^→Ly5.1 *Spib*^−/−^ chimeras. Deletion of *Tnfsf11* within *Rorc*-expressing hematopoietic cells in *Rorc*^*creT/+*^*Tnfsf11*^*fl/fl*^→Ly5.1 chimeras increased Peyer’s patch ILC3 numbers compared to the *Rorc*^*cre+/+*^*Tnfsf11*^*fl/fl*^→Ly5.1 chimeras (Fig. 7d), consistent with the reported negative regulatory role of RANKL on ILC3 proliferation^43^, whereas loss of SPI-B in epithelial cells in *Rorc*^*cre+/+*^*Tnfsf11*^*fl/fl*^→Ly5.1 *Spib*^−/−^, Ly5.2 *Spib*^−/−^→Ly5.1 *Spib*^−/−^ and *Rorc*^*creT/+*^*Tnfsf11*^*fl/fl*^→Ly5.1 *Spib*^−/−^ chimeras resulted in loss of ILC3s in Peyer’s patch and small intestine compared to *Rorc*^*cre+/+*^*Tnfsf11*^*fl/fl*^→Ly5.1 chimeras (Fig. 7e). ILC3 cell numbers were not recovered by the additional loss of RANKL in ILC3s in the *Rorc*^*creT/+*^*Tnfsf11*^*fl/fl*^→Ly5.1 *Spib*^−/−^ chimeras compared to *Rorc*^*creT/+*^*Tnfsf11*^*fl/fl*^→Ly5.1 chimeras in the Peyer’s patch and small intestine (Fig. 7e), indicating that although RANKL restrained the expansion of ILC3s, SPI-B-dependent pathways in epithelial cells were dominant in establishing the tissue niche that supported ILC3 accumulation. Collectively, these data demonstrated that epithelial SPI-B-dependent chemokine signaling and RANK–RANKL pathways acted as complementary mechanisms that coordinated the positioning and abundance of ILC3 in Peyer’s patches.

## Discussion

Here we showed that Peyer’s patch M cells contributed to the organization of a local epithelial niche that supported ILC3 homeostasis and function within the intestinal mucosa. Analysis of SPI-B^+^ epithelial cells revealed marked heterogeneity within the M cell lineage and identified distinct epithelial programs associated with tissue location and microbial challenge. Epithelial SPI-B was required beyond M cell differentiation, maintaining ILC3 abundance, positioning and cytokine production in Peyer’s patches. Together, these findings support a role for M cells as organizers of epithelial–immune interactions that coordinate innate and adaptive components of mucosal immunity.

The transcriptional diversity observed within the M cell compartment suggests that M cells are functionally specialized according to their anatomical location and local environmental cues. Although M cells have largely been considered a uniform antigen-sampling population^5,44^, the distinct gene expression programs identified here are consistent with broader roles in integrating microbial, metabolic and immune signals. Whether these transcriptional states represent stable subsets or dynamic responses to environmental stimuli remains unclear. The association between M cell signatures and microbial community changes further suggests M cells influence host–microbiota interactions beyond antigen uptake^45^.

Our findings expand the functional repertoire of M cells beyond antigen transcytosis and initiation of adaptive immune responses^44^. While previous studies linked M cells to dendritic cell maturation and T cell priming^46,47^, our work identified a role for M cells in maintaining local innate immune organization through selective regulation of ILC3s. Together with analogous tuft cell–ILC2 and Paneth cell–ILC3 interactions^4,48^, the M cell–ILC3 circuit defines an epithelial–immune signaling pathway that contributes to gut homeostasis. Although reduced proliferation likely contributed to the loss of ILC3s, the relative contribution of impaired recruitment, retention or survival remains unresolved. The persistence of the M cell–ILC3 architecture during infection suggests that this niche represents a stable structural feature of Peyer’s patches capable of supporting inflammatory responses without extensive tissue reorganization.

Mechanistically, our data support a model in which epithelial SPI-B influenced ILC3 localization through CCR6-dependent positioning cues. Reduced CCL20 expression in the absence of epithelial SPI-B, together with the requirement for CCR6 in Peyer’s patch ILC3 accumulation, is consistent with established roles for this pathway in lymphoid tissue organization and ILC3 positioning^24^; however, the incomplete loss of ILC3s suggests that additional adhesion molecules, extracellular matrix components or chemokine pathways contribute to niche maintenance. The relationship between M cells and ILC3s intersected with RANK–RANKL signaling, a pathway classically associated with M cell differentiation^5,42^. Recent studies have shown that RANKL can directly regulate ILC3 function^43^, and our findings suggest that epithelial and hematopoietic components of this pathway operate within a shared regulatory network in which epithelial niche-derived signals are dominant determinants of ILC3 accumulation within Peyer’s patches.

ILC3-derived IL-22 is a central regulator of mucosal barrier defense, epithelial repair and host-microbial interactions^49^. The association between M cell-dependent niche organization and IL-22 production therefore links epithelial architecture with cytokine-mediated barrier protection. This pathway seemed distinct from previously described dendritic cell-dependent mechanisms of IL-22 regulation^50^, suggesting that multiple spatially segregated circuits cooperate to control IL-22 availability within Peyer’s patches.

Collectively, these findings support a model in which M cells organize regional immune niches that integrate environmental sensing with immune cell positioning and effector function. The distinct transcriptional programs observed across intestinal locations further suggest that epithelial–immune interactions are regionally compartmentalized and adapted to tissue requirements, enabling specialized responses to microbial, dietary and inflammatory cues while maintaining tissue homeostasis. More broadly, these findings highlight how specialized epithelial populations shape immune organization through spatially restricted interactions and provide a framework for understanding how disruption of epithelial–immune communication may contribute to intestinal disease.

## Methods

### Mice

C57BL/6 (B6, Ly5.2^+/+^), B6.SJL-*Ptprc*^*a*^*Pep3*^*b*^*/*BoyJ (Ly5.1^+/+^), *Prdm1*^*gfp/+*^ (ref. ^14^), *Spib*^−/−11^, *Rorc(γt)*^*gfp/+*^ (*Rorc*^*gfp/+*^)^51^, *Itga*^*cre*^*Spi1*^*fl/fl*^ (ref. ^52^), *Tnfsf11*^*fl/fl*^ (B6.129-*Tnfsf11*^*tm1.1Caob*^/J)^53^, *Rorc(γt)*^*creT/+*^ (*Rorc*^*creT/+*^)^43^, *Ccr6*^−/−^ (B6.129P2-*Ccr6*^*tm1Dgen*^/J)^54^ and *Pou2f3*^−/−^ (C57BL/6J-*Pou2f3em1Cbwi*/J)^36^ mice have been described previously. *Spib*^*tdTomato*^ (*Spib*^*T2A−dTomato−IRES−ERT2cre*^), *Spib*^*em1Gtbz*^ (ref. ^13^) and *Prdm1*^*gfp*^ mice were generated on a C57BL/6 background. *Spib*^−/−^ and *Rorc*^*gfp/+*^ mice were backcrossed to C57BL/6 mice for more than ten generations and all mouse strains were maintained on a C57BL/6 background. *Spib*^*tdTomato/+*^*Rorc*^*gfp/+*^ were generated by intercrossing the respective strains. Mice were bred and housed in specific=pathogen-free conditions in ventilated cages (22 °C and 50–70% relative humidity) under a 12-h light–dark cycle with ad libitum access to food and water. Both male and female mice were analyzed at 6–16 weeks of age unless otherwise stated. Mice of different genotypes were not co-housed. All animal experiments were conducted according to approval by the Animal Ethics Committees of The University of Queensland or the Walter and Eliza Hall Institute of Medical Research and procedures were carried out in accordance with the regulatory standards of the National Health and Medical Research Council (NHMRC) and the Australian Code for the Responsible Conduct of Research.

### Generation of *Spib*^*T2A−dTomato−IRES−ERT2cre*^ mouse line

The *Spib*^*T2A−dTomato−IRES−ERT2cre*^ mouse line was generated by inserting the T2A-reporter-IRES-ERT2Cre construct after the last coding exon in each strain by homologous recombination. sgRNA sequences *Spib*: AGCGGAGTGCTCAGACATGC. Reporter mice were generated by inserting cut sites 5′ of the gene of interest followed by T2A linked to a fluorescent reporter and a CreERT2. *Frt* sites flanked the Cre construct to allow removal of the ERT2Cre while leaving the reporter construct intact. Validation of each strain was performed by PCR using the following primers and conditions:

tdTomato sequence: forward, 5′-AGTTCTTGCTGGGGTTGCTC-3′;

reverse, 5′-GCACCTTGAAGCGCATGAAC-3′; amplicon size 381 bp.

ERT2 sequence: forward, 5′-TGGAGATCTTCGACATGCTG-3′;

reverse, 5′-CACGTTCTTGCACTTCATGC-3′; amplicon size 335 bp.

The conditions used for PCR reactions were 95 °C for 2 min (95 °C × 30 s, 60 °C × 30 s and 72 °C × 30 s) ×35 cycles, 72 °C × 5 min.

### Generation of bone marrow chimeric mice

Recipient mice (6–10 weeks old) were lethally irradiated with two doses of 5.5 Gy (3 h apart). BM cells were collected from donor mice by flushing the femoral and tibial bones with 3 × 1 ml sterile DPBS to create a single cell suspension. Red blood cells were removed by osmotic lysis using Red Cell Removal Buffer for 5 min, then washed twice with 10 ml DPBS before counting live cells using Trypan blue exclusion. Cells (2.5 × 10^6^) resuspended in 200 μl sterile DPBS were then adoptively transplanted into the irradiated recipients. Chimeric mice were allowed 6–10 weeks to fully reconstitute their hematopoietic system with donor BM cells.

### Bacterial infections

*S.* *enterica* serovar Typhimurium SL1344 was grown statically, and the attenuated strain *S.* Typhimurium BRD509 was grown under gentle rotation, at 37 °C in Luria-Bertani (LB) broth containing 100 μg ml^−1^ streptomycin overnight, then diluted in Dulbecco’s phosphate-buffered saline (DPBS; Gibco). Bacteria were washed and pelleted (3,700*g*,10 min, 4 °C) and resuspended in 10% w/v NaHCO_3_ in DPBS. Then, 2 × 10^7^ colony-forming units (c.f.u.) SL1344 or 2 × 10^9^ c.f.u. BRD509 resuspended in 100 μl of 10% NaHCO_3_ were administered to mice intragastrically.

*C.* *rodentium* was grown in LB broth containing 10 mM nalidixic acid (37 °C, gentle rotation, overnight) then centrifuged and the pellet resuspended in DPBS. Food was withdrawn for 8 h before infection, and 6–8-week-old mice (male and female) were inoculated by oral gavage with 1.5 × 10^9^ c.f.u. *C.* *rodentium*.

### Isolation of intestinal intraepithelial and lamina propria lymphocytes

The small intestine and colon were collected from each mouse. Peyer’s patches were removed from the small intestine and pooled from each individual animal. Intestinal tissues were cleaned of fecal material and cut into 0.5-cm^2^ pieces. IECs were isolated from the intestine by incubating tissues with gentle shaking to dissociate epithelial cells (40 min, 37 °C, Ca^2+^ and Mg^2+^-free Hank’s medium containing 5 mM ethylenediaminetetraacetic acid (EDTA) and 2% FCS). The solution containing epithelial cells was then centrifuged at (5 min, 4 °C, 550*g*) and the pellet was washed twice. The remaining tissues were then incubated in 1–2 mg ml^−1^ collagenase type IV (Worthington), 1 μg ml^−1^ DNase I (Roche), 0.4 U ml^−1^ Dispase (Gibco) in RPMI 1640 medium plus 2% FCS (45 min, gentle shaking). Samples were filtered (70-μm filter, Corning) and mononuclear cells were isolated on a 40–80% Percoll (Cytiva). Lamina propria lymphocytes were recovered from the 40:80% interface and washed twice with cold DPBS.

### Cell isolation and flow cytometric analyses

Single-cell suspensions were generated by forcing organs through 70-μm sieves. Cell suspensions were blocked with PBS containing 5 μg ml^−1^ anti-CD16/CD32 (2.4G2) and stained (20–30 min, on ice) with fluorophore-conjugated antibodies or reagents, unless stated otherwise. For analysis of surface molecules, cells were stained with the antibodies: anti-CD45 (30-F11, BD Bioscience Pharmingen), EpCAM (G8.8, BioLegend), anti-M cell antibody (NKM-16-2-4, Miltenyi), *Ulex* *europaeus* Agglutinin I (UEA-1, Vector Laboratories), CD19 (ID3, BD Horizon), CD3ε (145^−^2C11, BD Horizon), CD45R (RA3B2, WEHI Antibody Facility), CD98 (RL388, BioLegend), CD38 (90, eBiociences) and CD95 (Jo2, BD OptiBuild). Live cells were identified by exclusion after staining with a fixable viability dye (BD Biosciences or BioLegend). Intracellular staining of DCLK1 was performed by fixing cells with 4% paraformaldehyde, then permeabilization with Perm/Wash buffer (BD Biosciences) and addition of unconjugated rabbit polyclonal to DCLK1 (LSBio), goat anti-rabbit IgG (H + L) cross-adsorbed secondary antibody conjugated with Pacific Blue, FITC or Alex Fluor 647. Intracellular staining of IgA and IgG was performed by fixing and permeabilizing cells before staining with anti-IgA (mA-6E1, eBiosciences) or anti-IgG (poly4053, Thermo Fisher). Cytokine staining was performed on unstimulated or stimulated (50 ng ml^−1^ phorbol 12-myristate 13-acetate and 500 ng ml^−1^ ionomycin) cells (4 h, 37 °C with 10 μg ml^−1^ Brefeldin A and GolgiPlug, BD Biosciences) in vitro in complete medium (RPMI 1640 medium containing 10% heat-inactivated FCS, 1 mM L-glutamine, 100 U ml^−1^ penicillin, 100 μg ml^−1^ streptomycin and 50 μM β-mercaptoethanol). Cells were stained for surface markers, then fixed and permeabilized using the Foxp3/Transcription Factor Staining Buffer Set (eBiosciences) and stained for various transcription factors (T-BET, GATA3 and RORγt) and cytokines (IL-17A and IL-22). Flow cytometry used an LSRFortessa X-20 or Canto10C (BD Biosciences) and FlowJo analysis software (BD Biosciences) and cells enumerated using counting beads (Invitrogen). Antibodies used in this study are outlined in Supplementary Table 7.

### Histological scoring of intestinal epithelial integrity

Intestinal tissues were fixed in 10% neutral buffered formalin and stained with hematoxylin and eosin, Alcian Blue Periodic Acid Schiff or Masson Trichome. Images were collected and analyzed with QuPath v.0.4.1 Bioimage Analysis software (https://qupath.github.io/)^55^. Sections were scored by researchers blinded to sample identity to generate a combined score of histological colitis (maximum of 25) via the assessment of parameters that reflect tissue damage and intestinal tissue severity^56^ (Supplementary Information).

### Single-cell RNA sequencing

Single-cell RNA sequencing was performed using Cel-Seq2 (ref. ^57^) or 10x GEM-X protocols. For Cel-Seq2 protocol, single CD45^−^EpCAM^+^SPI-B-tdTomato^*−*^ and CD45^*−*^EpCAM^+^SPI-B-tdTomato^+^ IECs from *Spib*^*tdTomato*^ mice were flow cytometrically sorted into 384-well plates (one cell per well) using a FACS Aria Fusion flow cytometer (BD Biosciences, USA). Index-sort data were collected at the time of cell deposition and libraries were generated using the Cel-Seq2 protocol^57^. Samples were pooled after first-strand complementary DNA synthesis, treated with Exonuclease 1 for 30 min, followed by bead cleanup. Second-strand synthesis was performed using NEB Next Second Strand Synthesis Module (NEB, E6111S) in a final reaction volume of 20 ml and Nucleo Mag NGS Cleanup and Size select magnetic beads (Macherey-Nagel, 7449970.5) were used for all DNA purification and size selection steps. Three biological replicates were prepared for all tissues (small and large intestine, *n* = 1 mouse per replicate; all Peyer’s patches were collected from each individual mouse then pooled from three mice for each replicate). Libraries were sequenced on an Illumina Nextseq 500 instrument with the following sequencing parameters: 14 cycles (read 1), 72 cycles (read 2) and a 6-cycle single index read.

scRNA-seq reads from Cel-Seq2 protocol were mapped to the GRCm38.p8 mouse genome and external RNA controls consortium (ERCC) spike-in sequences using the Subread aligner (v.2.4.3)^58^ and assigned to genes using scPipe (v.1.12.0)^59^ with GENCODE 18 primary assembly annotation. Gene counts were exported as a matrix using scPipe with unique molecular identifier aware counting. Quality control was performed in R (v.4.1.3)^60^ with Bioconductor (v.3.12)^61^ and were adapted from ‘Orchestrating Single Cell Analysis with R’^62^. Downstream analysis was performed in R with Seurat (v.4.2.0)^63^. Adaptive quality control thresholds were applied using scran^64^ based on sequencing depth, gene detection, and spike-in ERCC RNA reads from mitochondrial genes. Overall, 127 cells were excluded, leaving 4,361 cells and 3,262 tdTomato^+^ cells among them for downstream analysis. Protein-coding genes (20,000 genes) detected in at least one sample were retained for downstream analysis with a median of 319–1,619 genes detected per cell across groups and an overall range of 124–8,367 genes per cell. Seurat was then used for data normalization. A total of 2,000 highly variable genes were identified, and the normalized data were scaled based on them. We then performed principal-component (PC) analysis of the log counts of highly variable genes, retaining 20 PCs for downstream analysis. Batch correction was performed using RunHarmony^65^ followed by shared nearest-neighbor graph construction, FindClusters with Louvain algorithms using a standard Seurat analysis pipeline and UMAP. To annotate each cluster, we used FindAllMarkers in Seurat to find marker genes for each cluster. We also confirmed the expression level of marker genes of expected cell types from the literature. Differential expression analysis between M cells and Tuft cells, and between cells from different locations including Peyer’s patch, small and large intestine compared to other M cells without any stimulus was performed using FindMarkers. Pre-ranked Gene Set Enrichment Analysis was performed using Seurat-derived gene rankings to identify enriched Gene Ontology Biological Process pathways from the Molecular Signatures Database (MSigDB) with the top ten up- and downregulated pathways (false discovery rate <0.05).

For the 10x GEM-X protocol, CD45^*−*^EpCAM^+^SPI-B-tdTomato^+^ IECs were isolated from Peyer’s patches pooled from *Spib*^*tdTomato/+*^*Rorc*^*gfp/+*^ mice (*n* = 10) were flow cytometrically sorted and processed as a single GEM-X sample. The immune cell population from Peyer’s patches of *Spib*^*tdTomato/+*^*Rorc*^*gfp/+*^ mice consisted of CD45^+^lineage^*−*^CD90.2^+^RORγt-GFP^+^ cells (ILC3) that were mixed with CD45^+^GFP^−^cells and processed in a single well of a GEM-X 3’ Chip. Libraries were prepared using the Chromium GEM-X Single Cell 3′ Reagent kit v.4 and sequenced on an Illumina NextSeq.

The 10x GEM-X library reads were mapped to mm10 reference genome using Cell Ranger v.8.0.1. Seurat objects were created in R v.4.3.2 with RNA matrix files using Seurat (v.5.0.3)^66^. Quality control was performed by filtering cells with the following criteria: total RNA counts between 100 and 15,000 and total RNA feature counts between 200 and 7,500. The filtered data were further processed with DoubletFinder v.2.0.4 to remove doublets. Out of a total of 1,821 epithelial cells and 3,487 immune cells sequenced, 1,798 and 3,163 cells passed quality control, respectively. Cell cycle scores were assigned based on G2/M and S phase variability scores using the Seurat CellCycleScoring function. RNA gene expression data were normalized using SCTransform and PC analysis was performed on the SCTransformed Pearson residual matrix with 20 PCs using the RunPCA function in Seurat. UMAP was generated with the first 20 dimension reductions followed by unsupervised clustering using Seurat FindClusters function. UMAP plots were produced using Seurat DimPlot function with Viridis color scale. Marker genes were identified using Seurat FindAllMarkers function and DEGs between groups of cells or clusters were calculated using the FindMarkers function. DEGs were classified using an adjusted *P* value (Bonferroni-corrected) <0.01 and absolute log_2_ fold change >1. Gene expression plots were made with Seurat FeaturePlot function using normalized non-SCTransform RNA data.

### Cell–cell interaction analysis

Peyer’s patch epithelial and immune cell scRNA-seq data were merged and integrated using IntegrateLayers (RPCAIntegration) function of Seurat. Integrated object with epithelial and immune cells was used to predict intercellular communication with CellChat (v.2.1.2) package^33^ with default parameters.

### Microbiota sequencing

Feces were collected from animals that had been co-housed for at least 3 weeks and used litter mixed from cages. Fresh fecal pellets (50–300 mg) from individual mice (8–10 weeks old) were collected into one well of 96-deep-well plates (Axygen, No. AX-P-DW-20-C). DNA from feces was extracted using the QIAamp 96 Power Fecal QIAcube HT kit (QIAGEN, cat. no. 51531). Libraries were then generated with Nextera DNA Flex Library Preparation kit (Illumina, cat. no. 20018705) and indexed with IDT for Nextera DNA Unique Dual Indexes Set A-D (Illumina, cat. no. 20027213-6).

Nextera DNA FLEX libraries were pooled at equimolar amounts to create a sequencing pool. The pool was quantified, and quality control was performed with gel analysis, qubit measurement and qPCR. Libraries were prepared for sequencing on a NovaSeq6000 (Illumina) using NovaSeq6000, 2 × 150 bp paired-end chemistry. Library pools were sequenced to a target depth of 3 Gb per sample with a minimum of 2 Gb (~7–16 million paired-end reads).

### Fecal microbiota isolation and microbiota (m)FACS

Fresh feces were homogenized in 1 ml DPBS (Gibco). One 50 mm stainless steel bead (QIAGEN) was added to gently disrupt feces pellets in TissueLyser (QIAGEN). Samples were centrifuged (50*g*, 15 min, 4 °C) to remove large particles. Supernatants (50 μl) were pelleted (8,000*g*, 5 min, 4 °C), resuspended in staining buffer (100 μl) containing IgA (mA-6E1, eBiosciences), propidium iodide (PI) and SYTO 9 Green Fluorescent Nucleic Acid Stain (30 min on ice). Pellets were washed twice before flow cytometric analysis. Viable microbiota were distinguished by PI negative or low and SYTO9 high events using a LSRFortessa X-20 as previously described^67^.

### Fecal IgA analysis

Total IgA in fecal supernatants and serum was quantified by sandwich ELISA using goat anti-mouse IgA capture and HRP-conjugated detection antibodies (Southern Biotech). Reactions were developed with ABTS substrate (Roche) and absorbance was measured at 415 nm with reference correction at 492 nm using a SpectraMax microplate reader (Molecular Devices). To determine antigen-specific IgA antibody responses following *Salmonella* infection, an antigen-specific antibody ELISA was used. Clear polystyrene microplates (96-well, Corning) were coated with 10 μg ml^−1^ *Salmonella*. Typhimurium*-*derived lipopolysaccharide (BioXtra) resuspended in DPBS (overnight, 4 °C). Plates were then incubated (37 °C, 2 h) with fecal or serum supernatant. Goat anti-mouse IgA-HRP IgA antibody (Southern Biotech) was added and incubated (37 °C, 1.5 h) to detect the bound mouse IgA. ELISA reactions were developed for 10 min and the OD_415–492 nm_ was read. The negative control curve was generated with serum or fecal supernatant isolated from uninfected mice.

### Whole-mount imaging of intestinal epithelial cells

Intestinal tissues were cleaned, cut into 0.5–1-cm pieces and opened longitudinally. Tissues were fixed with 4% paraformaldehyde in PBS (40 min, room temperature), then incubated in blocking buffer (DPBS containing 1% BSA, 3% normal goat serum, 0.3% Triton-X100 and 0.1% sodium azide, 1 h, room temperature). EpCAM (CD326)-BV421, rabbit anti-mouse DCLK1 (unlabeled), chicken anti-GFP polyclonal antibody (unlabeled), CD11c-CF514, goat anti-RFP polyclonal antibody-CF568, CD3ε-Alexa Fluor 594 and B220-Alexa Fluor 647. Antibodies were diluted in working buffer (PBS containing 0.1% BSA, 0.3% normal goat serum and 0.3% Triton X-100) and incubated with tissues for 48 h (4 °C). Samples were washed twice with working buffer (15 min, room temperature), then incubated with goat anti-chicken IgY-Alexa Fluor Plus 488, goat anti-rabbit IgG–Alexa Fluor Plus 405 (1 h, room temperature). For RORγt staining, samples were incubated with rat anti-mouse RORγt for 48 h at 4 °C and subsequently stained with goat anti-rat IgG–Alexa Fluor 488, followed by donkey anti-goat IgG–Alexa Fluor 488, and samples were washed between each incubation. Tissues were washed before clearing using clearing-enhanced 3D (Ce3D)^68^. Sections were mounted with SlowFade Glass Antifade Mountant (Life Technologies). Images were captured using an LD LCI Plan-Apochromat ×25/0.8 Imm Corr DIC M27 objective on a Zeiss LSM 880 confocal microscope equipped with ZEN Black software (Zeiss). Multiple fluorescence channels were spectrally deconvolved using single-color reference spectra and unmixed using online fingerprinting. M cells were identified as EpCAM^+^DCLK1^*−*^SPI-B^+^ cells located in the epithelium of Peyer’s patches, whereas immune cells were characterized by their expression of CD3ε (T cell), B220 (B cell), CD11c (DC) and eGFP (ILC3). Three-dimensional reconstructions of intestinal tissue were generated and quantitated using Imaris Image Analysis software (v.9.3, Bitplane).

### Enumeration of markers

For radial shell analysis^69^, confocal images were quantified in FIJI/ImageJ v.2.1.0 using a custom radial shell macro (https://github.com/Wang-HJ-Cao/Radial-shell-analysis_PP). To account for variation in Peyer’s patch dome size, a circular or elliptical ROI was manually drawn around each dome and divided into four concentric shells representing 0–25%, 25–50%, 50–75% and 75–100% of the radial distance from the ROI center to its boundary. MFI was measured within each shell for RORγt, SPI-B, CD3, CD11c and B220. Data quality control and visualization were performed in R v.4.4.2 using the tidyverse package.

### Quantification and statistical analysis

Statistical analyses were calculated by performing a paired or unpaired two-tailed Student’s *t*-test or two-way analysis of variance using Prism v.10 software (GraphPad). Student’s *t*-tests for all single comparisons were two-tailed and unpaired and assumed Gaussian distribution and equivalent s.d. unless otherwise stated. Bar graphs display the mean ± s.e.m. For imaging analyses, multiple Peyer’s patches, domes or image fields collected from the same mouse were summarized at the mouse level before statistical analysis, unless otherwise stated. Each analysis is described in the Results. Statistical tests for each dataset are indicated in each figure legend; exact *n* values and *P* values are indicated. Statistical significance was considered at *P* < 0.05. No statistical method was used to predetermine sample size. No data were excluded from the analyses as long as the library and/or sequencing quality passed our criteria. The experiments were not randomized; however, animals were age- and sex-matched, and samples from all experimental groups were processed concurrently within each experiment to minimize batch-related bias. The investigators were not blinded to allocation during experiments and outcome assessment except for review of histological slides.

### Reporting summary

Further information on research design is available in the Nature Portfolio Reporting Summary linked to this article.

## Online content

Any methods, additional references, Nature Portfolio reporting summaries, source data, extended data, supplementary information, acknowledgements, peer review information; details of author contributions and competing interests; and statements of data and code availability are available at https://doi.org/10.1038/s41590-026-02606-3.

## Supplementary information

## Source data

## Data Availability

All data associated with this study are present in the paper or the Supplementary Information. RNA sequencing data associated with this publication have been deposited in the Gene Expression Omnibus repository with the accession number GSE280744. All other materials are available from the corresponding author on reasonable request. Source data are provided with this paper.

## Code availability

Code for radial shell analysis is available at https://github.com/Wang-HJ-Cao/Radial-shell-analysis_PP.
