# Author Correction: Peyer’s patch M cells organize an epithelial niche that sustains group 3 innate lymphoid cells and IL-22

**DOI:** 10.1038/s41590-026-02669-2

**Published:** 2026-09-01

**Authors:** Wang H. J. Cao, Yue You, Nancy Wang, M. Zeeshan Chaudhry, Huiyang Yu, Peter T. Bell, Ellesandra C. Noye, Renae Denman, Byungchul Lee, Abbey Waddington, Junpeng Ye, Jaring Schreuder, Qiutong Huang, Julie Tellier, Sophie Curio, James Santiago, Daniela Amann-Zalcenstein, Nicolas Jacquelot, Peter Hickey, Stephen L. Nutt, Cyril Seillet, Philip M. Hansbro, Verena C. Wimmer, Richard A. Strugnell, Zewen Kelvin Tuong, Matthew E. Ritchie, Gabrielle T. Belz

**Affiliations:** 1 The University of Queensland Frazer Institute, University of Queensland, Woolloongabba, Queensland Australia; 2 Walter and Eliza Hall Institute of Medical Research, Parkville, Victoria Australia; 3 Department of Medical Biology, University of Melbourne, Parkville, Victoria Australia; 4 Department of Microbiology and Immunology, The University of Melbourne at the Peter Doherty Institute for Infection and Immunity, Melbourne, Victoria Australia; 5 Department of Biochemistry & Molecular Biology; Department of Microbiology, Immunology & Infectious Diseases; Riddell Centre for Cancer Immunotherapy, Arnie Charbonneau Cancer Institute, Arthur J.E. Child Comprehensive Cancer Centre, Calvin, Phoebe, and Joan Snyder Institute for Chronic Diseases, Alberta Children’s Hospital Research Institute, Cumming School of Medicine, University of Calgary, Calgary, Alberta Canada; 6 Department of Immunology, University of Monash, Melbourne, Victoria Australia; 7 Centre for Inflammation, Centenary Institute and University of Technology Sydney, Faculty of Science, School of Life Sciences, Sydney, New South Wales Australia; 8 Ian Frazer Centre for Children’s Immunotherapy Research, Child Health Research Centre, Faculty of Medicine, The University of Queensland, Brisbane, Queensland Australia

**Keywords:** Innate lymphoid cells, Mucosal immunology

Correction to: *Nature Immunology* https://doi.org/10.1038/s41590-026-02606-3, published online 14 August 2026.

In the version of the article initially published, Matthew E. Ritchie’s surname appeared incorrectly (as Richie) and has now been amended in the HTML and PDF versions of the article.

