# Supplementary material for: Peyer’s patch M cells organize an epithelial niche that sustains group 3 innate lymphoid cells and IL-22
Source: Nat Immunol. 2026 Aug 14;27(9):1829–41. doi: 10.1038/s41590-026-02606-3 (PMC13506346; doi:10.1038/s41590-026-02606-3)
Supplement: Supplementary file 1 [file 41590_2026_2606_MOESM1_ESM.pdf]

nature immunology

Supplementary information

https://doi.org/10.1038/s41590-026-02606-3

Peyer's patch M cells organize an epithelial niche that sustains group 3 innate lymphoid cells and IL-22

In the format provided by the authors and unedited

Materials for

Peyer's patch M cells organise an epithelial niche that sustains group 3 innate lymphoid cells and IL-22

Cao *et al.*

The PDF file includes:

Supplementary Figures 1 to 5
Supplementary Movies 1, 2
Supplementary Tables 2, 3, 4
Supplementary Methods
Supplementary References

Other Supplementary Material for this manuscript includes the following:
Supplementary Table 1, 5, 6, 7, 8.

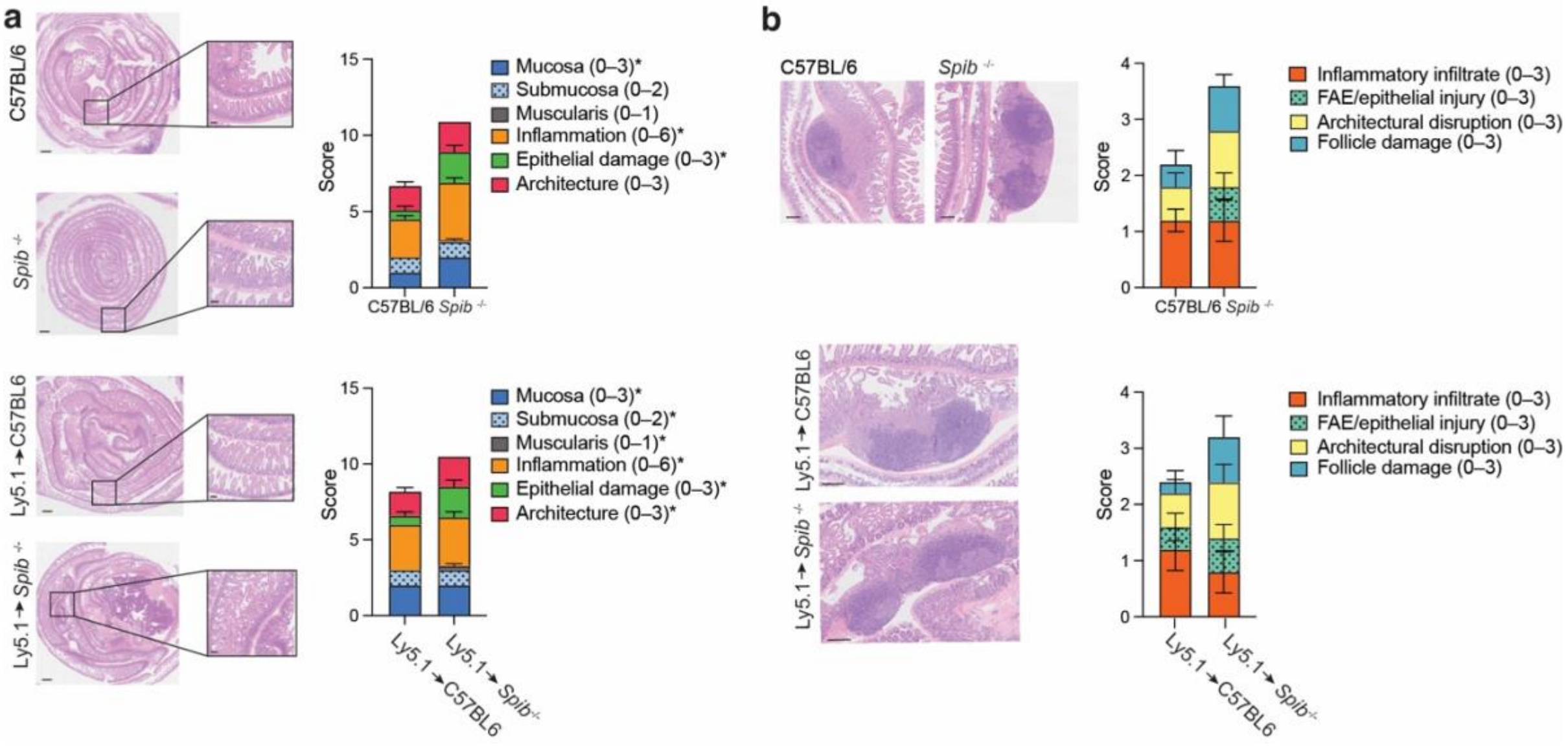

Supplementary Data Figure 1. SPI-B deficiency is associated with intestinal and Peyer's patch pathology at day 3 after *Salmonella* Typhimurium infection.

a,b, Representative H&E-stained sections and histological scoring of intestinal tissues collected on day 3 after *Salmonella enterica* serovar Typhimurium infection from control and SPI-B-deficient mice (top panels) or chimeric mice. Representative small intestinal sections (**a**) and Peyer's patch (**b**) from *Salmonella*-infected C57BL/6 and *Spib*$^{-/-}$ mice (upper panels) and chimeric C57BL/6 and *Spib*$^{-/-}$ mice 8 weeks after reconstitution with Ly5.1 bone marrow cells following irradiation (Ly5.1→C57BL/6 and Ly5.1→*Spib*$^{-/-}$) (lower panels). Inset shows regions at higher magnification. Small intestinal (SI) histopathology was blindly scored across tissue compartments including mucosa, submucosa, muscularis, inflammation, epithelial damage, and architectural disruption, and displayed as stacked cumulative scores. Peyer's patches were scored for inflammatory infiltrate, FAE/epithelial injury, architectural disruption, and follicle damage and displayed as stacked cumulative scores. Data are shown as mean ± s.e.m (n = 5 mice/genotype, 5 sections averaged per mouse) pooled from two independent experiments. Scale bars show 500 µm, 200 µm, and 100 µm for respective images. An unpaired two-tailed Student's t-test was used to test statistical significance (*, p<0.05).

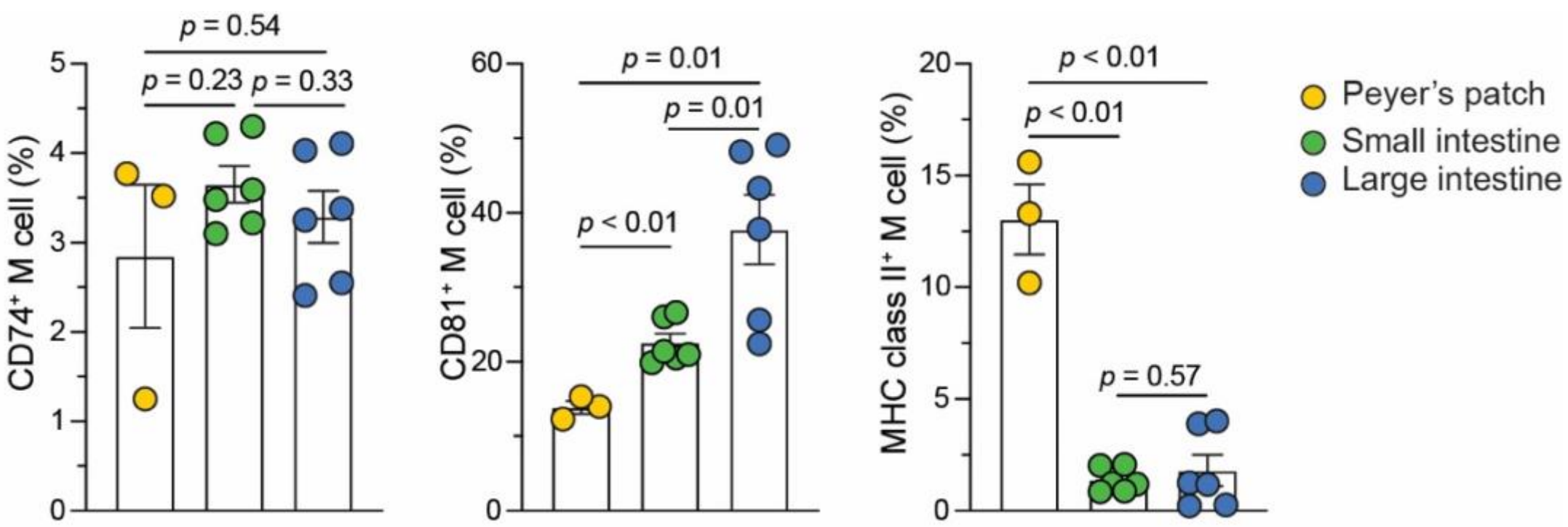

Supplementary Data Figure 2. Expression of antigen presentation-associated molecules by M cells at steady-state.

Flow cytometric quantification showing the frequency of $CD74^+$ (left), $CD81^+$ (middle) and MHC class II^+ (right) SPIB-$tdTomato^+DCLK1^-$ M cells in Peyer's patches, small intestine and large intestine of naïve *Spib*^{tdTomato} mice. Data show frequency for small intestine, large intestine, and pooled samples from Peyer's patch of individual mice and mean ± s.e.m. Data pooled from three independent experiments (n = 6 mice). p values were determined using an unpaired two-tailed Student's t test.

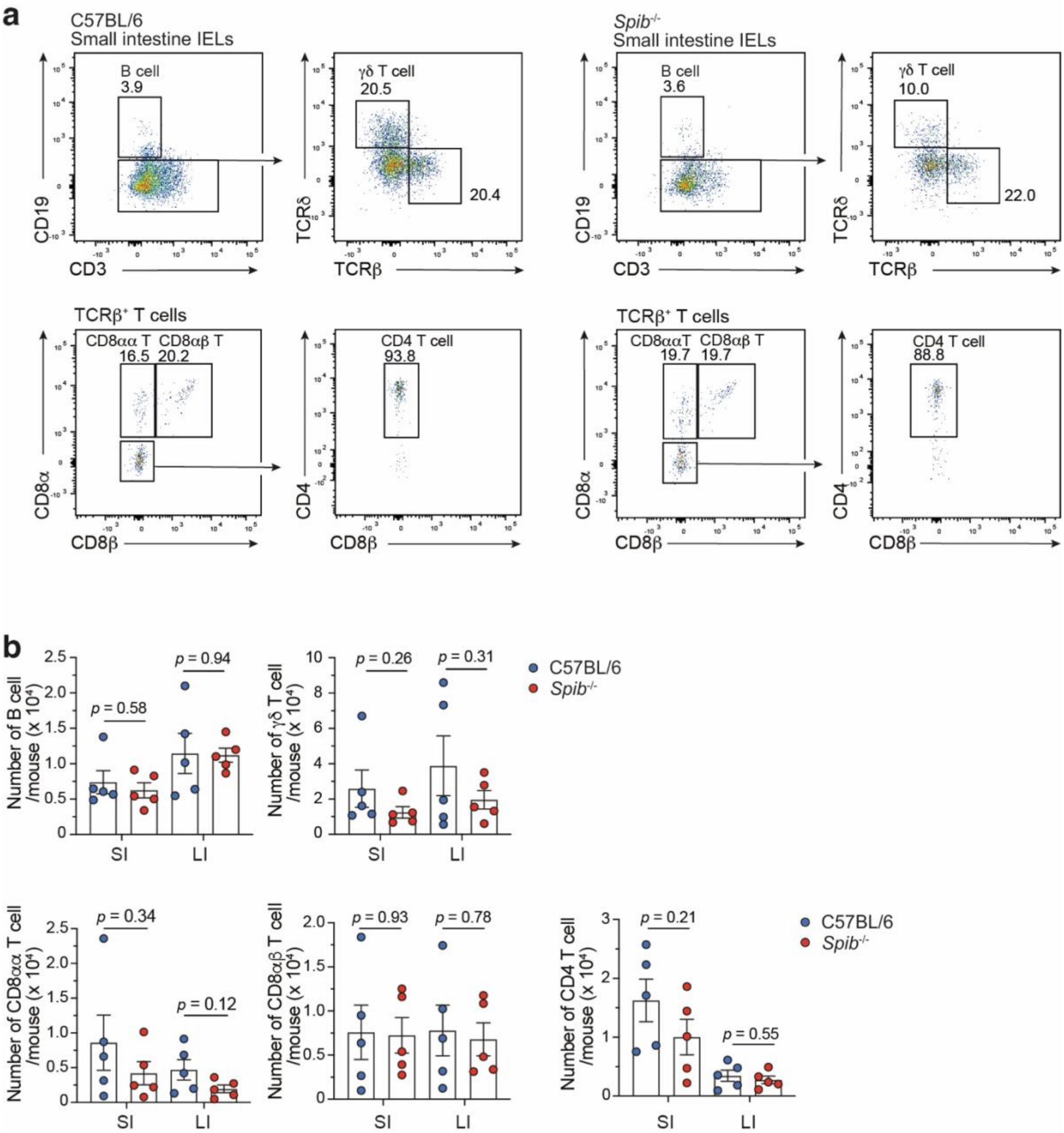

Supplementary Data Figure 3. IELs are maintained in the absence of SPI-B expression.

a, Flow cytometric plots showing the frequency of viable $CD45^{+}$ IEL populations isolated from the small intestine of C57BL/6 and *Spib*$^{-/-}$ mice. Data show representative plots from one of two independent experiments (n = 5 mice/genotype).

b, Flow cytometric quantification showing the total number of each IEL subset in the small intestine (SI) and large intestine (LI) of naïve C57BL/6 and *Spib*$^{-/-}$mice as in **a**. Data show individual values for each mouse and mean ± s.e.m. pooled from two experiments (n=5 mice/genotype). Statistical significance was determined using the unpaired two-tailed Student's t-test.

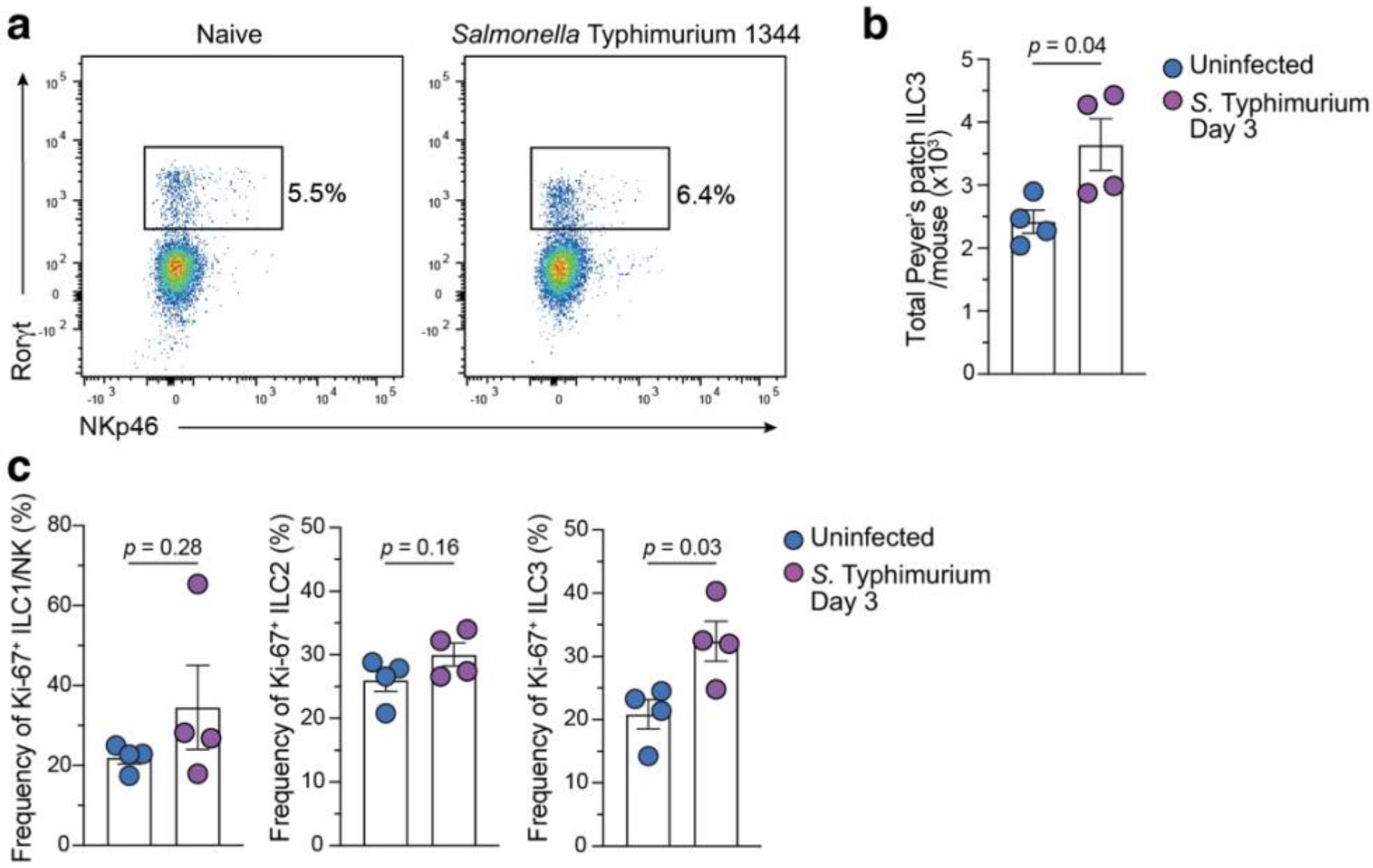

Supplementary Data Figure 4. Peyer's patch ILC3 abundance and proliferation following *Salmonella* Typhimurium infection.

a, Flow cytometric plots showing live lin$^-$CD45$^+$CD127$^+$CD90.2$^+$ cells isolated from the Peyer's patch of naive and *Salmonella enterica* serovar Typhimurium SL1344-infected C57BL/6 mice three days after infection.

b, Flow cytometric quantification showing the total number of ILC3 isolated from Peyer's patch of uninfected and *Salmonella*-infected mice as in **a**.

c, Flow cytometric quantification showing the frequency of Ki67$^+$ Peyer's patch ILC3s.

a-c, Data from one of two experiments shown as mean ± s.e.m. ($n = 4$ mice/genotype) Statistical significance in was determined using an unpaired two-tailed Student's t test (**b**,**c**).

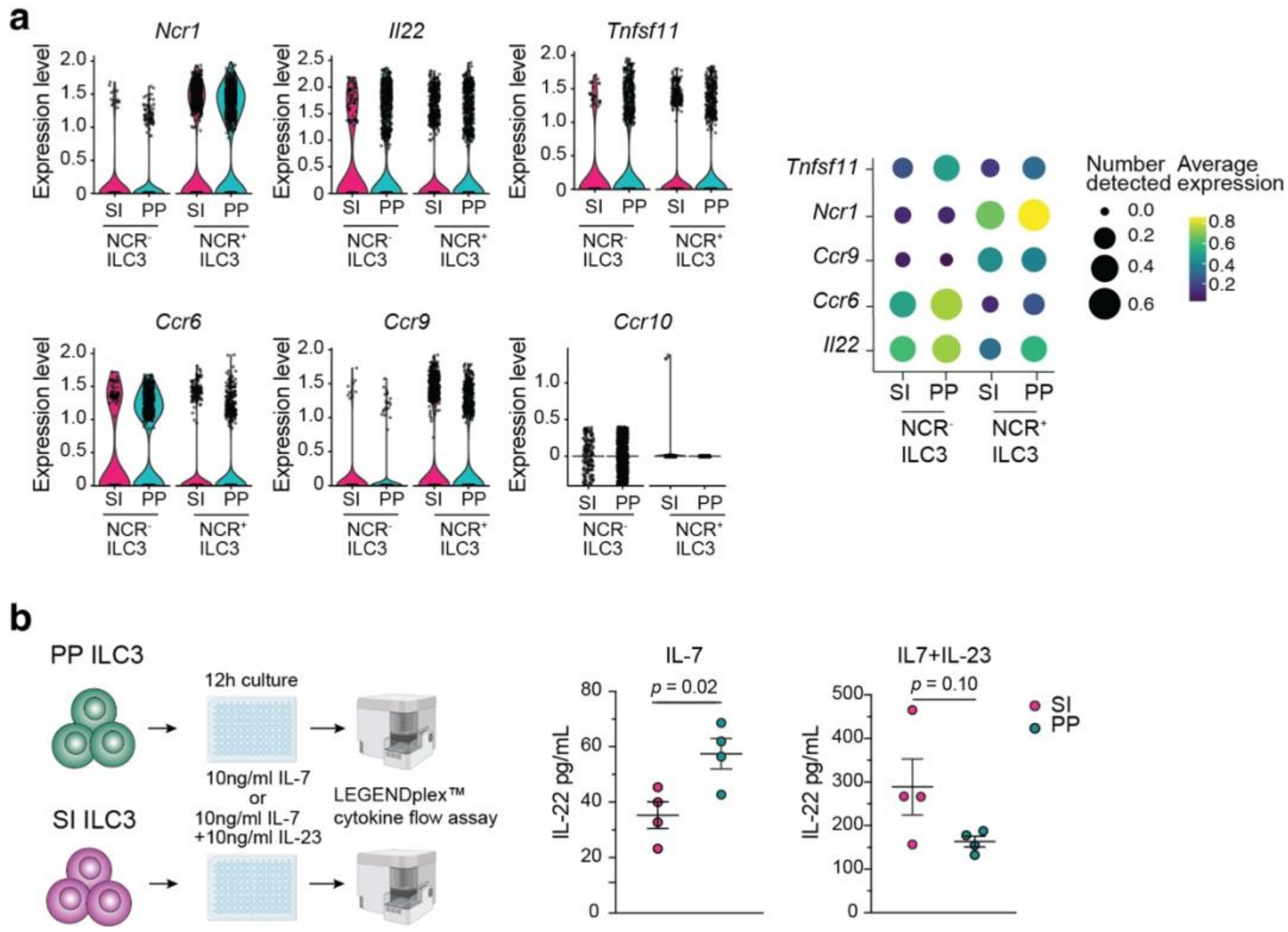

Supplementary Data Figure 5. Peyer's patch and small intestinal ILC3s show distinct transcriptional and functional profiles.

a, Violin plots (left) and dot plot (left) showing expression of selected genes in small intestine (SI) and Peyer's patch (PP) NCR^- ILC3 and NCR^+ ILC3 subsets. Data was re-analysed from GSE124880.[1]

b, Cytokine assay showing IL-22 production by purified Peyer's patch and small intestinal ILC3 (3. 5 $\times 10^3$ cells/well) isolated from naïve C57BL/6 mice and cultured for 12 h in the presence of IL-7 or IL-7+IL-23. IL-22 was measured in the culture supernatants using LEGENDplex^{TM} mouse IL-22 cytokine assay. Data pooled from two independent experiments and show mean ± s.e.m. (n = 4 mice), p values were determined using an unpaired two-tailed Student's t test.

Supplementary Movie 1. Multiple follicles in the small intestinal Peyer's patch show the discrete positioning of T cells (CD3, red), B cells (purple), intestinal epithelial cells (grey), tuft cells (blue), RORγt^{+} innate cells (green) and SPI-B^{+} epithelial and immune cells. Time 17 secs.

Supplementary Movie 2. Spatial organisation of small intestinal Peyer's patch. The different layers and positioning of epithelial cells (blue), SPI-B^{+} epithelial and immune cells (yellow), RORγt^{+} innate cells (green) and DCLK1^{+} tuft cells (magenta) are exposed through the planes of the cleared tissues. Time 25 secs.

Supplementary Tables

Supplementary Table 1. Supplementary Table 1. Analysis of marker gene expression on intestinal SPIB⁺ IECs used for cluster annotation.

Marker genes were identified by differential gene expression analysis comparing each $SPIB^+$ clusters to all the other clusters. Statistical significance was assessed using the Wilcoxon Rank Sum test, and *p*-values were adjusted for multiple testing using the Benjamini–Hochberg method. For each gene, the $\log_2$ fold change ($\log_2FC$) was calculated to indicate the magnitude of expression difference. Genes with an adjusted $p < 0.05$ and positive $\log_2FC$ were considered significantly enriched in the $SPIB^+$ IEC cluster and used for annotation.

Supplementary Table 2. Recovery of cells for scRNA-seq analysis from different intestinal sites.

	**Condition**		
**Tissue**	**Naive**	***S. entericia* Typhimurium**	***C. rodentium***
**SPI-B-tdTomato⁺ IECs**			
Peyer's patch	327	178	61
Small intestine	480	384	485
Large intestine	533	349	465
**SPI-B-tdTomato⁻ IECs**			
Small intestine	283	0	89
Large intestine	159	0	70
Total cells recovered	1,782	911	1,170

Supplementary Table 3. Enumeration of cells in scRNA-seq clusters across all conditions.

**Cluster annotation**	**Number of cells***	**Proportion of indicated population**
**SPI-B-tdTomato⁺ IECs**		
M_1	242	7.38%
M _2	151	4.60%
M_3	56	1.71%
Tuft_1	900	27.44%
Tuft_2	793	24.18%
Tuft_3	596	18.17%
Tuft_4	220	6.71%
B cells	131	3.99%
unknown	171	5.21%
**SPI-B-tdTomato⁻ IECs**		
Crypt stem cell	115	30.3%
Enterocytes	194	51.2%
Goblet cell	70	18.5%

* passed sequencing quality control

Supplementary Table 4. Enumeration of cells in scRNA-seq clusters of Peyer's patch of naïve *Spib*$^{tdTomato/+}$*Rorc(γt)*$^{gfp/+}$mice.

Cluster annotation	Number of cells*	Proportion of total cells recovered in each compartment
**Peyer's patch epithelial cells**		
M_1	131	8.69%
M_2	252	16.71%
Tuft_1	386	25.6%
Tuft_2	96	6.37%
Tuft_3	416	27.59%
Tuft_4	227	15.05%
**Peyer's patch immune cells**		
Activated B cells	890	28.68%
Follicular B cells	692	22.30%
Memory-like B cells	554	17.85%
GC B cells	234	7.54%
T cells	265	8.54%
NCR^{+} ILC3	309	9.96%
NCR^{-} ILC3	159	5.12%

* passed sequencing quality control

Supplementary Table 5. Analysis of marker gene expression comparing M cells and Tuft cells.
The analysis was carried out using the Wilcoxon Rank Sum test, comparing gene expression levels between the two cell populations. Each gene entry includes the $\log_2$ fold change ($\log_2$FC), raw *p*-value, and adjusted *p*-value (Benjamini–Hochberg correction). Genes with statistically significant differences (*adjusted p* < 0.05) highlight molecular features that define the unique identity and function of each cell type.

Supplementary Table 6. Pathway enrichment analysis of M Cells versus enterocytes using PrGSEA.
Pre-ranked gene set enrichment analysis (PrGSEA) was used to perform pathway analysis of differentially expressed genes between M-cell clusters and enterocytes. Using Seurat's FindMarkers function, genes were ranked according to the -log10(*p*-value) multiplied by the sign of the log foldchange. The ranked list of genes was used for PrGSEA using gene sets from Gene Ontology Biological Processes, accessed through the Molecular Signatures Database. The results are filtered for the top 10 up or down pathways in the respective contrast based on the Normalised Enrichment Score (NES) and a false discovery rate (FDR)<0.05.

Supplementary Table 7. List of antibodies used in this study.
Supplementary Table 8. List of primers used in this study.

Supplementary Methods

Histological scoring of intestinal epithelial integrity

Related to Extended Data Fig. 1b. Intestinal tissues were fixed in 10% neutral buffered formalin and stained with hematoxylin and eosin, Alcian Blue Periodic Acid Schiff or Masson Trichome. Images were collected and analyzed with QuPath 0.4.1 Bioimage Analysis software (https://qupath.github.io/)[2]. Sections were scored by researchers blinded to sample identity to generate a combined score of histological colitis (maximum of 25) *via* the assessment of parameters that reflect tissue damage and intestinal tissue severity[3]. Tissues were assessmented for crypt architecture (0-normal to 5-large ulcers), crypt abscesses (0=none to 3 ≥10 crypt abscesses), tissue or epithelial damage (0-none to 3-extensive mucosal damage), goblet cell depletion (0=normal to 3≥50% depletion), inflammatory cell infiltration (0-occasional infiltration to 3-transmural infiltration), and shape, position and compactness of glands, and Paneth cell expansion. Crypt length (0–4) was measured using QuPath 0.4.1 Bioimage Analysis software (https://qupath.github.io/).[2] This scoring system produces a combined score of histological colitis (maximum of 25) *via* the assessment of parameters that reflect tissue damage and intestinal tissue severity.[3]

Quantitative PCR Analysis

Related to Extended Data Fig. 1c, 3d. For quantitative PCR (qPCR) analyses, samples were processed according to the cell type examined in each experiment. Live SPI-B-tdTomato^{+} lymphocytes were isolated by FACS sorting, and RNA was purified using RNeasy Plus Mini Kits (Qiagen). Intestinal epithelial cells were isolated by dissociation in 5 mM EDTA in HBSS containing 2% FCS at 37°C for 50 min, followed by RNA extraction using TRIzol™ (Invitrogen) according to the manufacturer's instructions. For Peyer's patches were directly lysed in TRIzol™ and homogenised using TissueLyser (Qiagen). RNA was quantified by NanoDrop Lite spectrophotometry (Thermo Scientific). cDNA was synthesised from total RNA using Bio-Rad iScript reagents according to the manufacturer's instructions. Quantitative PCR was performed using SYBR Green chemistry on either a Bio-Rad CFX384 or QuantStudio 7 real-time PCR platform. Primers used were listed in Supplementary Table 8.

Faecal sequencing analysis

Related to Extended Data Fig. 1d. α-diversity was measured using the Shannon index and species or phylum richness that were compared using a standard *t* test. Data was rarefied to 2,135,150 reads. Taxonomic profiles were analyzed using supervised and unsupervised multivariate methods. Profiles were ordinated using the unsupervised methods Non-Metric Multidimensional Scaling (NMDS) and PC Analysis (PCoA). The supervised methods Adonis and Redundancy analysis were used to assess if variance in microbial community composition could be attributed to the study condition. NMDS, PCoA and Adonis were run on Bray-Curtis dissimilarities. Differential abundance of microbial species was examined by ANOVA or linear mixed effect regression (LMER) of centred log-ratio transformed relative abundances, Fisher's exact test and/or ALDEx2 (on species or phylum read counts). The LMER p values correspond to a nested model test of the significance of including the corresponding fixed effect. ALDEx2 uses subsampling (Bayesian sampling) to estimate the underlying technical variation. For each sample, centre log-ratio transformed data was compared across study groups and computed p values were corrected for multiple testing using the Benjamini–Hochberg procedure.

Faecal IgA Analysis

Related to Extended Fig.1i,j,l, 2d Total IgA in faecal supernatants and serum was quantified by sandwich ELISA. Ninety-six-well plates (Corning) were coated overnight at room temperature with goat anti-mouse IgA antibody (10 µg/ml; SouthernBiotech, 1040-01) in DPBS. Samples diluted in blocking buffer (DPBS containing 0.05% Tween-20, 1% FCS and 0.6% skim milk powder) were incubated overnight at room temperature, followed by goat anti-mouse IgA–HRP antibody (SouthernBiotech) for 4 h. Reactions were developed for 45 min in the dark using ABTS substrate (0.54 mg/ml; Roche) containing 0.03% hydrogen peroxide in citrate buffer (0.1M Citric acid and 0.1 M Trisodium Citrate·$2H_2O$). Absorbance was measured at 415 nm with reference correction at 492 nm using a SpectraMax microplate reader (Molecular Devices). IgA concentrations were calculated from serial dilutions of purified mouse IgA (SouthernBiotech).

pIgR staining and quantification

Related to Extended Data Fig. 1e. The small intestine was cleaned with 1×PBS, rolled into a Swiss roll, and fixed in 4% paraformaldehyde (PFA) overnight at 4 °C. The tissue was then embedded in OCT compound and stored at −80 °C until cryosectioning into 5 µm sections. Sections were stained with rabbit polyclonal anti-pIgR antibody (1:200; Invitrogen, Cat.#PA5-76848) and anti-EpCAM-Alexa Fluor 594 antibody (1:300; BioLegend, Cat.#118222), followed by goat anti-rat IgG-Alexa Fluor 647 secondary antibody (1:400; Invitrogen, Cat. #A-21247). Images were acquired using a Zeiss LSM 980 confocal microscope and analysed with Imaris software (Bitplane, Oxford Instruments). Epithelial cells were identified as EpCAM$^+$ DAPI$^+$ cells, and mean fluorescence intensity (MFI) of pIgR was quantified.

Single cell RNA-seq analysis of stromal and neuronal cells in Peyer's patch Peyer's patch neuronal cells

Related to Extended Data Fig. 7a,b. Published single-nuclei RNA-seq (snRNA-seq) data[4], available *via* the Single Cell Portal (https://singlecell.broadinstitute.org/single_cell), were mined. Raw count matrices, gene annotation files, and cell barcodes were imported into R and processed with the Seurat package. Low-quality or artifactual nuclei were removed: cells with fewer than 200 detected genes, more than 5,000 detected genes, or greater than 10% mitochondrial transcripts were excluded. Gene expression counts were normalized, and highly variable features were identified using the variance-stabilizing transformation (2,000 genes). Data were scaled with ScaleData while regressing out mitochondrial transcript proportion. Dimensionality reduction was performed with principal component analysis (PCA) on the highly variable genes, and the first 20 principal components were used for downstream steps. A shared nearest-neighbor (SNN) graph was constructed, and unsupervised clustering was conducted using the Louvain algorithm. Neuronal and glial clusters were subsequently examined for *Spib* expression.

Peyer's patch stromal cells

Related to Extended Data Fig. 7c-e. Published single-cell RNA-seq data were mined *via* ArrayExpress (accession number E-MTAB-9162; 10x Chromium (10x Genomics))[5]. In this dataset, Peyer's Patches from adult *Ccl19*^{Cre}*R26*^{YFP} and *Col6a1*^{Cre}*R26*^{YFP} mice were digested, stained and sorted for fibroblasts as defined by Live, CD31$^-$ cells that expressed PDPN$^+$ and/or VCAM$^+$. Raw gene-cell count matrices were generated using the 10x Genomics Cell Ranger pipeline (version 9.0) and imported into R using the Seurat package. Quality control (QC) metrics were calculated for each cell, including the number of detected genes (nFeature_RNA), the total UMI count (nCount_RNA), and the percentage of reads mapping to mitochondrial genes. Cells were retained if within median ±2.5 median absolute deviations (MAD) across

both nCount_RNA and nFeature_RNA, and if mitochondrial gene content was below the > 2.5 MAD threshold. Cells expressing cell cycle proliferation markers (*Top2a, Mki67, Cenpf, Pclaf*) or contamination markers characteristic of non-stromal lineages (*Lyve1, Cldn5, Ptprc, Cd79a, Cd79b, Vil1, Fcer1g, Gfap, S100b*) were excluded from downstream analysis. Cells with high *Cdh5* expression (> 95th percentile) were removed to eliminate any residual contamination.

Single-cell RNA-seq datasets were normalized and variance-stabilized followed by integration with the Seurat SCTransform workflow. Integration features were selected with SelectIntegrationFeatures using the top 3,000 variable genes across datasets. The datasets were prepared for integration with PrepSCTIntegration, and integration anchors were identified using FindIntegrationAnchors with SCTransform normalization. Data were integrated with IntegrateData using the SCT assay. Dimensionality reduction was performed using principal component analysis (PCA) on the top 30 principal components (PCs). Cell-cell relationships were computed with FindNeighbors using the first 30 PCs. Downstream analysis included normalization, scaling, dimensional reduction with principal component analysis (PCA) and UMAP and graph-based clustering utilizing functions from the Seurat package. Clusters were characterized based on the expression of canonical marker genes as well as unbiased cluster markers inferred from the Wilcoxon test, as implemented in the FindMarkers function of the Seurat package.

IL-22 production assay

Related to Supplemental Data Figure 2. Measurement of IL-22 production by Peyer's patch and small-intestinal ILC3. ILC3 were flow cytometrically purified from Peyer's patches and small intestine of naïve C57BL/6 mice. Sorted ILC3 (5,000 cells per well) were cultured for 12 h with IL-7 alone or IL-7+IL-23. Culture supernatants were harvested, and IL-22 concentrations were quantified using a custom LEGENDplex™ assay (BioLegend) based on the Mouse T Helper Cytokine Panel.

References

1. Xu, H. *et al.* Transcriptional Atlas of Intestinal Immune Cells Reveals that Neuropeptide alpha-CGRP Modulates Group 2 Innate Lymphoid Cell Responses. *Immunity* **51**, 696–708 e699 (2019).
2. Bankhead, P. *et al.* QuPath: Open source software for digital pathology image analysis. *Sci Rep* **7**, 16878 (2017).
3. Wang, R. *et al.* Gut microbiota shape the inflammatory response in mice with an epithelial defect. *Gut Microbes* **13**, 1–18 (2021).
4. Drokhlyansky, E. *et al.* The Human and Mouse Enteric Nervous System at Single-Cell Resolution. *Cell* **182**, 1606–1622 e1623 (2020).
5. Prados, A. *et al.* Fibroblastic reticular cell lineage convergence in Peyer's patches governs intestinal immunity. *Nature Immunology* **22**, 510–519 (2021).
