# Supplementary material for: Peyer’s patch M cells organize an epithelial niche that sustains group 3 innate lymphoid cells and IL-22
Source: Nat Immunol. 2026 Aug 14;27(9):1829–41. doi: 10.1038/s41590-026-02606-3 (PMC13506346; doi:10.1038/s41590-026-02606-3)
Supplement: Supplementary file 2 [file 41590_2026_2606_MOESM2_ESM.pdf]

nature portfolio

Corresponding author(s): Gabrielle T. Belz

Last updated by author(s): 28/06/2026

Reporting Summary

Nature Portfolio wishes to improve the reproducibility of the work that we publish. This form provides structure for consistency and transparency in reporting. For further information on Nature Portfolio policies, see our Editorial Policies and the Editorial Policy Checklist.

Statistics

For all statistical analyses, confirm that the following items are present in the figure legend, table legend, main text, or Methods section.

n/a	Confirmed	
☐	☒	The exact sample size (*n*) for each experimental group/condition, given as a discrete number and unit of measurement
☐	☒	A statement on whether measurements were taken from distinct samples or whether the same sample was measured repeatedly
☐	☒	The statistical test(s) used AND whether they are one- or two-sided<br>*Only common tests should be described solely by name; describe more complex techniques in the Methods section.*
☒	☐	A description of all covariates tested
☐	☒	A description of any assumptions or corrections, such as tests of normality and adjustment for multiple comparisons
☐	☒	A full description of the statistical parameters including central tendency (e.g. means) or other basic estimates (e.g. regression coefficient) AND variation (e.g. standard deviation) or associated estimates of uncertainty (e.g. confidence intervals)
☐	☒	For null hypothesis testing, the test statistic (e.g. *F*, *t*, *r*) with confidence intervals, effect sizes, degrees of freedom and *P* value noted<br>*Give P values as exact values whenever suitable.*
☒	☐	For Bayesian analysis, information on the choice of priors and Markov chain Monte Carlo settings
☒	☐	For hierarchical and complex designs, identification of the appropriate level for tests and full reporting of outcomes
☐	☒	Estimates of effect sizes (e.g. Cohen's *d*, Pearson's *r*), indicating how they were calculated

Our web collection on statistics for biologists contains articles on many of the points above.

Software and code

Policy information about availability of computer code

Data collection	FlowJo (version 9 and 10, Becton Dickinson, USA), Zen software (Zeiss, Germany), Imaris (version 9.3, Bitplane, USA), Subread aligner (v2.4.3), scPipe (v1.12.0), R (version 4.1.3), Bioconductor (version 3.12), Seurat (4.2.0), RunHarmony, MAST, limma (3.50.3), and SpectraMax Microplate Reader software (Molecular Devices LLC, USA).
Data analysis	Data analysis in this study involved several software tools for processing and analyzing different types of data. FlowJo (version 9 and 10) was used to analyze flow cytometry data and quantify specific cell populations. Zen Software (Zeiss, Germany) facilitated image acquisition and analysis for microscopy data. Imaris (version 9.3, Bitplane, USA) was employed for 3D image reconstruction and cell quantification. For radial shell analysis, confocal images were quantified in FIJI/ImageJ v2.1.0 using a custom radial shell macro (https://github.com/Wang-HJ-Cao/Radial-shell-analysis_PP). Subread Align (v2.4.3) mapped RNA sequencing reads to the reference genome, while scPipe (v1.12.0) processed scRNA-seq data for gene assignment. R (version 4.1.3) and related packages such as Seurat (4.2.0) were used for scRNA-seq data analysis, clustering, and visualization. Bioconductor (version 3.12) provided tools for quality control, and RunHarmony corrected for batch effects in scRNA-seq data. MAST and limma (3.50.3) were used for differential gene expression and Gene Ontology analysis, respectively. Finally, SpectraMax Microplate Reader Software (Molecular Devices LLC, USA) was used to analyze ELISA data. Custom R scripts were written for data preprocessing, statistical analysis, and visualization.

For manuscripts utilizing custom algorithms or software that are central to the research but not yet described in published literature, software must be made available to editors and reviewers. We strongly encourage code deposition in a community repository (e.g. GitHub). See the Nature Portfolio guidelines for submitting code & software for further information.

Data

Policy information about availability of data

All manuscripts must include a data availability statement. This statement should provide the following information, where applicable:

- Accession codes, unique identifiers, or web links for publicly available datasets
- A description of any restrictions on data availability
- For clinical datasets or third party data, please ensure that the statement adheres to our policy

Data have been deposited in the Gene Expression Omnibus repository with the accession number GSE280744. All other data generated or analysed in this study are included in the published version and supplementary information files.

Research involving human participants, their data, or biological material

Policy information about studies with human participants or human data. See also policy information about sex, gender (identity/presentation), and sexual orientation and race, ethnicity and racism.

Reporting on sex and gender	*Use the terms sex (biological attribute) and gender (shaped by social and cultural circumstances) carefully in order to avoid confusing both terms. Indicate if findings apply to only one sex or gender; describe whether sex and gender were considered in study design; whether sex and/or gender was determined based on self-reporting or assigned and methods used. Provide in the source data disaggregated sex and gender data, where this information has been collected, and if consent has been obtained for sharing of individual-level data; provide overall numbers in this Reporting Summary. Please state if this information has not been collected. Report sex- and gender-based analyses where performed, justify reasons for lack of sex- and gender-based analysis.*
Reporting on race, ethnicity, or other socially relevant groupings	*Please specify the socially constructed or socially relevant categorization variable(s) used in your manuscript and explain why they were used. Please note that such variables should not be used as proxies for other socially constructed/relevant variables (for example, race or ethnicity should not be used as a proxy for socioeconomic status). Provide clear definitions of the relevant terms used, how they were provided (by the participants/respondents, the researchers, or third parties), and the method(s) used to classify people into the different categories (e.g. self-report, census or administrative data, social media data, etc.) Please provide details about how you controlled for confounding variables in your analyses.*
Population characteristics	*Describe the covariate-relevant population characteristics of the human research participants (e.g. age, genotypic information, past and current diagnosis and treatment categories). If you filled out the behavioural & social sciences study design questions and have nothing to add here, write "See above."*
Recruitment	*Describe how participants were recruited. Outline any potential self-selection bias or other biases that may be present and how these are likely to impact results.*
Ethics oversight	*Identify the organization(s) that approved the study protocol.*

Note that full information on the approval of the study protocol must also be provided in the manuscript.

Field-specific reporting

Please select the one below that is the best fit for your research. If you are not sure, read the appropriate sections before making your selection.

☒ Life sciences ☐ Behavioural & social sciences ☐ Ecological, evolutionary & environmental sciences

For a reference copy of the document with all sections, see nature.com/documents/nr-reporting-summary-flat.pdf

Life sciences study design

All studies must disclose on these points even when the disclosure is negative.

Sample size	Sample sizes in this study were determined based on prior research conducted in our laboratory, following the principle of ensuring sufficient numbers of mice per group to provide statistically meaningful results. This approach accounts for potential sources of variability, such as differences in cell isolation during the digestion process, and ensures the reliability and reproducibility of the findings.
Data exclusions	No data points were excluded.
Replication	All experiments, except RNA (including single-cell sequencing), were performed at least twice. The presented data were successfully replicated.
Randomization	Age- and sex-matched mice were allocated to groups based on the experimental treatment (no randomisation).
Blinding	Blinding was not performed in this study as data analysis in strictly quantitative (and not subjective) and data acquisition occurred based on fixed protocols. Thus, introduction of investigator bias is unlikely.

Reporting for specific materials, systems and methods

We require information from authors about some types of materials, experimental systems and methods used in many studies. Here, indicate whether each material, system or method listed is relevant to your study. If you are not sure if a list item applies to your research, read the appropriate section before selecting a response.

Materials & experimental systems

n/a	Involved in the study
☐	☒ Antibodies
☒	☐ Eukaryotic cell lines
☒	☐ Palaeontology and archaeology
☐	☒ Animals and other organisms
☒	☐ Clinical data
☒	☐ Dual use research of concern
☒	☐ Plants

Methods

n/a	Involved in the study
☒	☐ ChIP-seq
☐	☒ Flow cytometry
☒	☐ MRI-based neuroimaging

Antibodies

Antibodies used

Antibodies:

- Anti-mouse B220- Alexa Fluor 647 (clone RA3-6B2) - Biolegend, Cat#103229
- Anti-mouse CD11b-PE-Cy7 (clone M1/70) - eBioscience, Cat#25-0112-82
- Anti-mouse CD11c-PE-Cy7 (clone HL3) - BD Pharmingen, Cat#558079
-Anti-mouse CD11c (unlabeled, clone N418) - Antibody Facility (WEHI), N/A
- Anti-mouse CD117 (C-kit)-APC (clone 2B8) - eBioscience, Cat#46-0333-82
- Anti-mouse CD138-PE-Cy7 (clone 281-2) - BioLegend, Cat#142513
- Anti-mouse CD16/CD32 (clone 2.4G2 purified) - Antibody Facility (WEHI), N/A
- Anti-mouse CD19-BUV395 (clone 1D3) - BD Horizon, Cat#563557
- Anti-mouse CD23-BUV737 (clone B3B4) - BD Optibuild, Cat#749668
- Anti-mouse CD3ε-BUV-395 (clone 145-2C11) - BD Horizon, Cat#563565
- Anti-mouse CD3ε- Alexa Fluor 594 (clone 17A2) - Biolegend, Cat#100240
- Anti-mouse CD326 (EpCAM)-BV421 (clone G8.8) - Biolegend, Cat#118225
- Anti-mouse CD326 (EpCAM)-PE-Cy7 (clone G8.8) - BioLegend, Cat#118216
- Anti-mouse CD38-PeCp-Cy5.5 (clone 90) - BioLegend, Cat#102721
- Anti-mouse CD4-PE-Cy7 (clone GK1.5) - eBioscience, Cat#25-0041-82
- Anti-mouse CD45-BV786 (clone 30-F11) - BD Horizon, Cat#564225
- Anti-mouse CD45.1-FITC (clone A20) - BD Bioscience Pharmigen, Cat#561871
- Anti-mouse CD45.2-BV786 (clone 104) - BD Horizon, Cat#563686
- Anti-mouse CD45R(B220)-APC-Cy7 (clone RA3-6B2) - eBioscience, Cat#17-0452-82
- Anti-mouse CD49a-Alex Fluor 647 (clone Ha31/8) - BD Pharmingen, Cat#562113
- Anti-mouse CD49b-eFluor 450 (clone DX5) - eBioscience, Cat#48-5971-82
- Anti-mouse CD64-BV605 (X54-5/7.1) - Biolegend, Cat#563414
- Anti-mouse CD8α-PE-Cy7 (clone 53-6.7) - eBioscience, Cat#25-0081-82
- Anti-mouse CD95-BV711 (clone Jo2) - BD OptiBuild, Cat#740716
- Anti-mouse CD98-APC-Fire750 (clone RL388) - BioLegend, Cat#128216
- Anti-mouse/human DCLK1 (polyclonal) - LSBio, Cat#LS-C100746-200
- Anti-mouse Flt3-Biotin (clone A2F10) - eBioscience, Cat#13-1351-85
- Anti-mouse/human Gata3-BV711 (clone L50-823) - BD Horizon, Cat#565449
- Anti-mouse GP2-Alexa Fluor 488 (clone 2F11-C3) - MBL International Corporation, Cat#D278-A48
- Anti-mouse I-A/I-E (MHC-II)-bv711 (clone M5/114.15.2) - BD Horizon, Cat#47-5961-82
- Anti-mouse IgA (clone mA-6E1) - eBiosciences, Cat#12-4204-82
- Anti-mouse IgA (polyclonal) - Southern Biotech, Cat#10400
- Anti-mouse IgA-HRP (polyclonal) - Southern Biotech, Cat#1040-05
- Anti-mouse IgG-BV421 (polyclonal) - BioLegend, Cat#405414
- Anti-mouse IgM- APC-eFluor 780 (clone II/41) - eBioscience, Cat#47-5790-82
- Anti-mouse Ig κ-FITC (clone 187.1) - BD Bioscience Pharmigen, Cat#550003
- Anti-mouse Ig λ1, λ2, λ3-FITC (clone R26-46) - BD Bioscience Pharmigen, Cat#553434
- Anti-mouse IL-17A-PerCP-eFluor710 (clone TC11-18H10) - eBioscience, Cat#560220
- Anti-mouse IL-22-APC (clone IL22JOP) - eBioscience, Cat#17722282
- Anti-mouse IL-5-PE (clone TRFK5) - eBioscience, Cat#12705281
- Anti-mouse IL7Rα-PE-Cy7 (clone SB-199) - eBioscience, Cat#25-1273-82
- Anti-mouse Ly-6A/E (Scal1)-V500 (clone D7) - BD Horizon, Cat#561228
- Anti-mouse Ly6D-eFluor 450 (49-H4) - eBioscience, Cat#48-5974-80
- Anti-mouse-Ly6G/C (Gr-1)-PE-Cy7 (clone RB6-8C5) - BD Pharmingen, Cat#552985
- Anti-mouse Microfold-APC (clone NKM-16-2-4) - Miltenyi Biotec, Cat#130-102-150
- Anti-mouse NK1.1-BV650 (clone PK136) - BD Horizon, Cat#D278-A48
- Anti-mouse NKp46-PE-Cy7 (clone 29A1.4) - BioLegend, Cat#137617
- Anti-mouse RANKL-Biotin (clone IK22/5) - BioLegend, Cat#737694

- Anti-mouse Rorγt-BV421 (clone Q31-378) - BD Horizon, Cat#562894
- Anti-mouse Siglec-F-BV421 (clone E50-2440) - BD Horizon, Cat#565934
- Anti-mouse Siglec-H-PerCP-eFluor710 (clone eBio440c) - eBioscience, Cat#565934
- Anti-mouse TCR β-APC-eFlour780 (clone H57-597) - eBioscience, Cat#47-5961-82
- Anti-mouse-TER-119-PE-Cy7 (clone TER-119) - eBioscience, Cat#25-5921-82
-Anti-mouse- CD218a (IL-18a)-PerCP/eFluor710 (clone P3TUNYA) -eBioscience, Cat#46-5183-82
-Anti-mouse-CD186 (CXCR6)-BV421 (clone SA051D1) - BioLegend, Cat#151109
-Rabbit anti-mouse pIgR (polyclonal) - Invitrogen, Cat#PA5-76848
-Anti-mouse-Ki-67-Alexa Fluor 488 (clone SolA15) - BD Pharmingen, Cat#53-5698-82

Secondary Antibodies:

- Chicken anti-GFP polyclonal antibody - Invitrogen, Cat#A10262
- Goat anti-chicken IgY (H+L) cross-adsorbed secondary antibody-Alexa Fluor Plus 405 (polyclonal) - Invitrogen, Cat#A32931
- Goat anti-rabbit IgG (H+L) – Alex Fluor 488 (polyclonal) - Invitrogen, Cat#A11034
- Goat anti-rabbit IgG (H+L) – Alex Fluor 647 (polyclonal) - Invitrogen, Cat#A21245
- Goat anti-rabbit IgG (H+L) – Pacific Blue (polyclonal) - Invitrogen, Cat#P-10994
- Goat anti-RFP polyclonal antibody (polyclonal) - Rockland, Cat#200-101379
- Goat anti-rabbit IgG (H+L) highly cross-adsorbed secondary antibody-Alexa Fluor Plus 405 (polyclonal) - Invitrogen, Cat#A48254
- Streptavidin-APC-eFluor780 - eBioscience, Cat#47-4317-82

Validation

All antibodies were titrated and validated using appropriate controls.

Animals and other research organisms

Policy information about studies involving animals; ARRIVE guidelines recommended for reporting animal research, and Sex and Gender in Research

Laboratory animals

The animals used in the study included C57BL/6 (Ly5.2+/+), Ly5.1 (B6.SJL-Ptprca Pepcb/BoyJ) (Ly5.1+/+), F1 (Ly5.1 × C57BL/6) (Ly5.1+ Ly5.2+), Spib-tdTomato (SpibT2A-tdTomato-IRES-CreERT2), Spib KO (Spib-/-), Blimp-1-GFP (Prdm1+/GFP), and Rorγt-GFP (Rorc(γt)+/GFP). Both male and female mice were used at 6-16 weeks old age. All mice were bred and maintained under specific pathogen-free conditions at the animal facility of of the WEHI or the University of Queensland.

Wild animals

No wild animals were used in this project.

Reporting on sex

Both, male and female mice were used.

Field-collected samples

None

Ethics oversight

The University of Queensland Animal Ethics Committee

Note that full information on the approval of the study protocol must also be provided in the manuscript.

Plants

Seed stocks

Report on the source of all seed stocks or other plant material used. If applicable, state the seed stock centre and catalogue number. If plant specimens were collected from the field, describe the collection location, date and sampling procedures.

Novel plant genotypes

Describe the methods by which all novel plant genotypes were produced. This includes those generated by transgenic approaches, gene editing, chemical/radiation-based mutagenesis and hybridization. For transgenic lines, describe the transformation method, the number of independent lines analyzed and the generation upon which experiments were performed. For gene-edited lines, describe the editor used, the endogenous sequence targeted for editing, the targeting guide RNA sequence (if applicable) and how the editor was applied.

Authentication

Describe any authentication procedures for each seed stock used or novel genotype generated. Describe any experiments used to assess the effect of a mutation and, where applicable, how potential secondary effects (e.g. second site T-DNA insertions, mosiacism, off-target gene editing) were examined.

Flow Cytometry

Plots

Confirm that:

☒ The axis labels state the marker and fluorochrome used (e.g. CD4-FITC).

☒ The axis scales are clearly visible. Include numbers along axes only for bottom left plot of group (a 'group' is an analysis of identical markers).

☒ All plots are contour plots with outliers or pseudocolor plots.

☒ A numerical value for number of cells or percentage (with statistics) is provided.

Methodology

Sample preparation

For FACS analysis, tissues were enzymatically digested to generate single-cell suspensions (described in Materials & Methods section , which were then filtered, and cell viability was assessed. Specific populations were isolated based on surface markers using flow cytometry. Intracellular staining involved fixing and permeabilizing cells using the Foxp3/Transcription Factor Staining Buffer (Thermo Fisher) or BD Perm/Wash buffer (BD Biosciences) , followed by incubation with antibodies targeting intracellular proteins. Cells were analyzed using flow cytometry for marker expression.

For imaging, cells or tissue sections were mounted and imaged with a fluorescence or confocal microscope to assess marker expression.

For RNA sequencing, RNA was extracted from isolated c)lls, processed into libraries, and sequenced to evaluate gene expression and identify molecular pathways.

For ELISA analysis, samples were collected and centrifuged to separate plasma or supernatants, which were then diluted as necessary. The samples were processed according to the manufacturer's instructions for the specific ELISA kit used. Absorbance was measured to quantify analyte concentrations, with internal controls included for quality assurance.

For qPCR, samples were homogenized and RNA was extracted to be transcribed into cDNA.

For histology, tissues were fixed in 4% neutral buffered formalin (NBF), embedded in paraffin, then sectioned and stained as required, including H&E.

Instrument

The following instruments were used in the study: Flow cytometry was performed using the LSRFortessa X-20 and BD FACS Aria™ Fusion flow cytometer (BD Biosciences, USA). Imaging was carried out on the Zeiss LSM 880 NLO and Zeiss LSM 980 confocal microscope (Zeiss, Germany). ELISA readings were taken using a SpectraMax Microplate Reader (Molecular Devices LLC, USA). Single-cell sorting for RNA sequencing was performed using the BD FACS Aria™ Fusion flow cytometer (BD Biosciences, USA). Intestinal tissue was embedded in OCT compound and cryosectioned, with sections imaged using a Zeiss LSM 880 NLO confocal microscope (Zeiss, Germany). Quantitative PCR was performed using SYBR Green chemistry on either a Bio-Rad CFX384 or QuantStudio 7 real-time PCR platform.

Software

Flow cytometry data were analyzed using FlowJo™ v10 (BD Biosciences, USA). Imaging data were processed and analyzed using Zen Blue (Zeiss, Germany) and ImageJ (NIH, USA). ELISA data were analyzed using SoftMax Pro (Molecular Devices LLC, USA). RNA sequencing data were analyzed using STAR (Dobin et al., 2013) for alignment, followed by DESeq2 (Love et al., 2014) for differential expression analysis. Statistical analysis was performed using GraphPad Prism v9 (GraphPad Software, USA) and R (R Foundation for Statistical Computing, Austria). Data visualization was performed using GraphPad Prism v9 (GraphPad Software, USA) and ggplot2 (Wickham, 2016).

Cell population abundance

The gating strategy and the indicative frequencies/abundance of populations are presented in both the main and supplementary figures.

Gating strategy

M cells : CD45-EpCAM+SPIB-tdTomato+DCLK1- cells.
Tuft cells: CD45-EpCAM+DCLK1+.
IgA plasma cells : CD45+CD138+IgA+ cells or Cd45+ Blimp-GFP+ IgA+ cells.
NK/ILC1: CD45+NK1.1+NKp46+, negative for KLRG1, TER119, B220, CD19, CD3, TCRβ, CD11b.
ILC2: CD45+NK1.1-GATA3+, negative for KLRG1, TER119, B220, CD19, CD3, TCRβ, CD11b.
ILC3: CD45+NK1.1-Rorγt+, negative for KLRG1, TER119, B220, CD19, CD3, TCRβ, CD11b.

☒ Tick this box to confirm that a figure exemplifying the gating strategy is provided in the Supplementary Information.
